# Thymidylate Synthase Genotype-Directed Chemotherapy for Patients with Gastric and Gastroesophageal Junction Cancers

**DOI:** 10.1371/journal.pone.0107424

**Published:** 2014-09-18

**Authors:** Laura W. Goff, Nilay Thakkar, Liping Du, Emily Chan, Benjamin R. Tan, Dana B. Cardin, Howard L. McLeod, Jordan D. Berlin, Barbara Zehnbauer, Chloe Fournier, Joel Picus, Andrea Wang-Gillam, Wooin Lee, A. Craig Lockhart

**Affiliations:** 1 Department of Medicine, Vanderbilt-Ingram Cancer Center, Nashville, Tennessee, United States of America; 2 Department of Pharmaceutical Sciences, College of Pharmacy, University of Kentucky, Lexington, Kentucky, United States of America; 3 Department of Medicine, Siteman Cancer Center, Washington University School of Medicine, St. Louis, Missouri, United States of America; 4 Moffitt Cancer Center, Tampa, Florida, United States of America; 5 Centers for Disease Control and Prevention, Atlanta, Georgia, United States of America; Kyushu University Faculty of Medical Science, Japan

## Abstract

**Background:**

Retrospective studies indicate associations between *TSER* (thymidylate synthase enhancer region) genotypes and clinical outcomes in patients receiving 5-FU based chemotherapy, but well-controlled prospective validation has been lacking.

**Methods:**

In this phase II study (NCT00515216 registered through ClinicalTrials.gov, http://clinicaltrials.gov/show/NCT00515216), patients with “good risk” *TSER* genotypes (at least one TSER*2 allele) were treated with FOLFOX chemotherapy to determine whether prospective patient selection can improve overall response rates (ORR) in patients with gastric and gastroesophageal junction (GEJ) cancers, compared with historical outcomes in unselected patients (estimated 43%).

**Results:**

The ORR in genotype-selected patients was 39.1% (9 partial responses out of 23 evaluable patients, 95% CI, 22.2 to 59.2), not achieving the primary objective of improving ORR. An encouraging disease control rate (DCR, consisting of partial responses and stable diseases) of 95.7% was noted and patients with homozygous TSER*2 genotype showed better tumor response.

**Conclusions:**

In this first prospective, multi-institutional study in patients with gastric or GEJ cancers, selecting patients with at least one TSER*2 allele did not improve the ORR but led to an encouraging DCR. Further studies are needed to investigate the utility of selecting patients homozygous for the TSER*2 allele and additional genomic markers in improving clinical outcomes for patients with gastric and GEJ cancers.

**Trial Registration:**

ClinicalTrials.gov NCT00515216

## Introduction

Cancers of the gastric cardia, gastroesophageal junction (GEJ) and distal esophagus have been rapidly increasing in incidence in the past decades, especially in patients younger than 40 years of age [1]. Unfortunately, 80–90% of newly diagnosed patients present with regional or distant metastatic disease and even with optimal therapy, the median survival in these patients is less than 1 year and survival at 5 years is essentially zero [2], [3]. Recent advances in our capacity to detect and target specific molecular lesions in cancer cells and to obtain genetic information from tumor tissues and patients allow for selection of optimal therapies, improving the outcomes for these aggressive cancers. For example, targeting HER-2 expressing gastroesophageal tumors with trastuzumab led to an improvement in the survival of patients with advanced disease [4]. However, for the majority of patients selection of initial therapy remains largely empiric as most of the regimens have similar response rates and median survival [3], [5]. Thus, there is a clear need for approaches that can guide treatment selection for the most effective regimens.

5-fluorouracil (5-FU) is one of the most commonly prescribed chemotherapy agents. It has demonstrated preclinical synergy with oxaliplatin in a variety of tumor types and this combination has demonstrated clear efficacy in treating patients with gastric and GEJ cancers [6]–. Thymidylate synthase (TS, encoded by the *TYMS* gene) is the critical enzyme in DNA synthesis and repair and it is the primary target for 5-FU and other folate-based antimetabolites [9]. Overexpression of TS has been linked to clinical resistance to TS-targeted agents including 5-FU [10]. In turn, genetic polymorphisms involving the promoter region of the *TYMS* gene, specifically, the number of tandem repeat sequences in the TS enhancer region (*TSER* - the 28-nucleotide G/C-rich sequence in the 5′-untranslated region) have been shown to be important determinants of tumoral TS expression [11]–[15]. The two most common *TSER* alleles are the two tandem repeats (TSER*2, allelic frequency = 0.2∼0.4) and the three tandem repeats (TSER*3, allelic frequency = 0.6∼0.8) [15].

Retrospective studies in colorectal cancer have demonstrated that individuals with TSER*3/*3 genotype had a significantly lower response rate and poorer outcomes to 5-FU compared with those with at least one TSER*2 allele [11], [16]–[18]. Additional retrospective analyses of TS expression or *TSER* genotypes in relation to clinical outcomes in patients with gastric cancers replicated these findings [19], [20]. More recently, findings from the first prospective study evaluating the utility of *TSER* genotypes in directing neoadjuvant chemoradiation for patients with rectal carcinoma demonstrated that patients treated with 5-FU-based chemoradiotherapy according to their *TSER* genotypes had improved tumor downstaging [21]. Together, these studies provide ample evidence that TS expression status and/or *TSER* genotyping may be useful in selecting patients who are likely to respond to treatment with 5-FU or its analogues.

This Phase II study was designed to prospectively select patients with “good risk” *TSER* genotypes (i.e. TSER*2/*2 or *2/*3) and treat them with a standard 5-FU-based regimen (FOLFOX; 5-FU, leucovorin, oxaliplatin) in order to improve clinical outcomes in patients with gastric and GEJ cancers. The primary end point of this study was to determine whether *TSER* genotype-directed chemotherapy would result in an improved overall response rate (ORR, 60% or higher) compared to historical control response rates in non-genotype selected patients (estimated 43%). The secondary end points were to retrospectively assess whether other genetic variations, in particular, additional polymorphic loci in the *TYMS* gene and other genes involved in the disposition and response to the FOLFOX regimen would influence the response in the treated patients.

## Materials and Methods

### Ethics Statement

The trial was done in accordance with the Declaration of Helsinki and ICH Good Clinical Practice. The study protocol was approved by the Vanderbilt University and Washington University Institutional Review Boards and all participants provided their written informed consent to participate in this study. The study was registered through ClinicalTrials.gov (Identifier: NCT00515216). The protocol for this trial and supporting TREND checklist are available as supporting information; see Checklist S1 and Protocol S1. The trial started (first patient enrolled) in June 2008 and ended (last patient completed the clinical study) in October 2010.

### Eligibility

Adult (≥18 years) patients who had histologically or cytologically confirmed adenocarcinoma of the stomach or GEJ and had received no prior therapy for metastatic disease were eligible. Subjects could have received prior neoadjuvant or adjuvant therapy as long as the disease-free interval was longer than 6 months. Eligible patients had Eastern Cooperative Oncology Group (ECOG) performance status ≤2, adequate organ and bone marrow function, and an ability to understand and willingness to sign written informed consent. Patients with known active brain metastases, HIV on anti-retroviral therapy or other uncontrolled intercurrent illnesses were excluded.

### Study Design and Treatment

**Figure 1:**
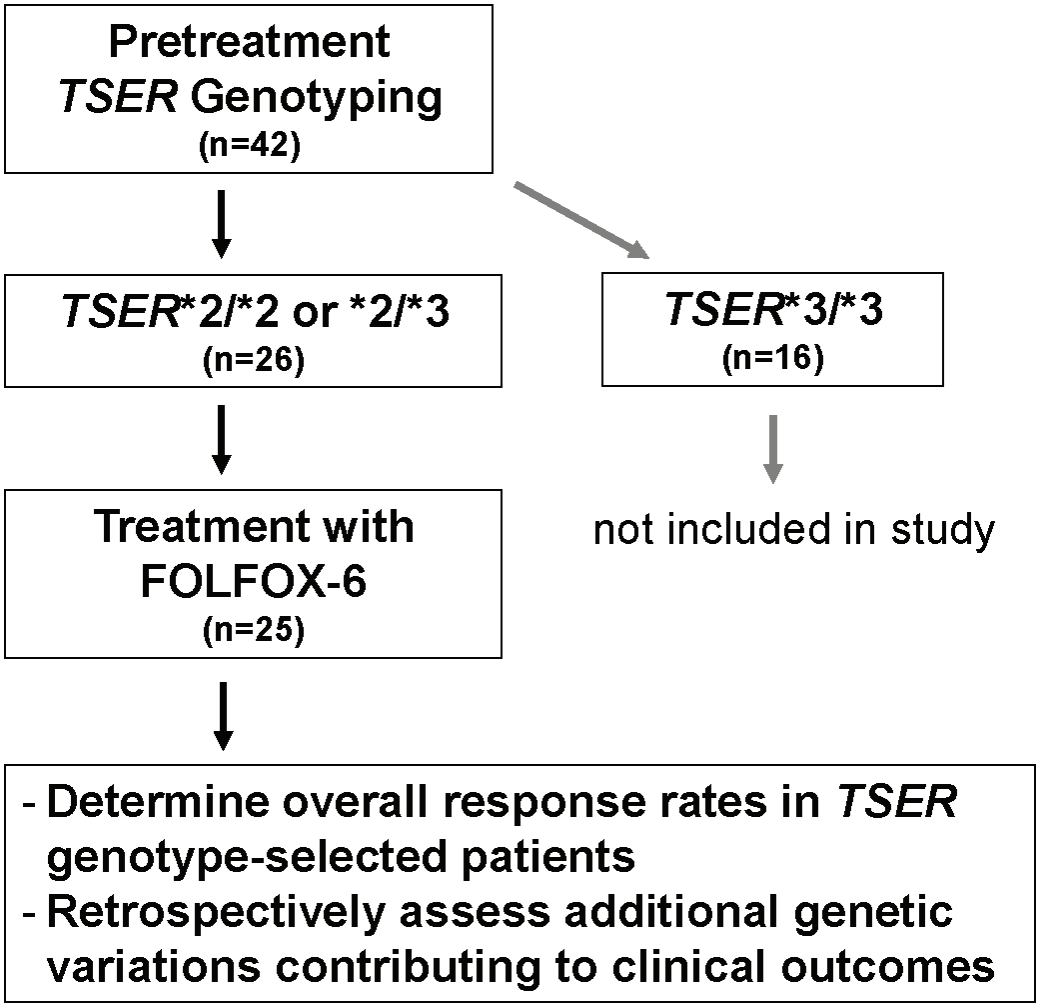
A schematic diagram of the current phase II study design.

This was an open-label, non-randomized, investigator-initiated multi-center study involving prospective genotyping (study flow chart shown in Figure 1). Potentially eligible patients underwent a blood draw for *TSER* genotyping. Patients with TSER*3/*3 genotype were not included in study treatment. Patients with TSER*2/*2 or TSER*2/*3 genotypes received the modified FOLFOX-6 treatment consisting of oxaliplatin 85 mg/m^2^ and leucovorin 400 mg/m^2^ given over 2 hours along with 5-FU 400 mg/m^2^ given as an intravenous push over 5 minutes, followed by 5-FU 2,400 mg/m^2^ given as an intravenous infusion of 46 hours. This treatment was repeated every 2 weeks in the absence of unacceptable adverse events or disease progression.

### Assessment of Efficacy and Toxicity

The patients were reevaluated for response every 8 weeks. In addition to a baseline scan, confirmatory scans were performed not less than 4 weeks following initial documentation of objective response. Tumor response and progression were evaluated using Response Evaluation Criteria in Solid Tumors (RECIST). Partial response (PR) was defined as at least 30% decrease in the sum of the longest diameter (LD) of target lesions, taking as reference the baseline sum LD. Progressive disease (PD) was defined as at least a 20% increase in the sum of the LD of target lesions, taking as reference the smallest sum LD recorded since the treatment started or the appearance of one or more new lesions. Stable disease (SD) was defined as neither sufficient shrinkage to qualify for PR nor sufficient increase to qualify for PD. Toxicities were graded according to the National Cancer Institute Common Terminology Criteria for Adverse Events (NCI-CTCAE), version 3.0. Dose modifications were made depending on the type and severity of toxicities observed.

### Retrospective Genotyping Analyses

Retrospective genotyping analyses by restriction fragment length polymorphism (RFLP) were performed for two additional loci in the *TYMS* gene (G>C SNP within the second 28-bp tandem repeat of the 3R allele, rs34743033; 1494delTTAAAG in the 3′-UTR, rs34489327) and five loci in additional genes reported to be associated with response and toxicity to the FOLFOX regimen (*ERCC1*, c.354C>T, rs11615; *ERCC2*, c.2251A>C, rs13181; *GSTP1*, c.313A>G, rs1695; *XRCC1*, c.1196G>A, rs25487; and *MDR1*, c.3435C>T, rs1045642). Briefly, regions encompassing the respective polymorphisms were PCR amplified (Platinum PCR Supermix, Invitrogen) and subjected to restriction enzyme digest to yield the diagnostic fragmentation patterns (detailed conditions provided in Table S1 in File S1) [13], [22]–[30]. The digested and undigested products from each patient sample were visualized on 3% agarose gel along with positive controls whose genotypes were verified by direct sequencing.

### Statistical Analyses

The primary objective of this study was to test whether selection of patients according to *TSER* genotypes would improve treatment response rates compared to the response rates previously reported in an unselected population. Sample size estimation was based on the assumed response rate of 43% with this regimen in an unselected population and improving the response rate to a minimum of 60% based on the retrospective data [19], [20]. An Optimum MinMax two-stage accrual design was employed [31]. In the initial step, 45 eligible patients were to be entered into the study, with a final accrual goal of 75 if ≥20 responses were observed in the first group. This design provides 90% statistical power to detect a difference of 17% with a two-sided significance level of <0.05. Unfortunately, the current study had to be terminated earlier than the initial stopping point due to insufficient funding. For 23 patients evaluable for tumor responses, univariate associations between genotypes and tumor response (PR and SD) were evaluated using Fisher’s exact test. Overall survival (OS) and progression free survival (PFS) were analyzed using Kaplan-Meier models and associations between genotypes and patient survival were assessed using log-rank tests. No sub-analyses were performed for potential associations between genotypes and toxicities since they were not pre-specified in our analysis plans. All statistical analyses were performed using R package (version 3.0.2).

## Results

### Patient characteristics

Patient baseline characteristics are listed in Table 1. Between June 2008 and October 2010, 42 patients with gastric and GEJ cancers were screened for their *TSER* genotypes; 26 patients (63.4%) had good risk genotypes (TSER*2/*2 or *2/*3) and enrolled onto the trial. One patient with a good risk genotype withdrew consent and was not treated on study. The majority of patients had gastric cancer (73%) and good performance status (69% ECOG PS of 1 or better). The median age of the participants was 56 years and a majority (62%) was male.

**Table 1:** Baseline clinical and demographic characteristics of enrolled patients (n = 26).

Characteristic	Patients (n = 26)	
	No	%
*TSER* genotypes		
TSER*2/*2	5	19
TSER*2/*3	21	81
Gender		
Male	16	62
Female	10	38
Age (years)		
median	56	
range	26–81	
Ethnicity		
Caucasians	23	88
African Americans	2	8
Not reported	1	4
ECOG performance status		
0	5	19
1	13	50
2	8	31
Primary tumor type		
Gastric	19	73
Gastroesophageal junction (GEJ)	7	27
Prior neoadjuvant or adjuvant therapy (>6 months)	2	8

### Treatment and toxicities

Twenty five patients received at least one dose of study treatment with the modified FOLFOX-6 regimen. A total of 128 cycles were administered, with a median of 5.5 cycles per patient (range, 0.5–15 cycles). The occurrence and the incidence of the main toxicities are reported in Table 2. The toxicities experienced by the study participants were within the expected range for patients receiving treatment with FOLFOX. The most common toxicities were hematologic with grade 3 and 4 neutropenia, leukopenia and anemia recorded in 8 out of 25 (32%), 4 out of 25 (16%), and one out of 25 (4%) patients, respectively. Only two out of 25 patients experienced grade 3 gastrointestinal toxicity and no participants had grade 4 gastrointestinal toxicity. Neurotoxicity was common and was observed in 44% (grade 1 in 20%, grade 2 in 16% and grade 3 in 8%) of the patients. Fatigue was also common and was observed in 52% (grade 1 in 40%, grade 2 in 4% and grade 3 in 8%) of the patients. No treatment-related deaths were reported.

**Table 2:** Toxicities observed in the treated patients (n = 25).

Toxicity	Grade 1		Grade 2		Grade 3		Grade 4	
	No	%	No	%	No	%	No	%
Leukopenia (total WBC)	4	16	5	20	4	16	0	0
Neutropenia	3	12	2	8	4	16	4	16
Lymphopenia	3	12	4	16	1	4	0	0
Anemia	13	52	6	24	1	4	0	0
Thrombocytopenia	9	36	3	12	0	0	0	0
Nausea	12	48	2	8	1	4	0	0
Vomiting	3	12	3	12	1	4	0	0
Diarrhea	8	32	0	0	0	0	0	0
Mucositis/stomatitis	3	12	1	4	0	0	0	0
Taste alteration (dysgeusia)	8	32	0	0	0	0	0	0
Vision-blurred vision	3	12	0	0	0	0	0	0
Allergic reaction/hypersensitivity	0	0	1	4	0	0	0	0
Rash: hand-foot skin reaction	2	8	0	0	0	0	0	0
AST, SGOT	8	32	2	8	1	4	0	0
ALT, SGPT	10	40	2	8	0	0	0	0
Neuropathy: sensory	5	20	4	16	2	8	0	0
Fatigue (asthenia, lethargy, malaise)	10	40	1	4	2	8	0	0

### Response to FOLFOX regimen, recurrence and survival and TSER genotypes

**Figure 2:**
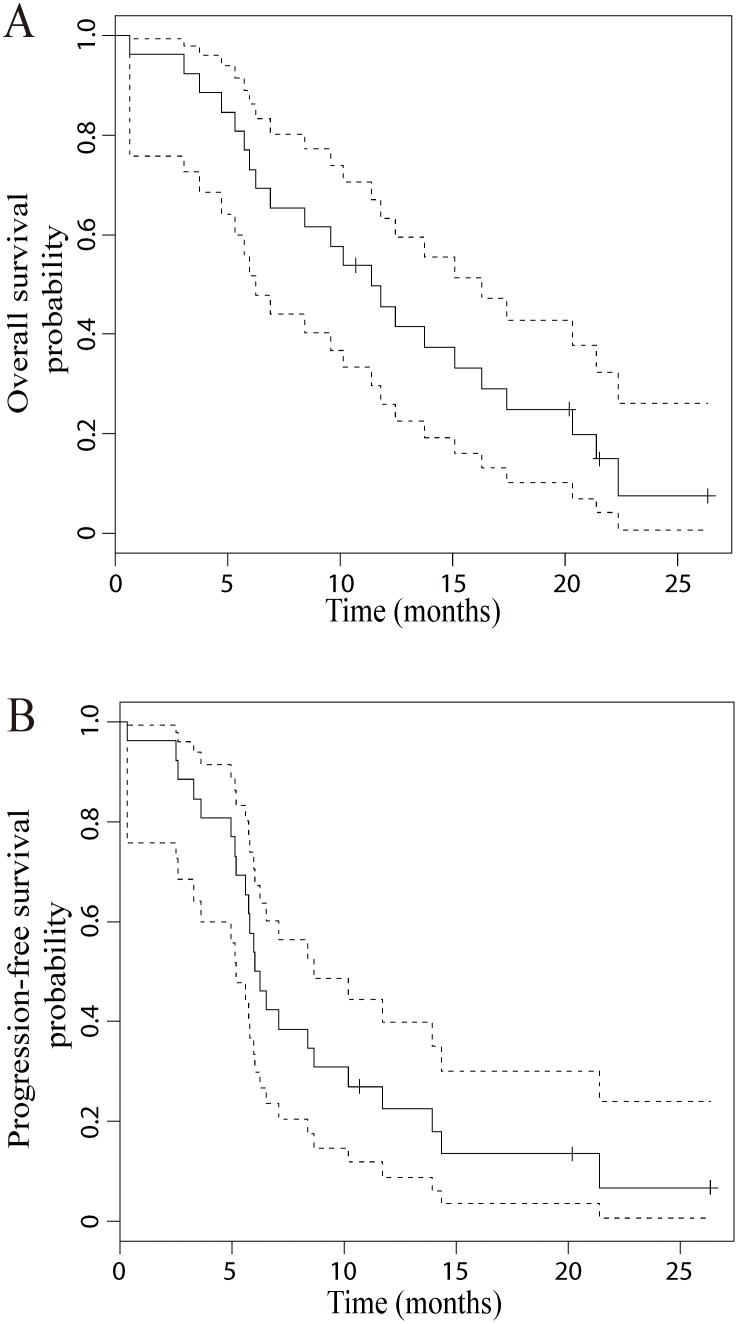
Kaplan-Meier curves showing overall survival (A) and progression free survival (B) with 95% confidence intervals (CI) in the patients enrolled.

**Figure 3:**
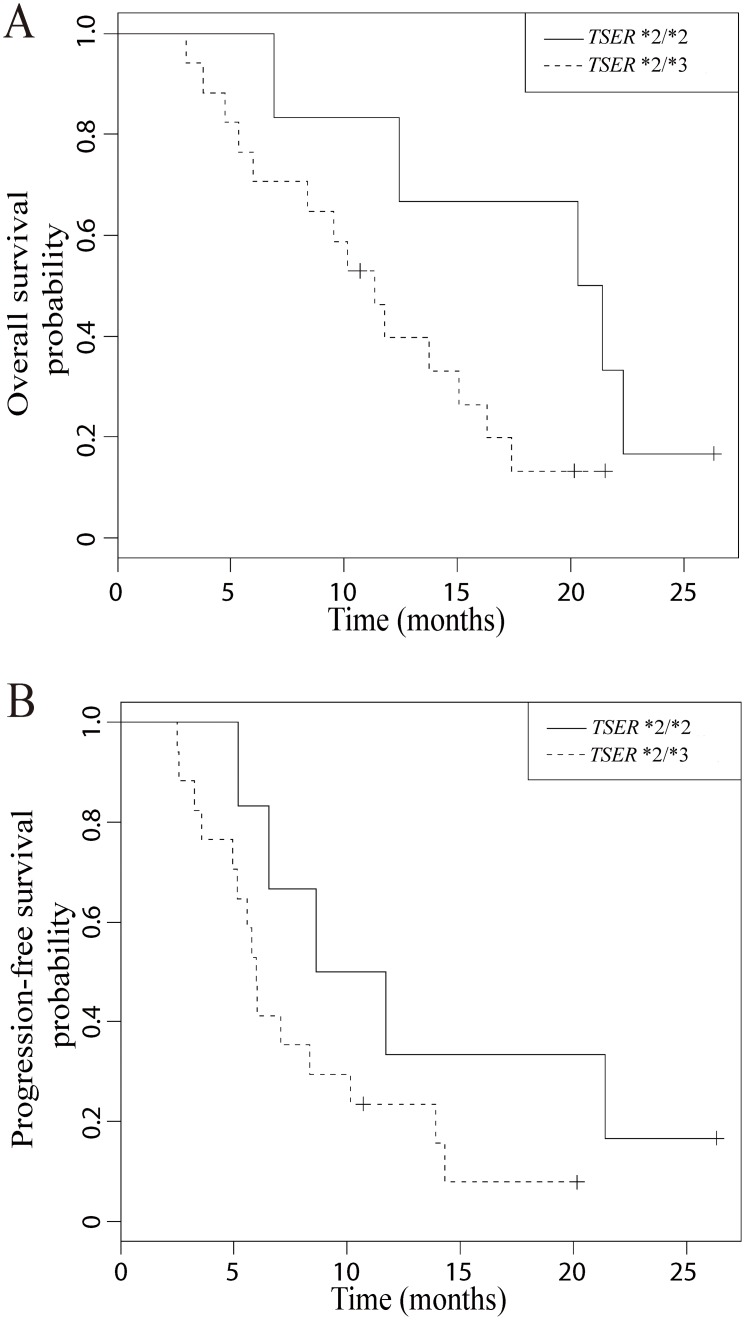
Kaplan-Meier curves showing overall survival (A) and progression free survival (B) according to *TSER* genotypes.

Response to the modified FOLFOX-6 regimen in the 23 evaluable patients is shown in Table 3. Nine patients experienced PR and no patient experienced complete response (CR), making the overall response rate (CR+PR) to be 39.1% (95% confidence interval (CI, Wilson), 22.2 to 59.2). When tumor responses are compared between the two *TSER* genotype groups, patients with the TSER*2/*2 genotype experienced a greater frequency of PR and a less frequency of SD compared to those with the TSER*2/*3 genotype (p = 0.02, Fisher’s exact test, Table 3). On the other hand, only one out of 23 patients experienced PD, for an observed disease control rate (CR+PR+SD) of 95.7% (95% CI, 79.0 to 99.2). OS and PFS plots for the enrolled patients are shown in Figure 2. Median values for OS and PFS were 11.4 months (95% CI, 6.3 to 16.3) and 6.2 months (95% CI, 5.2 to 8.6), respectively. For both OS and PFS, patients with the TSER*2/*2 genotype had a trend towards an improvement in median OS and PFS in comparison to those patients with the TSER*2/*3 genotype (Figure 3, OS, 20.9 vs 11.4 months; PFS, 10.2 vs 6.0 months).

**Table 3:** Tumor responses to the FOLFOX regimen in patients of differing *TSER* genotypes.

Tumor response	*TSER* genotypes				*P-*value^a^
	TSER*2/*2		TSER*2/*3		
	(n = 6)		*(n = 17)*		
	No	%	No	%	
PD (n = 1)	0	0	1	5.9	0.02
PR (n = 9)	5	83.3	4	23.5	
SD (n = 13)	1	16.7	12	70.6	

PD, progressive disease; PR, partial response; SD, stable disease.

a Calculated using Fisher’s exact test for the association between response (PR and SD) and *TSER* genotype.

### Potential associations between tumor response and retrospectively analyzed genetic variations

For 21 patients, the retrospective analyses for additional genotypes reported to be associated with treatment responses were completed (the genotypic and allelic frequencies of the interrogated variations are summarized in Table S2 in File S1). Of note, the frequency of TSER*2 allele is higher in our current study than those reported in the literature since the patients with the TSER*3/*3 genotype were excluded in this study. When the two additional loci in the *TYMS* genes (rs34743033 and rs34489327) were assessed for their potential association with tumor response (PR vs SD), the results did not indicate any statistically significant association (Table 4). Among the five additional genotypes analyzed, two genetic variations in the *XRCC1* (c.1196G>A, rs25487) and *MDR1* (c.3435C>T, rs1045642) genes displayed a trend supportive of their potential association with tumor response (p = 0.050 and 0.056, respectively, Fisher’s exact test, Table 4). However, no apparent association was observed when OS and PFS times were compared among the groups with differing genotypes at the five loci (Table S3 in File S1).

**Table 4:** Univariate association between additional retrospectively analyzed genotypes and tumor response (PR: partial response and SD: stable disease).

Gene	Genotype	Tumor response				*P-*value^a^
		PR (N = 9)		SD (N = 11)		
		No.	%	No.	%	
*TYMS*	5′-UTR *TSER* + G>C (rs34743033)					
	TSER*2/*2	5	56	1	9	
	TSER*2/*3(G)	2	22	4	36	
	TSER*2/*3(C)	2	22	6	55	0.137
*TYMS*	3′-UTR 1494delTTAAAG(6 bp) (rs34489327)					
	+6 bp/+6 bp	5	56	6	55	
	+6 bp/−6 bp	4	44	5	45	1
*ERCC1*	c.354C>T (rs11615)					
	C/C	2	22	1	9	
	C/T	4	45	8	73	
	T/T	3	33	2	18	0.552
*ERCC2*	c.2251A>C (rs13181)					
	A/A	5	56	3	27	
	A/C	3	33	6	55	
	C/C	1	11	2	18	0.485
*GSTP1*	c.313A>G (rs1695)					
	A/A	4	45	8	73	
	A/G	3	33	2	18	
	G/G	2	22	1	9	0.552
*XRCC1*	c.1196G>A (rs25487)					
	A/A	1	11	6	55	
	G/A	6	67	5	45	
	G/G	2	22	0	-	0.050
*MDR1*	c.3435C>T (rs1045642)					
	C/C	0	-	1	9	
	C/T	9	100	6	55	
	T/T	0	-	4	36	0.056

a Calculated using Fisher’s exact test.

## Discussion

This clinical study is unique in that we for the first time applied the strategy of prospectively assessing *TSER* genotypes to improve clinical outcomes in gastric and GEJ cancers. Our initial hypothesis was that selection of patients with good risk *TSER* genotypes would improve the response rate for FOLFOX to 60%, a 17% increase over the historic control response rate of 43% observed in non-genotype selected patients. The observed response rate in this study was 39.1% (9 PR out of 23 patients evaluable for tumor response, 95% CI 22.2 to 59.2), not achieving the primary study endpoint. The median OS and PFS times of 11.3 and 6.2 months in patients with good risk *TSER* genotypes were also comparable to those reported in non-genotype selected populations (Figure 2). Although the observed response rate did not support the utility of *TSER* genotyping as a treatment selection guide, it should also be noted that the enrolled patients experienced a very promising disease control rate of 95.7% (9 PR and 13 SD out of 23 patients), higher than those reported in the literature [32]–[36]. The high disease control rate in the current study may provide the first prospectively obtained evidence for the utility of *TSER* genotyping in improving clinical outcomes in patients with gastric and GEJ cancers.

The apparent lack of treatment outcome improvement in patients with good risk *TSER* genotypes may be related to the small number of patients enrolled in the current study, therefore necessitating further validation of this approach in a larger clinical trial. It should be however noted that we observed a very encouraging response rate of 83.3% in patients with the homozygous TSER*2/*2 genotype (5 out of 6 patients experienced PR). This observation was from a very small number of patients, but it suggests that the improvement in clinical outcomes in patients with gastric and GEJ cancers may require selection of patients with two TSER*2 alleles. Alternatively, the current findings may be related to the presence of multiple molecular/genetic factors contributing to chemotherapy response. Strategies involving more than one host and tumoral markers will likely be more successful in guiding therapeutic decision making. Currently we cannot rule out the possibility that *TSER* genotypes may have prognostic value, independent of therapy, in patients with gastric and GEJ cancers as previously reported in patients with colorectal cancer [37], [38]. Consideration of the above-mentioned aspects will be important in the design of future clinical trials which can evaluate multifactorial treatment selection approaches in a larger number of patients.

The findings from another prospective study with selection of patients on the basis of *TSER* genotypes have been recently reported in rectal cancer patients receiving neoadjuvant chemoradiation (n = 135) [21]. In this particular study, patients with good risk *TSER* genotypes (at least one TSER*2 allele) were treated with standard 5-FU-based chemoradiation. Patients with good risk genotypes had higher rates of downstaging and pathologic complete response than reported in unselected populations. Patients with poor risk TSER genotypes (harboring no TSER*2 allele) were treated with irinotecan in addition to 5-FU-based chemoradiation. Patients in the poor risk groups receiving additional therapies also showed higher rates of downstaging and pathologic complete response than those for unselected population. This study [21] however did not address whether patients in the poor risk group would have had poorer clinical outcomes if they had not received additional irinotecan therapy. Nevertheless, these findings certainly support the utility of this single genotype-based strategy in improving the clinical outcomes of rectal cancer patients.

No improvement in the overall response rate from this study echo the inconsistent results observed in previous studies utilizing *TSER* genotyping in treating patients with gastric and esophageal cancers [6], . Less than expected response rates in our current study as well as other previously reported retrospective studies may be explained by the potential effect of oxaliplatin on tumoral TS expression. The clinical synergy of combining 5-FU with oxaliplatin is well documented and is key to the success and frequent use of this combination regimen [42]–[44]. One proposed explanation for this synergy is oxaliplatin-induced TS downregulation by as yet unexplained mechanisms [45], [46]. If oxaliplatin were to cause tumoral TS downregulation or any potential molecular changes influencing tumor response, any benefits from pretreatment *TSER* genotyping may have been obscured in our current study. Interestingly, some *in vitro* studies also indicate that irinotecan may also decrease TS activities and protein levels where irinotecan could overcome 5-FU resistance in tumors [47], [48]. These observations could help to explain the favorable response results in the “poor risk” patients who received irinotecan in the Tan *et al*. study [21]. However, our current study design of a single arm (patients with the favorable *TSER* genotypes only) does not allow us to gather treatment outcome for patients with the unfavorable *TSER* genotypes. Our initial study design was indeed two-armed (favorable vs. unfavorable *TSER* genotypes). But, the reviewers in the NIH study section recommended that we revise our study to be single armed for patients with favorable *TSER* genotypes given the patient numbers and other resources available. In future clinical trials, it would be certainly interesting to prospectively compare treatment outcomes of patients with favorable and unfavorable *TSER* genotypes.

Retrospective analyses for two additional *TYMS* genotypes showed no significant association with tumor response (Table 4). For additional non-*TYMS* genes analyzed, *XRCC1* (c.1196G>A, rs25487) and *MDR1* (c.3435C>T, rs1045642) showed a potential association to tumor response (Table 4). We performed these analyses based on the previous findings on the potential association between these genotypes and clinical outcomes in colorectal cancer patients treated with FOLFOX [24], [29], [40]. However, we did not observe any strong association between the tested genotypes and tumor response, except a trend reflective of potential associations for *XRCC1* and *MDR1*. Similar negative results have been reported from retrospective analyses of *TYMS* and non-*TYMS* genotypes in relation to the histopathological tumor responses in patients with gastric and esophageal cancers [39], [41], [49]. Interestingly, the polymorphisms in *XRCC1* and *MDR1* have been associated with clinical outcomes in gastric and colorectal cancer [24], [50]. Further investigation in larger clinical trials is warranted to determine whether our current findings are indicative of varying impact of the tested genotypes in different tumor types.

In summary, our strategy to improve response rates in patients with gastric and GEJ cancers by pretreatment *TSER* genotyping was unsuccessful, but even in the setting of some limitations of our current study the high disease control rate was encouraging. The future applicability of a single polymorphism strategy to guide therapy selection, while feasible and attractive, does not appear to yield results of sufficient impact to be generally applicable. Our approach may aid in selecting patients who require chemotherapy intensification to achieve favorable results by adding oxaliplatin or irinotecan and avoid chemotherapy toxicities in those who may have a favorable outcome with 5-FU alone. The contemporary confluence of our capacity to molecularly target cancers and the advancement of our ability to characterize patients and their cancers using molecular genetics will allow for multiple variables to be considered and applied towards improvements in cancer care. *TYMS* polymorphism status does not appear to be a singularly important treatment selection factor, but it may be a key contributor to favorable outcomes in a multivariable genomically based therapeutic approach.

## Supporting Information

File S1 **Supporting Tables.**
(DOCX)

Checklist S1 **TREND Checklist.**
(PDF)

Protocol S1 **Trial Protocol.**
(PDF)

## References

[1] Brown LM, Devesa SS (2002) Epidemiologic trends in esophageal and gastric cancer in the United States. Surg Oncol Clin N Am 11: 235–256. 12424848 10.1016/s1055-3207(02)00002-9

[2] Polednak AP (2003) Trends in survival for both histologic types of esophageal cancer in US surveillance, epidemiology and end results areas. Int J Cancer 105: 98–100. 12672037 10.1002/ijc.11029

[3] Cunningham D, Starling N, Rao S, Iveson T, Nicolson M, et al (2008) Capecitabine and oxaliplatin for advanced esophagogastric cancer. N Engl J Med 358: 36–46. 18172173 10.1056/NEJMoa073149

[4] Bang YJ, Van Cutsem E, Feyereislova A, Chung HC, Shen L, et al (2010) Trastuzumab in combination with chemotherapy versus chemotherapy alone for treatment of HER2-positive advanced gastric or gastro-oesophageal junction cancer (ToGA): a phase 3, open-label, randomised controlled trial. Lancet 376: 687–697. 20728210 10.1016/S0140-6736(10)61121-X

[5] Van Cutsem E, Moiseyenko VM, Tjulandin S, Majlis A, Constenla M, et al (2006) Phase III study of docetaxel and cisplatin plus fluorouracil compared with cisplatin and fluorouracil as first-line therapy for advanced gastric cancer: a report of the V325 Study Group. J Clin Oncol 24: 4991–4997. 17075117 10.1200/JCO.2006.06.8429

[6] Keam B, Im SA, Han SW, Ham HS, Kim MA, et al (2008) Modified FOLFOX-6 chemotherapy in advanced gastric cancer: Results of phase II study and comprehensive analysis of polymorphisms as a predictive and prognostic marker. BMC Cancer 8: 148. 18505590 10.1186/1471-2407-8-148 PMC2442115

[7] Oh SY, Kwon HC, Seo BG, Kim SH, Kim JS, et al (2007) A phase II study of oxaliplatin with low dose leucovorin and bolus and continuous infusion 5-fluorouracil (modified FOLFOX-4) as first line therapy for patients with advanced gastric cancer. Acta Oncol 46: 336–341. 17450469 10.1080/02841860600791483

[8] De Vita F, Orditura M, Matano E, Bianco R, Carlomagno C, et al (2005) A phase II study of biweekly oxaliplatin plus infusional 5-fluorouracil and folinic acid (FOLFOX-4) as first-line treatment of advanced gastric cancer patients. Br J Cancer 92: 1644–1649. 15856038 10.1038/sj.bjc.6602573 PMC2362040

[9] Chu E, Moto AC, Fogarasi MC (2001) Cancer: Principles & Practice of Oncology; De Vita JVT, Hellman S, Rosenberg SA, editors. Philadelphia: Lippincott Williams & Wilkins. 388–415 p.

[10] Marsh S, McLeod HL (2001) Thymidylate synthase pharmacogenetics in colorectal cancer. [see comment]. Clin Colorectal Cancer 1: 175–178 discussion 179–181. 12450432 10.3816/CCC.2001.n.018

[11] Horie N, Aiba H, Oguro K, Hojo H, Takeishi K (1995) Functional analysis and DNA polymorphism of the tandemly repeated sequences in the 5′-terminal regulatory region of the human gene for thymidylate synthase. Cell Struct Funct 20: 191–197. 7586009 10.1247/csf.20.191

[12] Kawakami K, Omura K, Kanehira E, Watanabe Y (1999) Polymorphic tandem repeats in the thymidylate synthase gene is associated with its protein expression in human gastrointestinal cancers. Anticancer Res 19: 3249–3252. 10652619

[13] Ulrich CM, Bigler J, Velicer CM, Greene EA, Farin FM, et al (2000) Searching expressed sequence tag databases: discovery and confirmation of a common polymorphism in the thymidylate synthase gene. Cancer Epidemiol Biomarkers Prev 9: 1381–1385. 11142426

[14] Kawakami K, Salonga D, Park JM, Danenberg KD, Uetake H, et al (2001) Different lengths of a polymorphic repeat sequence in the thymidylate synthase gene affect translational efficiency but not its gene expression. Clin Cancer Res 7: 4096–4101. 11751507

[15] Marsh S, Collie-Duguid ES, Li T, Liu X, McLeod HL (1999) Ethnic variation in the thymidylate synthase enhancer region polymorphism among Caucasian and Asian populations. Genomics 58: 310–312. 10373329 10.1006/geno.1999.5833

[16] Iacopetta B, Grieu F, Joseph D, Elsaleh H (2001) A polymorphism in the enhancer region of the thymidylate synthase promoter influences the survival of colorectal cancer patients treated with 5-fluorouracil. Br J Cancer 85: 827–830. 11556832 10.1054/bjoc.2001.2007 PMC2375084

[17] Pullarkat ST, Stoehlmacher J, Ghaderi V, Xiong YP, Ingles SA, et al (2001) Thymidylate synthase gene polymorphism determines response and toxicity of 5-FU chemotherapy. Pharmacogenomics J 1: 65–70. 11913730 10.1038/sj.tpj.6500012

[18] Villafranca E, Okruzhnov Y, Dominguez MA, Garcia-Foncillas J, Azinovic I, et al (2001) Polymorphisms of the repeated sequences in the enhancer region of the thymidylate synthase gene promoter may predict downstaging after preoperative chemoradiation in rectal cancer. J Clin Oncol 19: 1779–1786. 11251009 10.1200/JCO.2001.19.6.1779

[19] Metzger R, Leichman CG, Danenberg KD, Danenberg PV, Lenz HJ, et al (1998) ERCC1 mRNA levels complement thymidylate synthase mRNA levels in predicting response and survival for gastric cancer patients receiving combination cisplatin and fluorouracil chemotherapy. J Clin Oncol 16: 309–316. 9440758 10.1200/JCO.1998.16.1.309

[20] Yeh KH, Shun CT, Chen CL, Lin JT, Lee WJ, et al (1998) High expression of thymidylate synthase is associated with the drug resistance of gastric carcinoma to high dose 5-fluorouracil-based systemic chemotherapy. Cancer 82: 1626–1631. 9576280 10.1002/(sici)1097-0142(19980501)82:9<1626::aid-cncr5>3.0.co;2-8

[21] Tan BR, Thomas F, Myerson RJ, Zehnbauer B, Trinkaus K, et al (2011) Thymidylate synthase genotype-directed neoadjuvant chemoradiation for patients with rectal adenocarcinoma. J Clin Oncol 29: 875–883. 21205745 10.1200/JCO.2010.32.3212 PMC3068061

[22] Henriquez-Hernandez LA, Murias-Rosales A, Hernandez Gonzalez A, Cabrera De Leon A, Diaz-Chico BN, et al (2009) Gene polymorphisms in TYMS, MTHFR, p53 and MDR1 as risk factors for breast cancer: a case-control study. Oncol Rep 22: 1425–1433. 19885596 10.3892/or_00000584

[23] Lincz LF, Scorgie FE, Garg MB, Ackland SP (2007) Identification of a novel single nucleotide polymorphism in the first tandem repeat sequence of the thymidylate synthase 2R allele. Int J Cancer 120: 1930–1934. 17278107 10.1002/ijc.22568

[24] Huang MY, Huang ML, Chen MJ, Lu CY, Chen CF, et al (2011) Multiple genetic polymorphisms in the prediction of clinical outcome of metastatic colorectal cancer patients treated with first-line FOLFOX-4 chemotherapy. Pharmacogenet Genomics 21: 18–25. 21057378 10.1097/FPC.0b013e3283415124

[25] Huang MY, Fang WY, Lee SC, Cheng TL, Wang JY, et al (2008) ERCC2 2251A>C genetic polymorphism was highly correlated with early relapse in high-risk stage II and stage III colorectal cancer patients: a preliminary study. BMC Cancer 8: 50. 18267032 10.1186/1471-2407-8-50 PMC2262891

[26] Zhang XH, Zhang X, Zhang L, Chen Q, Yang Z, et al (2012) XRCC1 Arg399Gln was associated with repair capacity for DNA damage induced by occupational chromium exposure. BMC Res Notes 5: 263. 22642904 10.1186/1756-0500-5-263 PMC3500259

[27] Lunn RM, Langlois RG, Hsieh LL, Thompson CL, Bell DA (1999) XRCC1 polymorphisms: effects on aflatoxin B1-DNA adducts and glycophorin A variant frequency. Cancer Res 59: 2557–2561. 10363972

[28] Zhang Z, Wan J, Jin X, Jin T, Shen H, et al (2005) Genetic polymorphisms in XRCC1, APE1, ADPRT, XRCC2, and XRCC3 and risk of chronic benzene poisoning in a Chinese occupational population. Cancer Epidemiol Biomarkers Prev 14: 2614–2619. 16284386 10.1158/1055-9965.EPI-05-0143

[29] Mandola MV, Stoehlmacher J, Muller-Weeks S, Cesarone G, Yu MC, et al (2003) A novel single nucleotide polymorphism within the 5′ tandem repeat polymorphism of the thymidylate synthase gene abolishes USF-1 binding and alters transcriptional activity. Cancer Res 63: 2898–2904. 12782596

[30] Kawakami K, Watanabe G (2003) Identification and functional analysis of single nucleotide polymorphism in the tandem repeat sequence of thymidylate synthase gene. Cancer Res 63: 6004–6007. 14522928

[31] Simon R (1989) Optimal two-stage designs for phase II clinical trials. Control Clin Trials 10: 1–10. 2702835 10.1016/0197-2456(89)90015-9

[32] Ocvirk J, Rebersek M, Skof E, Hlebanja Z, Boc M (2012) Randomized prospective phase II study to compare the combination chemotherapy regimen epirubicin, cisplatin, and 5-fluorouracil with epirubicin, cisplatin, and capecitabine in patients with advanced or metastatic gastric cancer. Am J Clin Oncol 35: 237–241. 21399488 10.1097/COC.0b013e31820dc0b0

[33] Moon YW, Rha SY, Jeung HC, Kim C, Hong MH, et al (2010) Outcomes of multiple salvage chemotherapy for advanced gastric cancer: implications for clinical practice and trial design. Cancer Chemother Pharmacol 66: 797–805. 20221831 10.1007/s00280-010-1295-z

[34] Moehler M, Mueller A, Trarbach T, Lordick F, Seufferlein T, et al (2011) Cetuximab with irinotecan, folinic acid and 5-fluorouracil as first-line treatment in advanced gastroesophageal cancer: a prospective multi-center biomarker-oriented phase II study. Ann Oncol 22: 1358–1366. 21119032 10.1093/annonc/mdq591

[35] Koizumi W, Takiuchi H, Yamada Y, Boku N, Fuse N, et al (2010) Phase II study of oxaliplatin plus S-1 as first-line treatment for advanced gastric cancer (G-SOX study). Ann Oncol 21: 1001–1005. 19875759 10.1093/annonc/mdp464

[36] Amarantidis K, Xenidis N, Chelis L, Chamalidou E, Dimopoulos P, et al (2011) Docetaxel plus oxaliplatin in combination with capecitabine as first-line treatment for advanced gastric cancer. Oncology 80: 359–365. 21811088 10.1159/000330199

[37] Edler D, Hallstrom M, Johnston PG, Magnusson I, Ragnhammar P, et al (2000) Thymidylate synthase expression: an independent prognostic factor for local recurrence, distant metastasis, disease-free and overall survival in rectal cancer. Clin Cancer Res 6: 1378–1384. 10778966

[38] Popat S, Matakidou A, Houlston RS (2004) Thymidylate synthase expression and prognosis in colorectal cancer: a systematic review and meta-analysis. J Clin Oncol 22: 529–536. 14752076 10.1200/JCO.2004.05.064

[39] Ott K, Vogelsang H, Marton N, Becker K, Lordick F, et al (2006) The thymidylate synthase tandem repeat promoter polymorphism: A predictor for tumor-related survival in neoadjuvant treated locally advanced gastric cancer. Int J Cancer 119: 2885–2894. 16929515 10.1002/ijc.22235

[40] Kawakami K, Graziano F, Watanabe G, Ruzzo A, Santini D, et al (2005) Prognostic role of thymidylate synthase polymorphisms in gastric cancer patients treated with surgery and adjuvant chemotherapy. Clin Cancer Res 11: 3778–3783. 15897576 10.1158/1078-0432.CCR-04-2428

[41] Okuno T, Tamura T, Yamamori M, Chayahara N, Yamada T, et al (2007) Favorable genetic polymorphisms predictive of clinical outcome of chemoradiotherapy for stage II/III esophageal squamous cell carcinoma in Japanese. Am J Clin Oncol 30: 252–257. 17551301 10.1097/01.coc.0000256059.88247.25

[42] Lordick F, Lorenzen S, Stollfuss J, Vehling-Kaiser U, Kullmann F, et al (2005) Phase II study of weekly oxaliplatin plus infusional fluorouracil and folinic acid (FUFOX regimen) as first-line treatment in metastatic gastric cancer. Br J Cancer 93: 190–194. 16012522 10.1038/sj.bjc.6602697 PMC2361546

[43] Chao Y, Yeh KH, Chang CJ, Chen LT, Chao TY, et al (2004) Phase II study of weekly oxaliplatin and 24-h infusion of high-dose 5-fluorouracil and folinic acid in the treatment of advanced gastric cancer. Br J Cancer 91: 453–458. 15226770 10.1038/sj.bjc.6601985 PMC2409850

[44] Rothenberg ML, Oza AM, Bigelow RH, Berlin JD, Marshall JL, et al (2003) Superiority of oxaliplatin and fluorouracil-leucovorin compared with either therapy alone in patients with progressive colorectal cancer after irinotecan and fluorouracil-leucovorin: interim results of a phase III trial. J Clin Oncol 21: 2059–2069. 12775730 10.1200/JCO.2003.11.126

[45] Yeh KH, Cheng AL, Wan JP, Lin CS, Liu CC (2004) Down-regulation of thymidylate synthase expression and its steady-state mRNA by oxaliplatin in colon cancer cells. Anticancer Drugs 15: 371–376. 15057142 10.1097/00001813-200404000-00010

[46] Chen CC, Chen LT, Tsou TC, Pan WY, Kuo CC, et al (2007) Combined modalities of resistance in an oxaliplatin-resistant human gastric cancer cell line with enhanced sensitivity to 5-fluorouracil. Br J Cancer 97: 334–344. 17609664 10.1038/sj.bjc.6603866 PMC2360324

[47] Fukushima M, Sakamoto K, Ohshimo H, Nakagawa F, Taguchi T (2010) Irinotecan overcomes the resistance to 5-fluorouracil in human colon cancer xenografts by down-regulation of intratumoral thymidylate synthase. Oncol Rep 24: 835–842. 20811661 10.3892/or.2010.835

[48] Torigoe S, Ogata Y, Matono K, Shirouzu K (2009) Molecular mechanisms of sequence-dependent antitumor effects of SN-38 and 5-fluorouracil combination therapy against colon cancer cells. Anticancer Res 29: 2083–2089. 19528468

[49] Toriumi F, Kubota T, Saikawa Y, Yoshida M, Otani Y, et al (2004) Thymidylate synthetase (TS) genotype and TS/dihydropyrimidine dehydrogenase mRNA level as an indicator in determining chemosensitivity to 5-fluorouracil in advanced gastric carcinoma. Anticancer Res 24: 2455–2463. 15330198

[50] Li Y, Yan PW, Huang XE, Li CG (2011) MDR1 gene C3435 T polymorphism is associated with clinical outcomes in gastric cancer patients treated with postoperative adjuvant chemotherapy. Asian Pac J Cancer Prev 12: 2405–2409. 22296392

