# Supplementary material for: Thymidylate Synthase Genotype-Directed Chemotherapy for Patients with Gastric and Gastroesophageal Junction Cancers
Source: PLoS One. 2014 Sep 18;9(9):e107424. doi: 10.1371/journal.pone.0107424 (PMC4169411; doi:10.1371/journal.pone.0107424)
Supplement: File S1 — Supporting Tables. (DOCX) [file pone.0107424.s001.docx]

**SUPPLEMENTARY DATA**

**Table S1** Summary of experimental conditions used for PCR-based restriction fragment length polymorphism (RFLP) genotyping assays according to the published procedures [[13](#_ENREF_13),[22-30](#_ENREF_22)]

| **Gene (SNP)** | **Primer sequences** | **PCR Condition (40 cycles)** |  | **Product size (bp)** | **Genotype interpretation after digest** |  |  |  |
| --- | --- | --- | --- | --- | --- | --- | --- | --- |
| *TYMS* 5’UTR +G/C | F:5'GTGGCTCCTGCGTTTCCCCC-3, R:5'GCTCCGAGCCGGCCACAGGCATGGCGCGG-3' | 94⁰C, 30 sec 63⁰C, 1 min 72⁰C, 1 min |  | 210 (2R) 238 (3R) | *Hae*III (2RGC): 66, 47 bp (3RG): 66, 47 bp (3RC): 94, 66, 47 bp |  |  |  |
| *TYMS* 3′-UTR 149del6 | F:5'CAAATCTGAGGGAGCTGAGT-3, R:5'CAGATAAGTGGCAGTACAGA-3' | 94⁰C, 30 sec 58⁰C, 30 sec 72⁰C, 1 min |  | 152 | *Dra*I (6bp+/+): 70,88 bp (6bp-/-): 152 bp (6bp+/-): 152, 70,80 bp | |  |  |
| *ERCC1* (C354T) | F:5'GCAGAGCTCACCTGAGGAAC-3, R:5'GAGGTGCAAGAAGAGGTGGA-3' | 94⁰C, 30 sec 65⁰C, 1 min 72⁰C, 1 min |  | 208 | *BsrD*I (C): 208 bp  (T): 128, 80 bp | |  |  |
| *ERCC2* (A2251C) | F:5'TCTGCAGGAGGATCAGCTG-3, R:5'GCAAGACTCAGGAGTCAC-3' | 94⁰C, 30 sec 64⁰C, 1 min 72⁰C, 1 min |  | 149 | *Pst*I (A): 143, 6 bp (C): 80, 63, 6 bp | | |  |
| *GSTP1* (A313G) | F:5'GTAGTTTGCCCAAGGTCAAG-3, R:5'AGCCACCTGAGGGGTAAG-3' | 94⁰C, 30 sec 65⁰C, 1 min 72⁰C, 1 min |  | 433 | *BsmA*I (A): 329, 104 bp (G): 222, 107, 104 bp | | |  |
| *XRCC1* (G1196A) | F:5'TTGTGCTTTCTCTGTGTCCA-3, R:5'TCCTCCAGCCTTTTCTGATA-3' | 94⁰C, 30 sec 54⁰C, 1 min 72⁰C, 1 min |  | 615 | *Msp*I (G): 615 bp (A): 375, 240 bp | | |  |
| *MDR1* (C3435T) | F:5'GATCTGTGAACTCTTGTTTTC-3, R:5'GAAGAGAGACTTACATTAGGC-3' | 94⁰C, 30 sec 58⁰C, 1 min 72⁰C, 1 min |  | 244 | *Dpn*II (C): 172, 72 bp (T): 244 bp | | |  |

**Table S2** Genotypic and allelic frequencies of additional genetic polymorphisms retrospectively analyzed in the patients enrolled (n=21)

| **Gene** | **Genotype** | **Frequency Total n= 21^a^** | |  | **Allele** | **Frequency Total N= 42** | |
| --- | --- | --- | --- | --- | --- | --- | --- |
|  |  | **No** | **%** |  |  | **No** | **%** |
| *TYMS* | 5′-UTR *TSER* + G>C ( rs34743033) |  |  |  |  |  |  |
|  | *TSER*2/*2* | 6 | 29 |  | **2* | 27 | 64 |
|  | *TSER*2/*3(G)* | 7 | 33 |  | **3(G)* | 7 | 17 |
|  | *TSER*2/*3(C)* | 8 | 38 |  | **3(C)* | 8 | 19 |
|  |  |  |  |  |  |  |  |
| *TYMS* | 3′-UTR 1494delTTAAAG(6bp) ([rs34489327](http://www.pharmgkb.org/rsid/rs34489327)) |  |  |  |  |  |  |
|  | +6 bp/+6 bp | 11 | 52 |  | +6 bp | 32 | 76 |
|  | +6 bp/-6 bp | 10 | 48 |  | -6 bp | 10 | 24 |
|  |  |  |  |  |  |  |  |
| *ERCC1* | c.354C>T (rs11615) |  |  |  |  |  |  |
|  | C/C | 3 | 14 |  | C | 19 | 45 |
|  | C/T | 13 | 62 |  | T | 23 | 55 |
|  | T/T | 5 | 24 |  |  |  |  |
|  |  |  |  |  |  |  |  |
| *ERCC2* | c.2251A>C (rs13181) |  |  |  |  |  |  |
|  | A/A | 9 | 43 |  | A | 27 | 64 |
|  | A/C | 9 | 43 |  | C | 15 | 36 |
|  | C/C | 3 | 14 |  |  |  |  |
|  |  |  |  |  |  |  |  |
| *GSTP1* | c.313A>G (rs1695) |  |  |  |  |  |  |
|  | A/A | 12 | 57 |  | A | 30 | 71 |
|  | A/G | 6 | 29 |  | G | 12 | 29 |
|  | G/G | 3 | 14 |  |  |  |  |
|  |  |  |  |  |  |  |  |
| *XRCC1* | c.1196G>A (rs25487) |  |  |  |  |  |  |
|  | A/A | 7 | 33 |  | G | 16 | 38 |
|  | G/A | 12 | 57 |  | A | 26 | 62 |
|  | G/G | 2 | 10 |  |  |  |  |
|  |  |  |  |  |  |  |  |
| *MDR1* | c.3435C>T (rs1045642) |  |  |  |  |  |  |
|  | C/C | 1 | 5 |  | C | 18 | 43 |
|  | C/T | 16 | 76 |  | T | 24 | 57 |
|  | T/T | 4 | 19 |  |  |  |  |

^a^ Out of 23 patients treated and evaluated for tumor response, 21 patient samples were available for additional post-treatment genotyping analyses.

**Table S3** Association between additional genotypes and survival represented as median overall survival (OS) and median progression free survival (PFS)

| **Gene** | **Genotype** |  |  | | **Median OS** | | |  |  | **Median PFS** | | |  |
| --- | --- | --- | --- | --- | --- | --- | --- | --- | --- | --- | --- | --- | --- |
|  |  | **N** | | **Months** | | **95% CI** | ***P-*value**^a^ |  | **Months** | | **95% CI** | ***P-*value**^a^ | |
| *ERCC1* | c.354C>T (rs11615) | | | | |  |  |  |  | |  |  | |
|  | C/C | 3 | | 9.6 | | 6.0-NA |  |  | 6.0 | | 6.0-NA |  | |
|  | C/T | 13 | | 13.8 | | 11.4-NA |  |  | 7.0 | | 5.6-NA |  | |
|  | T/T | 5 | | 8.4 | | 5.3-NA | 0.144 |  | 4.9 | | 3.6-NA | 0.320 | |
| *ERCC2* | c.2251A>C (rs13181) | | | | |  |  |  |  | |  |  | |
|  | A/A | 9 | | 9.6 | | 6.0-NA |  |  | 6.0 | | 4.9-NA |  | |
|  | A/C | 9 | | 13.8 | | 11.8-NA |  |  | 7.0 | | 5.8-NA |  | |
|  | C/C | 3 | | 6.9 | | 4.7-NA | 0.580 |  | 5.2 | | 3.3-NA | 0.328 | |
| *GSTP1* | c.313A>G (rs1695) | | | | |  |  |  |  | |  |  | |
|  | A/A | 12 | | 11.8 | | 8.4-NA |  |  | 6.4 | | 5.1-NA |  | |
|  | A/G | 6 | | 16.4 | | 6.9-NA |  |  | 7.6 | | 5.2-NA |  | |
|  | G/G | 3 | | 9.6 | | 6.0-NA | 0.252 |  | 6.0 | | 3.6-NA | 0.450 | |
| *XRCC1* | c.1196G>A (rs25487) | | | | |  |  |  |  | |  |  | |
|  | A/A | 7 | | 10.1 | | 6.0-NA |  |  | 5.8 | | 3.6-NA |  | |
|  | G/A | 12 | | 11.9 | | 6.9-NA |  |  | 6.3 | | 5.2-NA |  | |
|  | G/G | 2 | | 20.3 | | NA | 0.210 |  | 8.6 | | 8.6-NA | 0.153 | |
| *MDR1* | c.3435C>T (rs1045642) | | | | |  |  |  |  | |  |  | |
|  | C/C | 1 | | 11.4 | | NA |  |  | 5.6 | | NA |  | |
|  | C/T | 16 | | 11.0 | | 6.0-NA |  |  | 6.0 | | 4.9-NA |  | |
|  | T/T | 4 | | 15.6 | | 6.9-NA | 0.781 |  | 8.6 | | 5.2-NA | 0.721 | |

^a^ Calculated using Log-rank test
