# Supplementary material for: Thymidylate Synthase Genotype-Directed Chemotherapy for Patients with Gastric and Gastroesophageal Junction Cancers
Source: PLoS One. 2014 Sep 18;9(9):e107424. doi: 10.1371/journal.pone.0107424 (PMC4169411; doi:10.1371/journal.pone.0107424)
Supplement: Protocol S1 — Trial Protocol. (PDF) [file pone.0107424.s003.pdf]

TITLE: *Pharmacogenomically Selected Treatment for Gastric and Gastroesophageal Junction (GEJ) Tumors: A Phase II Study*

Coordinating Center: ***Vanderbilt University Medical Center***

Principal Investigator:** ***Laura Williams Goff, MD
777 Preston Research Bldg.
Nashville, TN 37232-6307
Phone: 615-322-4967
Fax: 615-343-7602
laura.williams.1@vanderbilt.edu

Co-Principal Investigator: ***A. Craig Lockhart, MD***
Washington University Medical Center

Co-Investigators: ***James Posey III, MD***
University of Alabama at Birmingham
Howard McLeod, PhD
University of North Carolina
Edith Mitchell, MD
Thomas Jefferson University

Statistician: ***Yu Shyr, PhD***
Dept. of Biostatistics - Biostatistics Shared Resource at VICC
571 Preston Research Bldg.
Nashville, TN 37232-6848
Phone: 615-936-2572
Fax: 615-936-2602

Responsible Research Nurse:
GI team nurse
VICC-Clinical Trials Shared Resources
491 Preston Research Bldg.
Nashville, TN 37232-6868
Phone: 615-936-5795
Fax: 615-936-5850

Responsible Data Manager:
GI team data manager
VICC-Clinical Trials Shared Resources
491 Preston Research Bldg.
Nashville, TN 37232-6868
Phone: 615-936-5795
Fax: 615-936-5850

NCI Supplied Agent: *None*

Protocol version ~~9~~10: ***~~November 23, 2010~~December 28, 2010***

SCHEMA

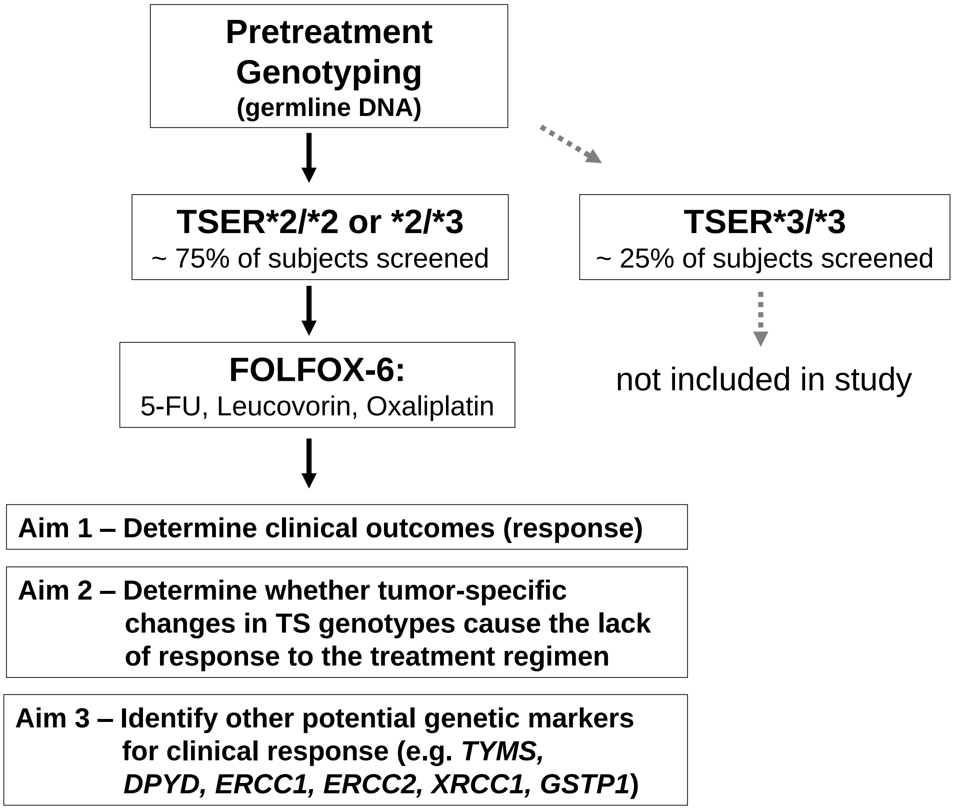
Pretreatment Genotyping
(germline DNA)
TSER*2/*2 or *2/*3
~ 75% of subjects screened
TSER*3/*3
~ 25% of subjects screened
FOLFOX-6:
5-FU, Leucovorin, Oxaliplatin
not included in study
Aim 1 – Determine clinical outcomes (response)
Aim 2 – Determine whether tumor-specific changes in TS genotypes cause the lack of response to the treatment regimen
Aim 3 – Identify other potential genetic markers for clinical response (e.g. TYMS, DPYD, ERCC1, ERCC2, XRCC1, GSTP1)

TABLE OF CONTENTS

	Page
SCHEMA	i
1. OBJECTIVES	1
1.1. Primary Objective	1
1.2. Secondary Objectives	1
2. BACKGROUND	1
2.1. Gastric and GEJ Cancers	1
2.2. Proposed Study Regimens	3
2.3. Pharmacogenomics of the Chemotherapy Agents in the Proposed Study	4
2.4. Rationale	8
3. PATIENT SELECTION	8
3.1. Eligibility Criteria	8
3.2. Exclusion Criteria	9
3.3. Inclusion of Women and Minorities	10
3.4. Research Eligibility Evaluation	11
4. TREATMENT PLAN	11
4.1. Study Design	11
4.2. Study Procedures	12
4.3. Supportive Care Guidelines	12
4.4. Duration of Therapy	13
5. DOSING DELAYS/DOSE MODIFICATIONS	14
5.1. Dose Modifications	14
6. PHARMACEUTICAL INFORMATION	15
6.1. Oxaliplatin	15
6.2. Fluorouracil	18
6.3. Leucovorin Calcium	19
7. RATIONALE FOR SECONDARY OBJECTIVES	20
7.1. Loss of Heterozygosity (LOH) in Tumor Tissues	21
7.2. Tumor TS mRNA and Protein Expression	21
7.3. Assessment of Polymorphisms in Other Genes Associated with Treatment Outcomes of Toxicity	21
8. STUDY CALENDAR	22
9. MEASUREMENT OF EFFECT	23
9.1. Definitions	23
9.2. Guidelines for Evaluation of Measurable Disease	24
9.3. Response Criteria	25
9.4. Confirmatory Measurement/Duration of Response	26
9.5. Progression-Free Survival	27
9.6. Response Review	27

10. DATA SAFETY AND MONITORING PLAN 27
10.1. Institutional Monitoring and Data Management 27
10.2. Confidentiality 30
10.3. Oncore Database 30
10.4. Data Sharing Plan 31

11. STATISTICAL CONSIDERATIONS 33
11.1. Study Design/Endpoints 33
11.2. Sample Size 33
11.3. Patient Accrual 34
11.4. Patient Selection 34
11.5. Analysis of Secondary Endpoints 34
11.6. Reporting and Exclusions 36

REFERENCES 37

INFORMED CONSENT TEMPLATE 43

APPENDICES

APPENDIX A
Performance Status Criteria A-1

APPENDIX B
CTCAE v3.0 B-1

APPENDIX C
SAE Reporting Form C-1

1. OBJECTIVES

1.1. Primary Objective

Determine whether the response rate to a 5-FU containing regimen in patients with metastatic gastric and GEJ adenocarcinomas will be increased by genotype-based treatment selection. "Good risk" patients (TSER*2/*2 or *2/*3, low TS expression genotype) will be treated with a standard 5-FU containing regimen. The response rate in these selected patients will be compared to historical control response rates in non-genotype selected patients.

TSER Status

*2/*3 or *2/*2 ⟶ FOLFOX (5-FU, leucovorin, oxaliplatin)
*3/*3 ⟶ Not included in study

1.2. Secondary Objectives

1.2.1. Examine whether tumor-specific changes in *TSER* genotype (loss of heterozygosity) contribute to any lack of response to a 5-FU containing regimen.

1.2.2. Determine whether other genetic polymorphisms (e.g. *TYMS, DPYD, ERCC1, ERCC2, XRCC1* and *GSTP1*) can influence the response/toxicity in the treated patients.

2. BACKGROUND

2.1. Gastric and GEJ Cancers

2.1.1. Incidence, Epidemiology and Prognosis

Gastric cancer ranks 14th in incidence and is the 8th leading cause of cancer mortality among the major cancer types in the United States, with an estimated 22,000 new cases and 11,500 deaths from this disease in 2005 [1]. Despite knowledge about several risk factors for gastric cancer, the precise etiology remains unknown [13]. Interestingly, the sites of cancer origin within the stomach have changed in frequency in the United States over recent decades [31]. Distal gastric tumors have been decreasing in frequency in the United States since the 1930s; conversely the incidence of cancer of the cardia and gastroesophageal junction (GEJ) has been rapidly rising, five- to six-fold in the past few decades, especially in patients younger than 40 years of age. In fact when adenocarcinomas of distal esophagus are included, gastric cardia and GEJ adenocarcinomas are increasing in frequency more rapidly than any other malignancy [8].

The prognosis for the vast majority of patients with gastric and GEJ adenocarcinoma is very poor as 80-90% of the patients diagnosed in the US present with metastatic disease in either regional or distant sites [48, 65]. The overall survival rate in patients with disseminated disease at 5 years is essentially zero since most tumors develop rapid drug resistance and the disease progresses within months. Despite the poor prognosis, palliative chemotherapy has a proven survival advantage over best supportive care in patients with gastric and GEJ cancer. The survival advantage has been demonstrated in randomized trials where patients assigned to receive best supportive care alone fared significantly worse than those assigned to receive chemotherapy [18, 47, 55].

2.1.2. Stagnation in Clinical Outcomes

Since the demonstration of a survival benefit for palliative chemotherapy in patients with metastatic gastric and GEJ cancer, a number of newer chemotherapies with proven anti-tumor activity in this disease, most notably cisplatin, have been evaluated. This has provided some optimism that the newer treatment regimens would further increase the survival benefit for chemotherapy treatment. The original randomized studies demonstrated an overall tumor response rate of 20-50% and a median survival of 9 to 11 months. Despite the incorporation of newer chemotherapy agents, as single agents or in combination regimens, into gastric and GEJ cancer clinical trials, the response rates and median survival for treated patients has not significantly improved (Table 1). Therefore new therapeutic approaches to improve upon the state-of-the-art treatment of gastric and GEJ cancers are needed. Table 1 presents the results of recent clinical trials of many of the current first-line chemotherapy regimens for advanced/metastatic gastric and GEJ cancers that would be considered "rational" based on available clinical data.

TABLE 1: Results from recent clinical trials in gastric and GEJ cancers demonstrating overlapping response rates and median survival.

Regimen	Response (%)	Median Survival (months)	Author
Epirubicin/cisplatin/5-FU	42 – 46	8.7 - 9.4	Ross; Webb [59, 80]
Docetaxel/cisplatin/5-FU	38.7	10.2	Ajani [3]
Docetaxel/cisplatin	37 – 56	9.0 – 10.4	Roth; Ridwelski [58, 60]
Paclitaxel/cisplatin /5-FU infusion/LV	51	6.5 – 14.0	Kollmannsberger; Kim [33, 34]
Irinotecan/cisplatin	33 – 58	9.0 – 10.5	Boku; Ajani; Lim; Pozzo [2, 9, 36, 53]
5-FU/cisplatin	23 – 34	7.3 – 8.5	Ohtsu; Ajani [3, 49]
Irinotecan/FU/LV	40 (28–54)	N/A	Dank [15]
FOLFOX	26 - 45	7.3 – 11.2	Kim; Louvet; Al-Batran; De Vita [4, 16, 32, 37]
IROX	50	8.5	Souglakos [67]

In December of 2002 the National Cancer Institute convened a Progress Review Group (PRG) for stomach and esophageal cancers. The priority recommendations from the PRG covered multiple areas including therapy and therapeutic targets. Of note, it was the conclusion of the PRG that there was a need to: "Develop and test novel therapeutics, and optimize existing treatments for gastroesophageal cancers and their precursors, based on the identification and understanding of molecular pathways involved in oncogenesis, tumor response and resistance." And to: "Define host and molecular/biologic tumor characteristics that will help customize treatment and best predict recurrence and/or survival" (http://prg.nci.nih.gov/stomach/stomach_esophageal.pdf). **Pharmacogenomically based selection of chemotherapy for the treatment of gastric and GEJ cancer may allow physicians to more accurately select effective treatment for patients and consequently improve upon the current response rates associated with chemotherapy for this disease.**

2.1.3. Current State-of-the-Art Treatment for Advanced Gastric and GEJ Cancers

Multiple chemotherapeutic agents have been previously shown to have antitumor activity in gastric and GEJ cancers (Reviewed in [26, 61]). Active chemotherapy agents that have been extensively studied in combinations and as single agents include fluorouracil, cisplatin, the anthracyclines doxorubicin and epirubicin, mitomycin C, and etoposide. More recently "newer" chemotherapy agents such as paclitaxel, docetaxel, irinotecan and oxaliplatin have been incorporated into the treatment regimens although in some cases the precise role that some of these agents may eventually play has not been fully determined (Table 1). Nevertheless there has been no significant improvement in the overall response rate and median survival for this group of patients and there is no generally accepted standard treatment regimen for these patients. Interpretation of the available data from Phase III trials in gastric and GEJ cancer treatment has been difficult because minor improvements in efficacy have been associated with significant toxicity. Therefore, the selection of a treatment regimen for a particular patient has been largely based on the physician's prior experience and comfort with administering a particular regimen, patient preferences regarding side-effect profiles, co-morbid conditions that could affect chemotherapy tolerance, marketing influences or reimbursement. **Treatment selection based on host or tumor genetic characteristics is a rational and potentially more powerful means of treatment selection.**

2.2. Proposed Study Regimen: FOLFOX-6

The combination regimen proposed in this application is modified FOLFOX-6 (5-FU, leucovorin, oxaliplatin). This regimen has extensive safety data from large trials in colon cancer and has been previously shown to have a response rate and median survival comparable to other regimens used in gastric and GEJ cancer (Response rate=43%; Median Survival=9.6 months) [4, 16, 32, 37].

In first-line studies in gastric cancer, the dose of oxaliplatin has varied from 85 to 100 mg/m^2 and the continuous infusion of 5-FU has been administered over 22 hours on 2 days (standard FOLFOX-4) or over 46 hours (modified FOLFOX). Response rates in this treatment setting have ranged from 38 – 44.9% and median survival duration has ranged from 8.6 – 11.2 months which are comparable with those of other oxaliplatin-based regimens, suggesting a role for this combination in gastric cancer [4, 16, 37].

The combination of 5-FU, leucovorin and oxaliplatin (FOLFOX) has been evaluated extensively in colorectal cancer as well as in other malignancies. The FOLFOX trials have tested this bimonthly regimen with a variety of oxaliplatin doses (85, 100 or 130 mg/m^2) and alterations in the 5-FU infusion schedule. The modified schedules of FOLFOX where patients receive bolus high-dose LV, 5-FU bolus on day 1 only and high-dose 5-FU infused over 46 h with a disposable pump for outpatient therapy have been widely adopted [6, 39, 40, 75]. This regimen is more comfortable for patients, less costly and at least as active, with lower toxicity, than the previous bimonthly regimens in which LV infusion had been repeated for 2 consecutive days.

The regimen FOLFOX selected for this study is the modified FOLFOX-6 regimen where oxaliplatin is administered at 85 mg/m^2 with leucovorin 400 mg/m^2 IV over 120 minutes, bolus 5-FU 400 mg/m^2 IV push, followed by a 46 hour infusion of 5-FU at a dose of 2400 mg/m^2. The regimen was selected based on its very common usage in other malignancies, the ease of administration and excellent tolerability.

2.3. Pharmacogenomics of the Chemotherapy Agents in the Proposed Study

Pharmacogenomics, defined as the use of genetic information to predict the safety, toxicity, and/or efficacy of drugs in individual patients or groups of patients, is a potentially powerful therapeutic approach that aims to elucidate the genetic basis for interindividual differences in drug response. When incorporated into rationally designed clinical trials, pharmacogenomics may improve treatment outcomes. A better understanding of the genetic determinants of chemotherapeutic response will enable prospective identification of patients at risk for treatment failure or those most likely to benefit from a particular treatment regimen. These studies can be translated to clinical practice via molecular diagnostics (genotyping) in order to guide selection of the optimal drug combination and dosage for the individual patient. Until recently this approach has largely focused on genetic variations associated with drug toxicity, such as variations in the *TPMT* gene and toxicity associated with 6-mercaptopurine (6-MP) when used in the treatment of leukemia and variations in the *UGT1A1* gene associated with irinotecan treatment related toxicity [5, 17, 25]. This proposal is focused on the prospective incorporation of patient genetic information to predict treatment outcome. **This is the first known prospective clinical trial in patients with gastric and GEJ tumors where therapy will be determined based upon the patients' genetic information.**

The chemotherapy agents to be used in this proposal (5-FU and oxaliplatin) have genetic variations associated with treatment outcomes (toxicity and/or efficacy). The most common genetic variations are summarized below (Table 2).

TABLE 2: Summary of genetic variations to be examined in the proposed study

gene name (ID)	Function	Nucleotide	Protein	Allelic frequency (%)	Effects
**5-FU associated**					
TYMS (7298)	DNA synthesis	28 bp tandem repeats (TSER*3)	5'UTR	50-80	increased expression
		G>C (within the 2nd tandem repeat)	5'UTR	15-33	decreased expression
		6 bp deletion	3'UTR	29	decreased expression
DPYD (1806)	5-FU catabolism	IVS14+1 G>A	deletion (exon 14)	1	no expression
**Oxaliplatin associated**					
ERCC1 (2067)	DNA repair	C354T	Asn118Asn	30	decreased cisplatin effect
		C8092A	3'UTR	30	
ERCC2 (2068)	DNA repair	A2251C	Lys751Gln	35-40	decrease or increase in activity?
XRCC1 (7515)	DNA repair	G1196A	Arg399Gln	30	decrease in activity
GSTP1 (2950)	detoxification	A313G	Ile105Val	30	decrease in activity

2.3.1. 5-Fluorouracil (5-FU)

Based on its activity in upper GI tumors and synergistic interactions with other chemotherapy agents, 5-fluorouracil (5-FU) remains one of the most commonly prescribed chemotherapy agents. Most standard regimens for gastric and GEJ tumors include 5-FU. In this proposal, we hypothesize that subjects with a common genotype that is associated with 5-FU resistance will have an improved outcome if treated with a non-5-FU containing active regimen. 5-FU metabolism is briefly summarized as follows: approximately 5% of administered 5-FU undergoes anabolism into cytotoxic nucleotides responsible for its antitumor activity, whereas the other 80–95% undergoes catabolism into biologically inactive metabolites that are excreted in the urine and bile [11]. Dihydropyrimidine dehydrogenase (DPD) is a critical enzyme in 5-FU catabolism. Thymidylate synthase (TS) is a primary target for 5-FU antitumor activity. We plan to examine the polymorphisms in the genes

encoding these proteins as part of the proposed clinical trial.

2.3.1.1. Thymidylate Synthase (TS)

TS is the critical enzyme in the de novo synthesis of thymidylate, an essential precursor of thymidine triphosphate, which is required for DNA synthesis and repair [11]. TS is, therefore, an important target for 5-FU as well as other folate-based antimetabolites, and clinical resistance to these TS-targeted agents has been linked to overexpression of TS in tumors (reviewed in [43]).

TS expression is governed at both transcriptional and translational levels. The human *TS* promoter has at least two tandem repeats of a 28-nucleotide G/C-rich sequence in the first 100 nucleotides upstream of the translational start site [22, 27, 77]. These tandem repeat sequences are known as the *TSER* (thymidylate synthase enhancer region). The two most common *TSER* alleles are the two tandem repeats (*TSER*2*) which has an allelic frequency of 0.2 - 0.5 in the Caucasian populations and three tandem repeats (*TSER*3*) with an allelic frequency of 0.5 – 0.8 [42]. The *TSER*3/*3* genotype (~25% frequency in Caucasians) is associated with higher (3-4 fold *in vitro*) TS protein expression. The number of repeated sequences influences the efficiency of translation of human *TS* mRNA [22, 28, 42].

Clinical studies have demonstrated that individuals who were homozygous for *TSER*3* had significantly higher *TS* mRNA expression levels in tumor tissue than those with *TSER*2* alleles and that these findings correlated with a lower response rate to 5-FU [22, 23, 54, 78]. In a study of 221 colorectal cancer patients, individuals with at least one *TSER*2* allele had an improvement in survival with 5-FU treatment (p=0.05) [23]. Similarly a study in rectal cancer patients reported a very significant correlation between *TSER* genotypes and tumor downstaging after preoperative chemoradiation [78]. Together, these studies suggest that *TSER* genotyping may be useful in selecting patients who are likely to respond to treatment with 5-FU or its analogues.

In gastric cancer patients treated with 5-FU and cisplatin, higher tumor TS levels were associated with a less favorable response (29% vs. 68%; p=0.024) [44]. Similarly, in a study in which patients were treated with high dose 5-FU, patients with high TS expression had a response rate of only 12.5%. Conversely a response rate of 92.9% was observed in patients with low tumor TS expression [82]. A longer but not statistically significant survival advantage was observed in patients with the *TSER*2* allele compared with the *TSER*3/*3* patients [24]. **Therefore, the primary goal of this proposal is to prospectively genotype patients, select patients with "good risk" *TSER* genotypes (***TSER*2*/*2* or **2/*3*) **and treat them with a standard 5-FU containing regimen in order to improve clinical outcomes.**

Germline *TSER* status may not always correlate with tumor TS expression levels. A recent study reported a frequent incidence of loss of heterozygosity in tumor tissues from patients with the

heterozygous *TSER*2/*3* genotype [76]. Loss of heterozygosity of the *TSER*2* allele in tumor tissues (tumor genotype of *TSER*3/loss*) was associated with high TS expression in tumor tissues and decreased response rate and survival [76]. We will examine whether the differences in germline vs. tumor *TSER* genotypes can account for clinical responses.

In addition to the number of *TSER* repeat sequences, the presence of a functional SNP (G>C) within the second tandem repeat in the 5'-UTR [29, 41] or a 6-bp deletion in the 3'-UTR has been associated with decreased tumor TS expression [77]. Both of these polymorphisms result in lower TS expression than would be expected by the presence of the *TSER*3* allele alone. We will examine the potential contribution of these additional *TYMS* polymorphisms to the observed study outcome.

2.3.1.2. Dihydropyrimidine dehydrogenase (DPD):

DPD is the principal enzyme responsible for 5-FU catabolism which is the pathway responsible for the elimination of approximately 80% of the administered drug [11]. Therefore, a reduction in this enzyme activity is one of the major factors that influences systemic exposure to fluorodeoxyuridine monophosphate (FdUMP) and the incidence of adverse effects to 5-FU [11]. DPD activity is completely or partially deficient in 0.1% and 3–5% of individuals in the general population, respectively, and DPD deficiency has been associated with severe toxicity and fatal outcomes after 5-FU treatment [38, 45].

DPD deficiency appears to be a genetic disorder arising from multiple polymorphisms in the *DPYD* gene resulting in decreased enzyme activity [70]. Analyses of the prevalence of the various mutations in the *DPYD* allele have shown that a guanidine to adenine point mutation in the invariant splice donor site (*DPYD*2A*) is by far the most common [12]. Polymorphisms in *DPYD* gene will be evaluated as part of the study to potentially account for any unexpected toxicity related to the study treatment.

2.3.2. Oxaliplatin

Oxaliplatin is presumed to exert its cytotoxicity through DNA alkylation similar to other platinums. Oxaliplatin differs from cisplatin by the presence of a diaminocyclohexane ligand. There are no known genetic factors related to oxaliplatin disposition [21, 56, 72]. However, several mechanisms play a role in the resistance to platinum agents and the genes encoding these proteins have demonstrated variations associated with protein activity and subsequent sensitivity to oxaliplatin. These include decreased drug accumulation, drug inactivation, enhanced tolerance to platinum–DNA adducts, and enhanced DNA repair (reviewed in [35]). We will examine variations in the genes encoding proteins involved in these processes as part of the proposed study.

2.3.2.1. Excision Repair Complementation Group 1 (ERCC1):

ERCC1 is an essential member of the nucleotide excision repair (NER) pathway and NER is thought to be the mechanism in mammalian cells for the removal of bulky DNA adducts produced

by platinum agents such as oxaliplatin [81]. *In vitro*, ERCC1 expression in colon cancer cell-lines predict oxaliplatin sensitivity [7]. Clinically, studies have shown an association between ERCC1 expression and response to platinum chemotherapy, in ovarian cancer and colorectal cancer [14, 44]. In other clinical studies, *ERCC1* gene expression levels had a significant correlation with overall survival after 5-FU/oxaliplatin therapy in patients with advanced colorectal cancer refractory to first-line chemotherapy ($p<0.001$) [63]. There are two common polymorphisms of the *ERCC1* gene that have been associated with the clinical outcome of patients with advanced colorectal cancer treated with 5-FU/oxaliplatin [52]. One SNP at codon 118 causes a C→T change without altering amino acid coding. Patients with the C/C genotype had a median survival of 15.3 months versus 7.0 months and 11.1 months for the C/T and T/T genotypes, respectively ($p=0.021$).[50]. The second *ERCC1* single nucleotide polymorphism causes a C→A change and is located in position 8092 in the 3′-UTR. This polymorphism was shown to have some correlation to overall survival, whereby patients with the A allele had a survival benefit ($p=0.08$).

2.3.2.2. Xeroderma Pigmentosum Group D (XPD)/ERCC2

XPD (also known as ERCC2) is a helicase involved in the NER pathway [19]. XPD plays a central role in the recognition of damaged DNA. Several common polymorphisms in the *XPD* gene have been reported to be associated with differential DNA repair capacity [10, 62, 68]. One polymorphism is at codon 751 and causes an A→C change (Lys-751-Gln). A study in patients with colorectal cancer receiving therapy with 5-FU/oxaliplatin reported higher response and survival rates in individuals with the Lys/Lys genotype than individuals who had Lys/Gln or Gln/Gln genotypes ($p=0.002$) [51].

2.3.2.3. X-ray Cross Complementation Group 1 (XRCC1):

XRCC1 is involved in the repair of DNA single-strand breaks via the base excision repair multi-enzyme complex and removes incorrect nucleotides that have been incorporated into DNA due to oxidative damage, and adducts formed after treatment with alkylating agents [64, 74]. Polymorphic changes of the *XRCC1* gene were detected at codons 194 (Arg-Trp), 280 (Arg-His), and 399 (Arg-Gln) [62]. In a study in patients with colorectal cancer treated with chemotherapy consisting of oxaliplatin and continuous infusion 5-FU a significant association between the 399 (Arg- Gln) polymorphism and clinical response to therapy was noted, in that patients carrying the Gln allele were shown to be at a higher risk (5.2 fold) of failing the 5-FU/oxaliplatin chemotherapy ($p=0.038$) [69].

2.3.2.4. Glutathione S-transferase P1 (GSTP1):

GSTP1 belongs to a superfamily of phase II metabolic enzymes and is overexpressed in human colorectal cancer tissues and appears to plays a significant role in detoxification and resistance to platinum agents [20, 46]. A single nucleotide polymorphism, an

A→G substitution at codon 105 (Ile-Val), of the *GSTP1* gene leads to diminished GSTP1 enzymatic activity [79]. In a retrospective analysis of 107 patients with refractory metastatic colorectal cancer treated with 5-FU and oxaliplatin, patients possessing the Val allele had a superior survival benefit [71].

2.4. Rationale

Polymorphic tandem repeat sequences in *TSER* have been shown to correlate with TS mRNA/protein expression and activity. These findings have been extended to the clinical setting, but mainly in a retrospective manner. **We hypothesize that we will achieve a higher response rate than previously demonstrated by selecting treatment for patients with metastatic gastric or GEJ cancer according to a prospective assessment of their *TSER* polymorphism status.** Patients with a *TSER*2* allele (low TS expression genotypes, 5-FU sensitive) will receive a 5-FU containing regimen, FOLFOX-6 (5-FU, leucovorin, oxaliplatin). Patients who have homozygous *TSER*3* genotypes (high TS expression genotypes, 5-FU resistant) will not be included in study. To our knowledge, this will be the first study of this kind in patients with gastric and GEJ adenocarcinomas, testing the clinical utility of *TSER* polymorphism status to guide therapeutic decisions.

To test our hypothesis, we propose to conduct a multi-centered, Phase II clinical trial. The rationale for choosing the germline *TSER* polymorphism status and study treatments is as follows:

- *TSER* polymorphisms have been associated with TS expression and 5-FU efficacy (See Section 2.3.1.).
- *TSER* tandem repeat polymorphisms in germline DNA are relatively common.
  - *TSER**3 (allelic frequency 0.5 – 0.8) vs *TSER**2 (allelic frequency 0.2 – 0.5)
- *TSER* polymorphism status in germline DNA can be rapidly determined prior to chemotherapy treatment (See Section 4.2.).

Despite convincing retrospective studies, there have been no prior prospective trials to test the clinical utility of pharmacogenomically guided treatment in this patient population. We will test the utility of the germline *TSER* tandem repeat status as a predictive marker to save patients from potentially ineffective treatment with 5-FU.

We will also try to identify other potential factors that may alter expected toxicity or effectiveness of the selected treatment regimens. Specifically we will examine the following:

- Loss of heterozygosity in tumor tissues (Section 2.3.1.)
- Genetic variations to potentially account for the toxicity or effectiveness of the selected treatment regimens (e.g. *TYMS, DPYD, ERCC1, ERCC2, XRCC1, GSTP1*).

The frequency of *TSER* tandem repeat polymorphisms makes it possible to test the clinical utility of pharmacogenomically guided therapy in a relatively small sample of patients (n=75). The results of this study will be important to provide solid scientific evidence to justify a large-scale randomized phase III study with the eventual goal of the reliable use of genetic markers in customizing cancer treatment.

3. PATIENT SELECTION

3.1. Eligibility Criteria

3.1.1. Patients must have histologically or cytologically confirmed adenocarcinoma of the stomach or gastroesophageal junction.

3.1.2. Patients must have measurable disease, defined as at least one lesion that can be accurately measured in at least one dimension (longest diameter to be recorded) as $\geq$20 mm with conventional techniques or as $\geq$10 mm with spiral CT scan. See section 9.2 for the evaluation of measurable disease.

3.1.3. No prior therapy for metastatic disease. Prior neo-adjuvant or adjuvant therapy is permitted if the disease free interval has been longer than 6 months.

3.1.4. Age ≥18 years. Because no dosing or adverse event data are currently available on the use of these regimens in patients <18 years of age, children are excluded from this study.

3.1.5. Life expectancy of greater than ≥3 months.

3.1.6. ECOG performance status ≤ 2 (Karnofsky ≥60%; see Appendix A).

3.1.7. Patients must have normal organ and marrow function as defined below:

– Leukocytes	≥3,000/microliter
– Absolute neutrophil count	≥1,500/microliter
– Platelets	≥100,000/microliter
– Total bilirubin	≤ 1.5 x ULN
– AST(SGOT)/ALT(SGPT)	≤ 2.5 x ULN if not liver metastases<br>< 5 x ULN if known liver metastases
– Serum Creatinine	≤ 1.5 x ULN

3.1.8. Not pregnant. Not breast feeding. If the patient or partner is of childbearing potential, the couple will use adequate birth control in accordance with VUMC IRB policies:

For woman of childbearing potential:
Patient must have negative blood pregnancy test. If sexually active, woman must either be post-menopausal (over age 50 and have not had a menstrual period for one year or more, or blood FSH level in the post-menopausal range) OR agree to use appropriate contraceptive measures for the duration of the study and for 21 days after stopping study treatment. The only birth control methods for women that are acceptable for this study are: (1) surgical sterilization (previous removal of the uterus or both ovaries or a tubal ligation) OR (2) an intrauterine device (IUD), (3) double barrier methods, (4) oral contraceptives.

For men:
Medically acceptable contraceptives include: (1) surgical sterilization, or (2) a condom used with a spermicide. If the female partner becomes pregnant while patient is on treatment or within 21 days after stopping treatment, the study physician must be informed.

3.1.9. Ability to understand and the willingness to sign a written informed consent document.

3.2. Exclusion Criteria

3.2.1. Patients may not be receiving any other chemotherapy agents.

3.2.2. Patients with known active brain metastases. Patients with treated brain metastases are permitted if stable off steroids for at least 30 days. A

screening head CT/MRI is not required in asymptomatic patients for this protocol.

3.2.3. History of allergic reactions to 5-FU or oxaliplatin.

3.2.4. Uncontrolled intercurrent illness including, but not limited to, ongoing or active infection, or psychiatric illness/social situations that would limit compliance with study requirements.

3.2.5. Patients with immune deficiency are at increased risk of lethal infections when treated with marrow-suppressive therapy. Therefore, HIV-positive patients receiving combination anti-retroviral therapy are excluded from the study because of possible pharmacokinetic interactions with the chemotherapies.

3.3. ***Inclusion of Women and Minorities***

According to the U.S. Census Bureau, the 2002 population of Tennessee is 5,740,021 with women comprising 51.3% of the residents. The American Cancer Society estimates that 29,100 new cases of cancer will be diagnosed in Tennessee in 2002 roughly half of which will occur in women. However, the annual death from cancer in women is less than that for men living in Tennessee (142.8 vs. 237 per 100,000). Between 1/1/98 and 08/31/03 VICC investigators enrolled over 7,000 patients into VICC clinical trials 60% of whom were entered into therapeutic studies. Of the population enrolled in VICC trials, approximately 6,900 had specific gender data recorded and among this group; there were 3,364 (49%) women and 3,529 (51%) men. These figures are commensurate with the gender distribution in our geographic region indicating that women are appropriately represented in the clinical trials performed at the VICC (Table 3).

According to the 2002 U.S. Census information, the racial composition of Tennessee's 5.7 million citizens consists of 80.2% Caucasians, 16.4% Black or African-American, ~2% Hispanic or Latino and ~2% “other”. By contrast, the racial composition of the VICC referral area, which consists of 68 counties in middle Tennessee, 38 counties in southern and western Kentucky and nine counties in northern Alabama, differs slightly from the state demographics. The population of this geographic region consists of 5.5 million residents with 85.5% Caucasians, 11% Black or African American, 2.2% Hispanic or Latino and 0.2% Asian.

Of the more than 8,000 patients enrolled into VICC clinical trials between 1/1/98 and 2/29/04, approximately 7,200 individuals listed a specific racial group. Among those listing a racial group, 88% were Caucasian, 8% were Black or African-American and 4% were “other” and were mostly comprised of individuals listing themselves as Hispanic or Latino. Slightly fewer than 5% of patients did not provide their racial or ethnic data. Although there has been a decline in minority accrual to therapeutic trials, we have experienced an upswing in minority accrual to non-therapeutic trials. Thus, the overall minority accrual numbers have actually increased slightly from 1998 through 2003. Data from the first two months of 2004 are provided in the updated accrual numbers provided below (Table 4) and show a continuation of this trend.

Women and members of the minority community will be actively recruited into the studies. There are known racial variations in the frequency of *TSER* repeat sequences, where higher numbers of repeats are seen in people of African heritage. Therefore, the inclusion of minorities in the study will be critical to allow for the generalization of the study results and will assist in rapid study accrual.

A number of initiatives to increase minority participation in clinical trials are underway. Most notably, in 2001 the Meharry-Vanderbilt Alliance was formed to enhance

representation of under-represented minorities in clinical trials. The partnership includes several important research and training goals including fostering multidisciplinary research efforts, increasing the representation of minority investigators with R01 or other investigator-initiated support, and expanding access of underserved minorities to clinical trials. The cooperative alliance between the Vanderbilt University Medical Center and the Meharry Medical Center will facilitate the recruitment of members of the minority community.

TABLE 3: FEMALE ENROLLMENT IN VICC CLINICAL TRIALS – 1998-2003

Category	Gender:	1998	1999	2000	2001	2002	2003*	Total:
Therapeutic	Female	309	341	344	330	272	256	1852
	Male	410	363	355	383	457	331	2292
Non-therapeutic	Female	109	217	301	195	281	409	1512
	Male	125	67	199	207	350	289	1237
Total	All	953	988	1199	1115	1360	1285	6893

TABLE 4: MINORITY ENROLLMENT IN VICC CLINICAL TRIALS – 1998-2003

Category	Race:	1998	1999	2000	2001	2002	2003	2004*	Total:
**Therapeutic**	White	611	612	616	637	655	763	95	3989
	Black	76	71	61	50	57	42	8	365
	Other	8	4	14	11	8	12	5	62
**Subtotal**		695	687	691	698	720	817	108	4416
	**Race:**	**1998**	**1999**	**2000**	**2001**	**2002**	**2003**	**2004***	**Total:**
**Non-therapeutic**	White	213	249	174	313	641	1211	207	3008
	Black	17	22	20	20	68	122	31	300
	Other	2	8	6	6	6	17	8	53
**Subtotal**		232	279	200	339	715	1350	246	3361
**Total**	**All**	**927**	**966**	**891**	**1037**	**1435**	**2167**	**354**	**7777**

*data through February 2004

3.4. Research Eligibility Evaluation

Pretreatment

- Complete history and physical examination
- Chemistry panel
- CBC, differential, platelet count
- Urinalysis
- B-HCG for women of child-bearing potential
- ECG (as indicated)
- Baseline CT or MRI of chest, abdomen and pelvis

4. TREATMENT PLAN

4.1. Study Design

The proposed study is a Phase II multi-institutional study for patients with gastric and GEJ tumors where treatment is prospectively determined based on germline *TSER* status. Genetic assessments will be performed at pretreatment and retrospectively, as described in section 4.2.

4.2. ***Study Procedures***

- Prior to treatment, potentially eligible patients will have blood drawn for genotyping. *TSER* status will be assessed (Molecular Diagnostics Laboratory at Washington University Medical Center) and results will be available for treatment assignment within 5 working days. Patients who have *TSER*2/*2* or *TSER*2/*3* genotypes will receive the modified FOLFOX-6 treatment. Patients homozygous for *TSER*3* will not be included in study.

- FOLFOX-6 chemotherapy: oxaliplatin IV in 500 ml D5W over 2 hours, leucovorin calcium IV over 2 hours, and fluorouracil IV over 5 minutes and then continuously over 46 hours on days 1 and 15.

- Treatment courses repeat every 2 weeks +/- 3 days (2 treatments per cycle) in the absence of unacceptable toxicity or disease progression. Disease assessments will be performed after 8 weeks (2 cycles) of treatment.

- Patients will be followed up until death or for 4 years from the date of study registration.

Genotype	Chemotherapy	Dose	Administration	Frequency
TSER *2/*3 or *2/*2	• Oxaliplatin<br>• Leucovorin<br>• 5-FU (bolus)*<br>• 5-FU (infusion)	• 85 mg/m^2<br>• 400 mg/m^2<br>• 400 mg/m^2<br>• 2400 mg/m^2	• IV in 500 ml D5W over 2 h<br>• IV over 2 h<br>• IV push over < 5 min<br>• IV infusion over 46 h	Every 2 weeks

*: bolus 5-FU may be eliminated from the FOLFOX-6 regimen for grade 2 or higher toxicity at the discretion of the investigator

4.3. ***Supportive Care Guidelines***

- Oxaliplatin is moderately emetogenic. A 5HT3 antagonist such as ondansetron 8mg po/iv will be given prior to each treatment. If needed, additional agents may be used, including oral steroids and phenothiazines (e.g. prochloperazine, metochlopramide).

- Magnesium and potassium levels must be within normal limits, and upper normal levels are encouraged due to concerns for ECG changes. The doses/route of electrolyte repletion will be at the discretion of the treating physician.

- For serum potassium level < 3.5 mmol/L, administration of 80 mEq of potassium, given as 40 mEq given IV and 40 mEq given PO is recommended. For patients with serum potassium levels > 3.5 mmol/L but < 4.0 mmol/L, administration of 40 mEq potassium, administered by either oral or IV routes is recommended.

- For patients with a serum magnesium level <0.85 mmol/L, administration of 1 gm $MgSO_4$ IV (8.12 mEq) for every 0.05 below 0.85 mmol/L, with a maximum of 4 grams (32.48 mEq) is recommended.

- Prophylactic (oral) antibiotics such as levofloxacin and (oral) antifungals such as fluconazole are not standardly recommended but are permitted in neutropenic patients (ANC <500).

- G-CSF use is allowed in patients with neutropenic fever according to ASCO guidelines (www.asco.org; guidelines@asco.org). Routine prophylaxis with GCSF is not permitted.

- In general, other concomitant medications and therapies deemed necessary for the supportive care and safety of the patient are allowed. Their use should be documented in the patient records and study specific flow sheets (this includes blood/platelet transfusions for patients with anemia and thrombocytopenia).

- The administration of other anti-neoplastic agents including chemotherapy, radiation therapy and biologic agents is not permitted on this study (except as described above).

- Hypersensitivity: Platinum hypersensitivity can cause dyspnea, bronchospasm, itching and hypoxia. Appropriate treatment includes supplemental oxygen, steroids, antihistamines and epinephrine; bronchodilators and vasopressors may be required. Platinum hypersensitivity is an extremely rare event (approximately 0.5% of patients) and should be treated promptly.

- Pharyngo-laryngeal Dysesthesias: Oxaliplatin may cause discomfort in the larynx or pharnyx associated with dyspnea, anxiety and/or swallowing difficulty and is exacerbated by cold. Appropriate therapy includes use of anxiolytics and cold avoidance. If grade 1 (mild) pharyngo-laryngeal dysesthesias occurs while treatment is being administered, increase the duration of infusion to 6 hours. If grade 2 (moderate) or grade 3 (severe) pharyngo-laryngeal dysesthesias occurs during treatment administration, stop oxaliplatin infusion, administer benzodiazepine, reassure the patient and monitor. At the discretion of the investigator, the infusion may be re-started at 1/3 original infusion rate. Increased duration of infusions is not required for subsequent treatment administration.

- Diarrhea: Patients must be instructed on the prompt initiation of anti-diarrhea therapy at the earliest signs of diarrhea onset. Aggressive re-hydration therapy should be considered for any patient experiencing grade 2 or worse diarrhea. In addition, empiric use of antibiotics directed against enteric bacteria should be considered in individuals who have multiple risk factors for bowel sepsis, such as diarrhea plus neutropenia (even in the absence of fever), or fever plus diarrhea (even in the absence of neutropenia). Loperamide is recommended to treat delayed diarrhea associated with therapy. Patients should begin taking loperamide at the earliest signs of diarrhea (i.e., first poorly formed or loose stool or first episode of an increase from baseline in bowel movements in one day) that occurs in association with therapy. Loperamide should be taken in the following manner: 4 mg at the first onset of diarrhea then 2 mg every two hours around the clock until diarrhea free for at least 12 hours. Patients may take loperamide 4 mg every four hours during the night. In those individuals with loperamide intolerance, the use of other narcotic antiperistaltic agents (e.g., Lomotil, codeine) may be considered at the investigator's discretion.

- The use of other investigational agents is not allowed during this trial.

4.4. Duration of Therapy

In the absence of treatment delays due to adverse event(s), treatment may continue until one of the following criteria applies:

- Disease progression

- Intercurrent illness that prevents further administration of treatment. Where treatment is delayed >28 days, permission of the PI for restarting treatment is required.

- Unacceptable adverse event(s)
- Patient decides to withdraw from the study
- General or specific changes in the patient's condition render the patient unacceptable for further treatment in the judgment of the investigator, or
- Non-compliance with the protocol by the patient or treating physician.

5. DOSING DELAYS/DOSE MODIFICATIONS

5.1. Dose Modifications

All toxicities should be graded according to the Common Terminology Criteria for Adverse Events (CTCAE), version 3.0.

Dose modifications are based upon dose level administered at last treatment day. If the dose level has been reduced due to toxicity, re-escalation is not permitted. If dose reduction is required after dose level -2, patient will discontinue protocol therapy.

5.1.1. Dose levels

Chemotherapy	Starting Dose	-1 Dose Level	-2 Dose Level
**Oxaliplatin**	85 mg/m^2	65 mg/m^2	50 mg/m^2
**Bolus 5-FU**	400 mg/m^2	300 mg/m^2	200 mg/m^2
**Infusion 5-FU**	2400 mg/m^2	1800 mg/m^2	1200 mg/m^2
**Leucovorin**	400 mg/m^2	400 mg/m^2	400 mg/m^2

5.1.2. Dose Modifications for Toxicity Related to FOLFOX-6

Toxicity NCI Grade (Value)	Worst interval toxicity	Day of treatment
No toxicity	Maintain dose level	Maintain dose level
**Neutropenia (ANC)**<br>Grade 1 (ANC < LLN - 1500/mm^3)<br>Grade 2 (ANC <1499 - 1000/mm^3)<br>Grade 3 (ANC <999 - 500/mm^3)<br>Grade 4 (ANC < 500/mm^3)	 Maintain dose level<br>Maintain dose level<br>Maintain dose level<br>Reduce 5-FU and oxaliplatin 1 dose level	If ANC < 1000 on day of treatment, hold and check weekly until > 1000 mm^3. Then treat based on interval toxicity. If ANC < 1000 after 2 weeks, discontinue therapy.
**Thrombocytopenia**<br>Grade 1 (PLT < LLN - 75,000/mm^3)<br>Grade 2 (PLT 74,999 – 50,000/mm^3)<br>Grade 3 (PLT 49,999 – 25,000/mm^3)<br>Grade 4 (PLT< 25,000/mm^3)	 Maintain dose level<br>Maintain dose level<br>Reduce 5-FU and oxaliplatin 1 dose level<br>Reduce 6-FU and oxaliplatin 1 dose level	If PLT < 75,000 on day of treatment, hold and check weekly until > 75,000 mm^3. Then treat based on interval toxicity. If PLT < 75,000 after 2 weeks, discontinue therapy.
**Diarrhea**<br>Grade 1<br>Grade 2<br>Grade 3<br>Grade 4	 Maintain dose level<br>Maintain dose level<br>Reduce 5-FU and oxaliplatin 1 dose level<br>Reduce 5-FU and oxaliplatin 1 dose level	Hold chemotherapy if any grade of diarrhea above baseline is present with the patient not taking antidiarrheal agents within 24 hours of treatment. Reduce 5-FU and oxaliplatin 1 dose level upon resolution of diarrhea. If diarrhea has not resolved within 2 weeks of scheduled treatment day, discontinue therapy.
**Other nonhematologic toxicities** (except Neurologic, alopecia, anorexia, nausea/vomiting if can be controlled by antiemetics)<br>Grade 1<br>Grade 2<br>Grade 3<br>Grade 4	 Maintain dose level<br>Maintain dose level<br>Reduce 5-FU and oxaliplatin 1 dose level<br>Reduce 5-FU and oxaliplatin 1 dose level	 Maintain dose level<br>Hold until resolved to grade <1. Maintain dose level<br>Hold until resolved to grade <1. Reduce 5-FU and oxaliplatin 1 dose level<br>Reduce 5-FU and oxaliplatin 1 dose level

5.1.3. Dose Modifications for Neurologic Toxicity Related to oxaliplatin

**Toxicity (Grade)**	**Duration of Toxicity**		
**Paresthesias/dysesthesias***	1 - 7 Days	> 7 Days	Persistent Between Doses
**Grade 1**<br>Short duration that resolves and does not interfere with function	No change	No change	No change
**Grade 2**<br>Interfering with function, but not activities of daily living (ADL)	No change	No change	Reduce oxaliplatin 1 dose level
**Grade 3**<br>With pain or with functional impairment that also interferes with ADL	1st time: reduce oxaliplatin 1 dose level<br>2nd time: reduce oxaliplatin another dose level	1st time: reduce oxaliplatin 1 dose level<br>2nd time: reduce oxaliplatin another dose level	Discontinue oxaliplatin
**Grade 4**<br>Persistent symptoms that are disabling or life-threatening	Discontinue oxaliplatin	Discontinue oxaliplatin	Discontinue oxaliplatin

6. PHARMACEUTICAL INFORMATION

6.1. __Oxaliplatin__

For complete prescribing information, please refer to the approved package insert.

6.1.1. Other Names: Eloxatin, trans-l-diaminocyclohexane oxalatoplatinum, cis-[oxalato(trans-l-1,2-diaminocyclohexane)platinum(II)].

6.1.2. Classification: Alkylating agent.

6.1.3. Mode of Action: The mechanism of action of oxaliplatin is similar to cisplatin. The main site of action is intrastrand cross-linking, therefore inhibiting DNA replication and transcription.

6.1.4. Storage and Stability: Oxaliplatin vials are stored at room temperature between 20 and 25 ºC protected from light. Reconstituted solution in sterile water or 5% dextrose may be stored for 24 to 48 hours at 2 to 8 ºC. After further dilution in 5% dextrose, the solution is stable for 24 hours at room temperature.

6.1.5. How supplied: Oxaliplatin is commercially available in 50 or 100 mg vials. Oxaliplatin is reconstituted by adding 10 to 20 mL of sterile water or 5% dextrose to the 50 mg vial. Add 20 to 40 mL sterile water or 5% dextrose to the 100 mg vial. Further dilution is needed in 250 - 500 mL of 5% dextrose prior to administration.

6.1.6. Route of Administration: Intravenous

6.1.7. Incompatibilities: Oxaliplatin may degrade in the presence of aluminum-containing needles or IV infusion sets or alkaline medications (such as fluorouracil). Oxaliplatin is incompatible with sodium chloride solutions.

6.1.8. Side effects:

6.1.8.1. Allergy/Immunology: Rhinitis, Allergic/Hypersensitivity reactions (including drug fever). Can be fatal and occur with any cycle of therapy. Manifested by: urticaria, pruritus, flushing of the face, diarrhea (during infusion), shortness of breath, bronchospasm, diaphoresis, chest pains, hypotension, disorientation, and syncope.

6.1.8.2. Auditory: Middle ear/hearing (ototoxicity, mild), inner ear/hearing (mild hearing loss).

6.1.8.3. Blood/Bone Marrow: decreased hemoglobin, hemolysis (e.g. immune hemolytic anemia, drug-related hemolysis), decreased leukocytes, decreased platelets, neutropenia. Single-agent oxaliplatin produces only mild myelosuppression with minimal to severe neutropenia, anemia or thrombocytopenia. In combination, more grade 3/4 neutropenia or thrombocytopenia may be noted.

6.1.8.4. Cardiovascular (Arrhythmia): Sinus tachycardia, supraventricular arrhythmias (SVT/atrial fibrillation/flutter), ventricular arrhythmias (PVCs/ bigeminy/ trigeminy/ventricular tachycardia).

6.1.8.5. Cardiovascular (General): Edema, hypertension, hypotension

6.1.8.6. Coagulation: DIC (disseminated intravascular coagulation), thrombosis/embolism (including pulmonary embolism), prolonged prothrombin time, increased INR, thrombotic microangiopathy (thrombotic thrombocytopenic purpura, hemolytic uremic syndrome). The hemolytic uremic syndrome should be suspected in individuals who experience the following: unexplained severe hemolysis, hemoglobinemia and renal failure as demonstrated by an increase in serum creatinine.

6.1.8.7. Patients suspected of experiencing HUS should have the following laboratory analyses conducted:
Creatinine, BUN
Urinalysis with microscopic evaluation
CBC with differential and platelets
PT/PTT
Fibrinogen, Fibrinogen Degradation Products (FDP)
Anti-thrombin III (ATIII)
Von Willebrand Factor (VWF)
Anti-nuclear antibodies (ANA)
Rheumatoid Factor (RhF)
C3, C4, CH50
Anti-platelet antibodies
Platelet associated IgG
Circulating immune complexes

6.1.8.8. Oxaliplatin should be discontinued for any suspected occurrence of hemolytic uremic syndrome.

6.1.8.9. Constitutional Symptoms: Fever (in the absence of neutropenia, where neutropenia is defined as AGC < 1.0 x 10^9L), fatigue (lethargy, malaise, asthenia), rigors/chills, insomnia, sweating, weight gain, weight loss.

6.1.8.10. Dermatology/Skin: Erythema or skin eruptions, alopecia, hand-foot skin reaction, injection site reaction, rash/desquamation, urticaria, pruritus/itching, dry skin, nail changes, pigmentation changes.

6.1.8.11. Endocrine: Hot flashes/flushes.

6.1.8.12. Gastrointestinal: Anorexia, ascites (non-malignant), colitis, constipation, dehydration, diarrhea, dysphagia, enteritis, esophagitis, flatulence, gastritis, gastrointestinal reflux (heartburn, dyspepsia), ileus (or neuroconstipation), intestinal obstruction, nausea, odynophagia (painful swallowing), stomatitis/pharyngitis (oral/pharyngeal mucositis), taste disturbance (dysgeusia), typhilitis, ulcer, vomiting, xerostomia (dry mouth).

6.1.8.13. Hemorrhage: CNS hemorrhage/bleeding, hemoptysis, hemorrhage/bleeding with grade 3 or 4 thrombocytopenia, melena, GI bleeding, rectal bleeding/hematochesia, pulmonary hemorrhage, vaginal hemorrhage, other (hemorrhage NOS).

6.1.8.14. Hepatobiliary/Pancreas: increased alkaline phosphatase, increased bilirubin, increased GGT (gamma glutamyl transpeptidase), hepatic enlargement, increased SGOT (AST) (serum glutamic oxaloacetic transaminase), increased SGPT (ALT) (serum glutamic pyruvic transaminase), pancreatitis, hepatic venoocclusive disease (manifested by hepatomegaly, ascites, and jaundice).

6.1.8.15. Infection/Febrile Neutropenia: Febrile neutropenia (fever of unknown origin without clinically or microbiologically documented fever (ANC <1.0 x 10^9L fever >38.5°C), infection (documented clinically or microbiologically with grade 3 or 4 neutropenia (ANC <1.0 x 10^9L), infection with unknown ANC, infection without neutropenia.

6.1.8.16. Metabolic/Laboratory: Acidosis (metabolic or respiratory), hypoalbuminemia, hypocalcemia, hyperuricemia, hyperglycemia, hypoglycemia, hypokalemia, hypophosphatemia, hyponatremia, hypomagnesemia

6.1.8.17. Musculoskeletal: Involuntary muscle contractions, trismus.

6.1.8.18. Neurology: Ataxia (incoordination, including abnormal gait), cerebrovascular ischemia, confusion, dizziness, extrapyramidal movements/restlessness, insomnia, mood alteration (depression, anxiety), neuropathy cranial (ptosis), vertigo, acute sensory neuropathy induced or exacerbated by cold (including acute laryngo-pharyngeal dysesthesias, Lhermitte's sign, upper extremity paresthesia), chronic peripheral neuropathy (paresthesias, dysesthesias, hypoesthesias), seizure, somnolence, speech impairment, syncope.

6.1.8.19. Ocular/Visual: Conjunctivitis, vision abnormalities including blindness, optic neuritis, papilledema, hemianopsia, visual field defect, transient blindness.

6.1.8.20. Pain: abdominal pain or cramping, athralgia (joint pain), bone pain, chest pain (non-cardiac and non- pleuritic), headache (including migraine), myalgia (muscle pain including cramps and leg cramps).

6.1.8.21. Pulmonary/Upper Respiratory: Bronchospasm/wheezing, Pulmonary fibrosis, cough, dyspnea (shortness of breath), hiccoughs (hiccups, singultus), pneumonitis/pulmonary infiltrates

(including eosinophilic pneumonia, interstitial pneumonitis, and interstitial lung disease), laryngospasm, nasal cavity/paranasal sinus reactions, voice changes (hoarseness, loss or alteration in voice, laryngitis).

6.1.8.22. Renal/Genitourinary: Increased creatinine, renal failure, urinary retention, urinary urgency, dysuria. Hemolytic Uremic Syndrome (HUS) – see coagulation.

6.1.8.23. Vascular: Phlebitis, thrombosis Also reported on oxaliplatin trials but with the relationship to oxaliplatin still undetermined: tongue paralysis, anemia, aphasia, abnormal hepatic function, hyporeflexia, anxiety, depression, dysarthria, insomnia, increased sweating, rhinitis, epistaxis, gout, pancreatitis, idiopathic thrombocytopenia (5 cases), thrombocytopenia associated with hemolytic anemia (2 cases).

NOTE: Oxaliplatin in combination with other agents could cause an exacerbation of any adverse event currently known to be caused by the other agent, or the combination may result in events never previously associated with either agent.

6.1.9. Nursing/Patient Implications:
Premedicate with antiemetics (5 HT3 antagonist and steroid) to prevent severe nausea and vomiting.
Monitor for diarrhea and treat symptomatically.
Monitor for neuropathies (parasthesias of hands, feet and toes, and of pharynx and occasionally cramps), if they occur, tend to be brief (less than one week) during the first course but longer with subsequent courses. Advise patients to avoid cold exposure and against touching cold objects. Sensory neuropathies develop with continued treatment. Ask patient if changes in ambulation, swallowing, breathing or fine motor activity have been noted.
Prolonging the Oxaliplatin infusion time to 6 hours may alleviate acute neurologic toxicities.
Monitor for respiratory changes such as shortness of breath.

6.1.10 WARNING: The hemolytic uremic syndrome should be suspected in individuals who experience the following: unexplained severe hemolysis, hemoglobinemia, and renal failure as demonstrated by an increase in serum creatinine.

6.2. __Fluorouracil__

For complete prescribing information, please refer to the approved package insert.

6.2.1. Other Names: 5-Fluorouracil, 5-FU, Adrucil, Efudex.

6.2.2. Classification: Antimetabolite.

6.2.3. Mode of Action: Fluorouracil is a pyrimidine antagonist that interferes with nucleic acid biosynthesis. The deoxyribonucleotide of the drug inhibits thymidylate synthetase, thus inhibiting

6.2.4. The formation of thymidylic acid from deoxyuridylic acid, thus interfering in the synthesis of DNA. It also interferes with RNA synthesis.

6.2.5. Storage and Stability: Stable for prolonged periods of time at room temperature if protected from light. Inspect for precipitate; if apparent, agitate

vial vigorously or gently heat to not greater than 140EF in a water bath. Do not allow to freeze.

6.2.6. Administration: IV bolus, and IV continuous infusion.

6.2.7. Incompatibilities: Incompatible with doxorubicin and other anthracyclines. When giving doxorubicin IV push or through a running IV, flush line before giving fluorouracil.

6.2.8. How supplied: Commercially available in 500 mg/10 ml ampules and vials, and 1 gm/20 ml, 2.5 gm/50 ml, and 5 gm/100 ml vials.

6.2.9. Side Effects:

6.2.9.1. Hematologic: Leukopenia, thrombocytopenia, anemia, can be dose limiting; less common with continuous infusion.

6.2.9.2. Dermatologic: Dermatitis, nail changes, hyperpigmentation, Hand-Foot Syndrome with protracted infusions, alopecia.

6.2.9.3. Gastrointestinal: Nausea, vomiting, anorexia, diarrhea, can be dose limiting; mucositis, more common with 5-day infusion, occasionally dose limiting; severe, cholera-like diarrhea which can be fatal when given with leucovorin.

6.2.9.4. Neurologic: Cerebellar Syndrome (headache and cerebellar ataxia).

6.2.9.5. Cardiac: Angina, noted with continuous infusion.

6.2.9.6. Ophthalmic: Eye irritation, nasal discharge, watering of eyes, blurred vision.

6.2.9.7. Hepatic: Hepatitis with hepatic infusion.

6.2.10. Nursing/Patient Implications:

- Monitor CBC, platelet counts.
- Administer antiemetics as indicated.
- Monitor for diarrhea. Encourage fluids and treat symptomatically - may be dose imiting.
- Assess for stomatitis - oral care recommendations as indicated. (Do NOT use ce chips)
- Monitor for neurologic symptoms (headache, ataxia).
- Patients on continuous infusions may need instruction regarding central IV atheters and portable IV or IA infusion devices.
- Inform patient of potential alopecia.

6.3. ***<u>Leucovorin Calcium</u>***

For complete prescribing information, please refer to the approved package insert.

6.3.1. Other Names: Leucovorin, Wellcovorin, citrovorum factor, folinic acid, 5-formyl tetrahydrofolate, LV, LCV.

6.3.2. Classification: Tetrahydrofolic acid derivative.

6.3.3. Mode of Action: Leucovorin acts as a biochemical cofactor for 1-carbon transfer reactions in the synthesis of purines and pyrimidines. Leucovorin does not require the enzyme dihydrofolate reductase (DHFR) for conversion

to tetrahydrofolic acid. The effects of methotrexate and other DHFR-antagonists are inhibited by leucovorin. Leucovorin can potentiate the cytotoxic effects of fluorinated pyrimidines (i.e., fluorouracil and floxuridine). After 5-FU is activated within the cell, it is accompanied by a folate cofactor, and inhibits the enzyme thymidylate synthetase, thus inhibiting pyrimidine synthesis. Leucovorin increases the folate pool, thereby increasing the binding of folate cofactor and active 5-FU with thymidylate synthetase.

6.3.4. Storage and Stability: All dosage forms are stored at room temperature. The reconstituted parenteral solution, 10 mg/ml, is stable for at least 7 days at room temperature. At concentrations of 0.5-0.9 mg/ml, the drug is chemically stable for at least 24 hours at room temperature under normal laboratory light.

6.3.5. How supplied: Commercially available in parenteral formulations (3 and 5 mg ampule; 50 mg, 100 mg and 350 mg vial).The 50 and 100 mg vials for injection are reconstituted with 5 and 10 ml of sterile water or bacteriostatic water, respectively, resulting in a 10 mg/ml solution. The 350 mg vial is reconstituted with 17 ml of sterile water resulting in a 20 mg/ml solution.

6.3.6. Administration: IV over 120 minutes

6.3.7. Compatibilities: Leucovorin (0.5-0.9 mg/ml) is chemically stable for at least 24 hours in normal saline, 5% dextrose, 10% dextrose, Ringer's injection or lactated Ringer's injection. Leucovorin (0.03, 0.24 and 0.96 mg/ml) is stable for 48 hours at room and refrigeration temperatures when admixed with floxuridine (FUDR, 1, 2 and 4 mg/ml) in normal saline. Leucovorin is compatible with fluorouracil and oxaliplatin.

6.3.8. Side Effects:

- Hematologic: Thrombocytosis.
- Dermatologic: Skin rash.
- Gastrointestinal: Nausea, upset stomach, diarrhea.
- Allergic: Skin rash, hives, pruritus.
- Pulmonary: Wheezing (possibly allergic in origin).
- Other: Headache; may potentiate the toxic effects of fluoropyrimidine therapy, resulting in increased hematologic and gastrointestinal (diarrhea, stomatitis) adverse effects.

6.3.9. Nursing/Patient Implications:
Observe for sensitization reactions.
When given with fluoropyrimidines, monitor closely for diarrhea and stomatitis.

7. Rationale for Secondary Objectives

The rationale for treating patients according to their *TSER* polymorphism status is described in Section 2.4. Several convincing retrospective studies have indicated the value of germline *TSER* status in predicting the response to 5-FU. Prospective evaluation of *TSER* has been limited and the therefore the scientific validity of *TSER* status as a single predictor of 5-FU sensitivity remains unproven.

Previous retrospective studies have reported variable response rates to 5-FU in groups of patients with different *TSER* genotypes. In the proposed study, we will evaluate the contribution of other genetic factors that may alter the expected outcomes of this genomically based treatment approach (e.g. loss of heterozygosity of a tumor TS allele and variations in other genes involved in response/toxicity of the administered treatment.

7.1. Loss of TSER heterozygosity (LOH) in tumor tissues

TABLE 5. Estimated frequency and response rate for tumor TSER genotype subgroups

Germline genotype	*2/*3(n= 50)		
Tumor genotype	*2/loss	*2/*3	*3/loss
Estimated frequency	46%	22%	32%
Estimated # of patients	23	11	16
Estimated response rate	80%	40%	20%
Estimated response rate	65%		20%

Of the 75 patients in the study, approximately 50 will have heterozygous *TSER**2/*3 genotypes. It has been observed that loss of heterozygosity at the tumor *TS* locus occurs relatively frequently (nearly 70%) [76] where the tumors in one-third of the heterozygous patients will still have the *TSER**2/*3 genotype. The remaining patients will have tumor genotypes of either *TSER**2/loss or *TSER**3/loss genotype. LOH potentially impacts the utility of germline *TSER* polymorphisms as a predictor of 5-FU sensitivity in that heterozygous patients whose tumors lose the *TSER*2* allele (*TSER*3/loss*) have a treatment outcome similar to patients homozygous for *TSER*3* [76]. Conversely, heterozygous patients whose tumors lose the *TSER**3 allele (*TSER*2/loss*) have a treatment outcome similar to patients homozygous for *TSER*2*. This scenario may account for apparent discrepancies in the predictive value of germline *TSER* genotypes.

7.2. Tumor TS mRNA and protein expression

Differing results have been reported by clinical and molecular studies attempting to show an association among TS mRNA [28], protein expression [57], response to treatment, survival, and down-staging of tumors in patients receiving 5-FU treatment [23, 30, 78]. Therefore we will assess the tumoral expression of TS at the mRNA and protein levels in a central laboratory. The results will be correlated with germline and tumor *TSER* genotypes as well as response to the study treatment regimens.

7.3. Assessment of polymorphisms in other genes associated with treatment outcomes or toxicity

In addition to TS, other genetic factors may contribute to any observed variability. We will assess patients for the presence of the most common polymorphisms in genes involved in the disposition or response of 5-FU and oxaliplatin. In many cases these polymorphisms have retrospectively been associated with altered treatment outcomes.

8. STUDY CALENDAR

Baseline evaluations are to be conducted within 2 weeks prior to administration of protocol therapy. Scans and x-rays must be done ≤ 4 weeks prior to the start of therapy. In the event that the patient's condition is deteriorating, laboratory evaluations should be repeated within 48 hours prior to initiation of the next cycle of therapy.

	Pre-treatment	C1 W1	C1 W2	C1 W3	C1 W4	C2 W1	C2 W2	C2 W3	C2 W4	Q1 Cycle	Off Study
FOLFOX-6		X[g]		X[g]		X[g]		X[g]		X[e]	
Informed consent	X										
Demographics	X										
Medical history	X										
Concurrent meds	X	X		X		X		X		X	
Physical exam	X	X		X		X		X		X	X
Vital signs	X	X		X		X		X		X	X
Height	X										
Weight	X	X		X		X		X		X	X
Performance status	X	X		X[f]		X		X[f]		X[f]	X
CBC w/diff, plts	X	X		X		X		X		X	X
Serum chemistry[a]	X	X		X[f]		X		X[f]		X[f]	X
CEA[d]	X										
ECG (as indicated)	X										
Adverse event evaluation		X		X		X		X		X	X
Tumor measurements	X	Tumor measurements should be performed every 8 weeks.									X
Radiologic evaluation	X	Radiologic measurements should be performed every 8 weeks.									X
B-HCG[b]	X										

a: Albumin, alkaline phosphatase, total bilirubin, bicarbonate, BUN, calcium, chloride, creatinine, glucose, LDH, phosphorus, potassium, total protein, SGOT[AST], SGPT[ALT], sodium.
b: Serum pregnancy test (women of childbearing potential).
c. Patient will be followed for every 6 months for 4 years.
d. CEA at baseline and if not normal then at each restaging visit with the CT scan
e. Two treatments per cycle in the absence of unacceptable toxicity or disease progression.
f. Optional. It's done only under physician's discretion.

g. +/- 3 days will be allowed outside of the two week treatment window

9. MEASUREMENT OF EFFECT

For the purposes of this study, patients should be reevaluated for response every 8 weeks. In addition to a baseline scan, confirmatory scans should also be not less than 4 weeks following initial documentation of objective response.

9.1. Definitions

Response and progression will be evaluated in this study using the international criteria proposed by the Response Evaluation Criteria in Solid Tumors (RECIST) Committee [73]. Changes in only the largest diameter (unidimensional measurement) of the tumor lesions are used in the RECIST criteria. Note: Lesions are either measurable or non-measurable using the criteria provided below. The term “evaluable” in reference to measurability will not be used because it does not provide additional meaning or accuracy.

9.1.1. Measurable disease

Measurable lesions are defined as those that can be accurately measured in at least one dimension (longest diameter to be recorded) as $\geq$20 mm with conventional techniques (CT, MRI, x-ray) or as $\geq$10 mm with spiral CT scan. All tumor measurements must be recorded in millimeters (or decimal fractions of centimeters).

9.1.2. Non-measurable disease

All other lesions (or sites of disease), including small lesions (longest diameter <20 mm with conventional techniques or <10 mm using spiral CT scan), are considered non-measurable disease. Bone lesions, leptomeningeal disease, ascites, pleural/pericardial effusions, lymphangitis cutis/pulmonis, inflammatory breast disease, abdominal masses (not followed by CT or MRI), and cystic lesions are all non-measurable.

9.1.3. Target lesions

All measurable lesions up to a maximum of five lesions per organ and 10 lesions in total, representative of all involved organs, should be identified as **target lesions** and recorded and measured at baseline. Target lesions should be selected on the basis of their size (lesions with the longest diameter) and their suitability for accurate repeated measurements (either by imaging techniques or clinically). A sum of the longest diameter (LD) for all target lesions will be calculated and reported as the baseline sum LD. The baseline sum LD will be used as reference by which to characterize the objective tumor response.

9.1.4. Non-target lesions

All other lesions (or sites of disease) should be identified as **non-target lesions** and should also be recorded at baseline. Non-target lesions include measurable lesions that exceed the maximum numbers per organ or total of all involved organs as well as non-measurable lesions. Measurements of these lesions are not required, but the presence or absence of each should

be noted throughout follow-up.

9.2 . Guidelines for Evaluation of Measurable Disease

All measurements should be taken and recorded in metric notation using a ruler or calipers. All baseline evaluations should be performed as closely as possible to the beginning of treatment and never more than 4 weeks before the beginning of the treatment.

Note: Tumor lesions that are situated in a previously irradiated area are not be considered measurable unless new measurable lesions have arisen AFTER completion of XRT.

The same method of assessment and the same technique should be used to characterize each identified and reported lesion at baseline and during follow-up. Imaging-based evaluation is preferred to evaluation by clinical examination when both methods have been used to assess the antitumor effect of a treatment.

9.2.1. Clinical lesions

Clinical lesions will only be considered measurable when they are superficial (e.g., skin nodules and palpable lymph nodes). In the case of skin lesions, documentation by color photography, including a ruler to estimate the size of the lesion, is recommended.

9.2.2. Chest x-ray

Lesions on chest x-ray are acceptable as measurable lesions when they are clearly defined and surrounded by aerated lung. However, CT is preferable.

9.2.3. Conventional CT and MRI

These techniques should be performed with cuts of 10 mm or less in slice thickness contiguously. Spiral CT should be performed using a 5 mm contiguous reconstruction algorithm. This applies to tumors of the chest, abdomen, and pelvis. Head and neck tumors and those of extremities usually require specific protocols.

9.2.4. Bone Scan and PET Imaging

When the primary endpoint of the study is objective response evaluation, PET, Bone Scan, and other nuclear medicine tests should not be used to measure tumor lesions. Two new lesions are considered highly suggestive of progression. These modalities can be used to confirm clinical disease progression where this is ambiguous CT/MRI data, and to confirm complete responses (where baseline bone scan/pet was positive).

9.2.5. Ultrasound (US)

When the primary endpoint of the study is objective response evaluation, US should not be used to measure tumor lesions. It is, however, a possible alternative to clinical measurements of superficial palpable lymph nodes, subcutaneous lesions, and thyroid nodules. US might also be useful to confirm the complete disappearance of superficial lesions usually assessed by clinical examination.

9.2.6. Endoscopy, Laparoscopy

The utilization of these techniques for objective tumor evaluation has not yet been fully and widely validated. Their uses in this specific context require sophisticated equipment and a high level of expertise that may only be available in some centers. Therefore, the utilization of such techniques for objective tumor response should be restricted to validation purposes in reference centers. However, such techniques may be useful to confirm complete pathological response when biopsies are obtained.

9.2.7. Tumor markers

Tumor markers alone cannot be used to assess response. If markers are initially above the upper normal limit, they must normalize for a patient to be considered in complete clinical response. Specific additional criteria for standardized usage of prostate-specific antigen (PSA) and CA-125 response in support of clinical trials are being developed.

9.2.8. Cytology, Histology

These techniques can be used to differentiate between partial responses (PR) and complete responses (CR) in rare cases (e.g., residual lesions in tumor types, such as germ cell tumors, where known residual benign tumors can remain).

The cytological confirmation of the neoplastic origin of any effusion that appears or worsens during treatment when the measurable tumor has met criteria for response or stable disease is mandatory to differentiate between response or stable disease (an effusion may be a side effect of the treatment) and progressive disease.

9.3. Response Criteria

9.3.1. **Evaluation of target lesions**

Complete Response (CR):	Disappearance of all target lesions
Partial Response (PR):	At least a 30% decrease in the sum of the longest diameter (LD) of target lesions, taking as reference the baseline sum LD
Progressive Disease (PD):	At least a 20% increase in the sum of the LD of target lesions, taking as reference the smallest sum LD recorded since the treatment started or the appearance of one or more new lesions
Stable Disease (SD):	Neither sufficient shrinkage to qualify for PR nor sufficient increase to qualify for PD, taking as reference the smallest sum LD since the treatment started

9.3.2. **Evaluation of non-target lesions**

Complete Response (CR):	Disappearance of all non-target lesions and normalization of tumor marker level
Incomplete Response/ Stable Disease (SD):	Persistence of one or more non-target lesion(s) and/or maintenance of tumor marker level above the normal limits

Progressive Disease (PD): Appearance of one or more new lesions and/or unequivocal progression of existing non-target lesions

Although a clear progression of "non-target" lesions only is exceptional, in such circumstances the opinion of the treating physician should prevail, and the progression status should be confirmed at a later time by the review panel (or study chair).

Note: If tumor markers are initially above the upper normal limit, they must normalize for a patient to be considered in complete clinical response.

9.3.3. **Evaluation of best overall response**

The best overall response is the best response recorded from the start of the treatment until disease progression/recurrence (taking as reference for progressive disease the smallest measurements recorded since the treatment started). The patient's best response assignment will depend on the achievement of both measurement and confirmation criteria (see section 9.3.1).

Target Lesions	Non-Target Lesions	New Lesions	Overall Response
CR	CR	No	CR
CR	Incomplete response/SD	No	PR
PR	Non-PD	No	PR
SD	Non-PD	No	SD
PD	Any	Yes or No	PD
Any	PD	Yes or No	PD
Any	Any	Yes	PD

Note:

- Patients with a global deterioration of health status requiring discontinuation of treatment without objective evidence of disease progression at that time should be classified as having "symptomatic deterioration." Every effort should be made to document the objective progression, even after discontinuation of treatment.
- In some circumstances, it may be difficult to distinguish residual disease from normal tissue. When the evaluation of complete response depends on this determination, it is recommended that the residual lesion be investigated (fine needle aspirate/biopsy) before confirming the complete response status.

9.4 . Confirmatory Measurement/Duration of Response

9.4.1. **Confirmation**

To be assigned a status of PR or CR, changes in tumor measurements must be confirmed by repeat assessments that should be performed no less than 4 weeks after the criteria for response are first met. In the case of SD, follow-up

measurements must have met the SD criteria at least once after study entry at a minimum interval of not less than 6-8 weeks (see section 9.3.3).

9.4.2. **Duration of overall response**

The duration of overall response is measured from the time measurement criteria are met for CR or PR (whichever is first recorded) until the first date that recurrent or progressive disease is objectively documented (taking as reference for progressive disease the smallest measurements recorded since the treatment started).

The duration of overall CR is measured from the time measurement criteria are first met for CR until the first date that recurrent disease is objectively documented.

9.4.3. **Duration of Stable Disease**

Stable disease is measured from the start of the treatment until the criteria for progression are met, taking as reference the smallest measurements recorded since the treatment started.

9.5. Progression-Free Survival

Time to progression or death, or progression-free survival (PFS), is one of the primary endpoints of this study. PFS is defined as the duration of time from start of treatment to time of progression or death, whichever occurs first.

9.6. Response Review

All responses will be reviewed by an expert radiologist at the coordinating center (Vanderbilt University).

10. Data Safety and Monitoring Plan

A list of patients participating in this study will be reviewed monthly, or sooner if events arise. Study related serious adverse events will be reviewed in detail with the investigators. Any serious adverse events experienced by patients participating in this trial will be immediately reported to the VICC Clinical Trials Office, and subsequently submitted to the VUMC IRB, and the study sponsor, in this case the NCI. Any unanticipated serious adverse events, including clinical laboratory abnormalities occurring in a patient after providing informed consent, whilst participating in these studies and until 4 weeks after completion of the study related procedures would be reported. A serious adverse event (SAE) report form will be completed and submitted to the Vanderbilt IRB. Each serious adverse event will also be described by: 1.) its duration (start and end dates); 2.) its severity grade (Grade 1 - 4); 3.) its relationship to study participation (suspected / not suspected); 4.) and the action(s) taken. Additionally follow-up will describe whether the event has resolved or continues. The study investigators will determine an assessment as to whether the serious adverse event requires an amendment to the consent form. This assessment will be submitted to each institution's IRB along with the SAE and the IRB will then assess independently whether an amendment to the consent process is warranted. If at any point the investigators, or any of the monitoring, or sponsoring bodies deems this study to be unsafe, accrual will be halted immediately.

Reporting of SAEs

In the event of a serious adverse event, whether related to study procedures or not, the study personnel at the individual sites will notify Vanderbilt personnel by fax or telephone

within 24 hours of being aware of the SAE. Individual sites must complete the following:

1) MedWatch FDA Form 3500, obtain from http://www.fda.gov/medwatch/index.html
2) Vanderbilt Serious Adverse Event Form (in appendix) which is to be signed by the treating physician

Within 24 hours of knowledge of the SAE, the individual site must fax the MedWatch FDA Form 3500 to the FDA and also to Sanofi-Aventis, US Inc. at Pharmacovigilance and Epidemiology at 908-231-4827.

Individual site must immediately send to Vanderbilt the following:

1) Copies of the MedWatch and proof of submission to the FDA and to Sanofi
2) Physician signed copy of Vanderbilt Serious Adverse Event Form
3) IRB Approval of the SAE submitted at the individual site
4) Additional follow-up MedWatch forms sent to the FDA and Sanofi must also be sent to Vanderbilt

Attn: Research Coordinator GI -0716
Vanderbilt-Ingram Cancer Center
2220 Pierce Ave, 491 PRB
Nashville, TN 37232-6868

Phone: 615-936-5747
Fax: 615-936-5850

Vanderbilt, the Coordinating Center, needs to report to the Vanderbilt IRB all adverse events and unanticipated problems involving risk to participants or others at collaborating institutions using the "Report of Adverse Events and Unanticipated Problems Involving Risk to Participants or Others" (Vanderbilt IRB Form #1105).

MedWatch 3500 Reporting Guidelines:

In addition to completing appropriate patient demographic and suspect medication information, the report should include the following information within the Event Description (section 5) of the MedWatch 3500 form:

Treatment regimen (dosing frequency, combination therapy)

Protocol description (and number, if assigned)

Description of event, severity, treatment, and outcome, if known

Supportive laboratory results and diagnostics

Investigator's assessment of the relationship of the adverse event to each investigational product and suspect medication

Follow-up information:

Additional information may be added to a previously submitted report by any of the following methods:

Adding to the original MedWatch 3500 report and submitting it as follow-up

Adding supplemental summary information and submitting it as follow-up with the original MedWatch 3500A form

Summarizing new information and faxing it with a cover letter including subject identifiers (i.e. DOB, subject number), protocol description and number if assigned, brief adverse event description, and notation that additional or follow-up information is being submitted (The subject identifiers are important so that the new information is added to the correct initial report)

Occasionally Vanderbilt may contact the reporter for additional information, clarification, or current status of the subject for whom and adverse event was reported. For questions regarding SAE reporting, you may contact the Vanderbilt-Ingram Cancer Center Clinical Trials Shared Resources office.

Assessing Causality:

Investigators are required to assess whether there is a reasonable possibility that the combination caused or contributed to an adverse event. The following general guidance may be used.

Yes: if the temporal relationship of the clinical event to drug administration makes a causal relationship possible, and other drugs, therapeutic interventions or underlying conditions do not provide a sufficient explanation for the observed event.

No: if the temporal relationship of the clinical event to drug administration makes a causal relationship unlikely, or other drugs, therapeutic interventions or underlying conditions provide a sufficient explanation for the observed event.

10.1. Institutional Monitoring and Data Management

The Vanderbilt-Ingram Cancer Center (VICC) places the highest priority on ensuring the safety of patients participating in clinical trials. From its inception, the VICC has had in place programs that help ensure the utmost protection and safety of all human subjects. Furthermore, even prior to the renewed emphasis on patient safety and data monitoring, the VICC leadership had in place a number of safeguards and oversight committees to ensure all studies conducted under the auspices of the VICC meet the highest standards of clinical research. As a consequence of this long-standing commitment to clinical research excellence, every therapeutic intervention trial conducted at the VICC must include a plan for safety and data monitoring. The VICC Data and Safety Monitoring Plan has been revised using guidelines as outlined in the Essential Elements of a Data Safety and Monitoring Plan for Clinical Trials Funded by the NCI.

At the VICC, monitoring of data and safety of all clinical trials, including those funded by the NCI, involves multiple levels of institutional oversight. The various levels of oversight include the IRB for the protection of human subjects, the VICC SRC through a subcommittee devoted exclusively to data safety and monitoring (the Data Safety and Monitoring Committee), individual disease-oriented and modality-oriented research teams (e.g., thoracic oncology; breast cancer; radiation therapy, etc.) and the diligence of individual Principal Investigators. Where appropriate and applicable, Cooperative Group auditing and reporting systems are used to insure the safety of study patients and validity of study data.

Institutional oversight and monitoring for patient safety and data validity for all cancer clinical trials regardless of funding source is provided by the following institutional committees: a) the Scientific Review Committee by and through its Data and Safety Monitoring Committee, b) Institutional Review Board for the protection of human subjects, and c) General Clinical Research Center Scientific Advisory Committee where appropriate.

10.1.1. Scientific Review Committee (SRC)

All proposed clinical studies undergo a rigorous review that includes scrutiny of the following elements: 1) scientific rational, including appropriate references to medical literature, 2) study design, including adequacy of the scientific aims, eligibility criteria, study endpoints, and treatment information, 3) biostatistical design, 4) proposed study duration, 5) evidence of ability to accrue to the protocol, 6) scientific priority, and 7) adequacy of data collection forms. Cooperative group and other NCI-sponsored studies, which have previously undergone a stringent review process, do not undergo review by full committee, but do undergo administrative review and approval by the SRC Chair and must undergo thorough review by the appropriate disease- or modality-oriented research team.

10.1.1.1. Data and Safety Monitoring Committee (DSMC)

DSMC will prepare or ensure the establishment of a plan for data and safety monitoring for all interventional trials. The committee will conduct or delegate ongoing monitoring of interventional trials and ensure that monitoring is timely and effective and that those responsible for monitoring have the appropriate expertise to accomplish its mission. Additional responsibilities include overseeing monitoring activities and responding to recommendations that emanate from monitoring activities. Oversight of data and safety monitoring will be the responsibility of the VICC SRC through its DSMC. Additionally, the DSMC of the VICC SRC will submit an annual report to the VUMC IRB, VICC Director, and VICC Associate Director for Clinical Research on activities of the preceding year and will make recommendations to improve data and safety monitoring activities as needed.

10.1.2. Institutional Review Board (IRB)

All cancer-related research involving human subjects, and all other activities which even in part involve such research, regardless of sponsorship, must be reviewed and approved by the Vanderbilt University IRB. No intervention or interaction with human subjects in research, including recruitment, may begin until the IRB has reviewed and approved the research protocol according to VU IRB policies and procedures.

All data safety and monitoring reports for local, investigator-initiated studies include the number of patients entered, number of patients treated, dose level of agent(s) involved, summary of all adverse events reported to date using CTC 3.0 grading, a specific list of adverse events requiring expedited reporting to include all serious adverse events (SAEs), and, on an annual basis or as it arises, significant literature reporting developments that may affect the safety of participants or the ethics of the study. On the anniversary date of the initial IRB approval for each local, investigator-initiated clinical trial,

the principal investigator is required to submit to the IRB and the SRC, an annual data safety and monitoring report summarizing the study's safety experience and efficacy over the preceding year and to date.

The IRB is comprised of four separate committees, all of which are appointed as University Committees. The IRB Committees serve Vanderbilt University as a whole, rather than a particular school or department. The Institutional Review Board is comprised of the following committees.

10.1.2.1. Committees for Health Sciences (CHS)

These committees review projects that entail physiological risks to human subjects through treatment or administration of investigational drugs or devices. Due to the volume of clinical research studies conducted at Vanderbilt University, three separate committees were created even though the activities of the individual committees are identical in scope. The availability of three separate committees helps insure no one committee or any individual member of a single committee is overwhelmed with review or oversight. Of note, the General Clinical Research Center (GCRC) utilizes a separate review committee to review all studies to be conducted in the GCRC. However, these studies must also be reviewed and approved by one of the CHS IRB committees prior to activation within the GCRC.

10.1.2.2. Committee for Behavioral Sciences

This committee reviews nonphysical projects that may entail psychological or sociological risks.

The IRB Office is also responsible for the operational support, initial and ongoing training, and oversight of the Human Subjects Radiation Committee, the Radioactive Drug Research Committee, and Institutional Biosafety Committee.

The research plan for safety monitoring, reporting of adverse events, descriptions of interim safety reviews and the procedures planned for transmitting the results to the IRB must be described in the initial IRB application, including a description of independent safety monitoring board (DSMB) or an explanation if it is determined an independent safety monitor is not necessary.

10.1.3. GCRC

The primary objective of the Vanderbilt General Clinical Research Center (GCRC) is to provide space, hospitalization cost, laboratories, equipment and supplies for clinical research by any qualified member of the faculty in any department of the Medical School. The use of the Center is justified on the basis of three criteria: first, the quality and significance of the research; second, the special need for the Center's common facilities; third, the common usefulness or collective justification for facilities or personnel. For this study, the Vanderbilt GCRC will provide service for the DNA extraction only.

All local, investigator-initiated therapeutic clinical trials are required to have specific data safety and monitoring plans based on the size and complexity of each trial.

Data safety and monitoring activities for each study continues until all patients have completed their treatment and all patients are beyond the time point at which study-related adverse events would likely be encountered. In some trials, this requires life-time follow-up.

All SAEs experienced by patients participating in trials conducted under the auspices of the VICC will be immediately reported to the VICC Clinical Trials Office, the VUMC IRB, and the study supporter, Sanofi-Aventis, including those funded by the NCI.

Data management for the therapeutic trials related to this proposal will be performed by the established teams of research nurses and data managers in the Vanderbilt-Ingram Cancer Center Clinical Trials Office (VICC-CTO). All relevant patient information will be maintained on study specific databases customized to ensure patient confidentiality and to handle special electronic data reporting requirements. These databases are linked to the Cancer Center and the Medical Center information systems allowing for efficient downloading of relevant information, cost reduction and error minimization. Additionally clinical and accrual information will be updated in real time.

10.2. ***Confidentiality***

The results of these studies may be published, but without patient identifiers. Efforts will be made to keep the patients' personal information confidential through assignment of identification numbers and maintenance of study related data in a secured database (Oncore described below). However, absolute confidentiality cannot be guaranteed. Patient personal information (research/medical records) may be disclosed if required by law to organizations such as the FDA or NCI for quality assurance and data analysis.

10.3. ***Oncore Database***

A web based study management and reporting database system designed for clinical trial data monitoring. The system was developed as part of a multi-center Oncology Collaborative Research Environment (Oncore) for cancer centers based on shared policies, practices and informatics. The partnership is dedicated to the creation and continuous improvement of technological integration into clinical trial management and monitoring with particular emphasis on multi-center operational environments. The system enables and promotes collaborative cancer research both within and among institutions by allowing for protocol tracking, patient registration, NCI reporting, committee management, SAE tracking, clinical study data capture, electronic case report form design, as well as protocol and regulatory compliance monitoring.

The Oncore database provides cancer centers with the comprehensive set of integrated capabilities including.

- Sophisticated security: dynamic, context-driven access control enables compliance with "good practice" security standards and HIPAA regulations.
- Data and safety monitoring: sophisticated monitoring with cross study toxicity reports, safety-monitoring rules, and automated e-mail notifications.
- Preactivation track and manage scientific review, data and safety monitoring plan, biosafety, resource evaluation, and ethical review (IRB).

- Reporting easy extraction of data for analysis and an assortment of reports and options that maintain critical security and access control policies.
- Study setup and activation rapid setup by non-technical staff, a hierarchy of "telescoping" standards still allow customization of electronic web based forms and schedules.
- Domain repository, an extensible set of standard reference codes, forms and form elements to promote standardization while allowing flexibility and adaptation to new science.
- Data management and collection highly efficient yet easily mastered data entry, patient registration and data management process.
- Public access controllable public web access to protocol information.
- Study monitoring track and monitor protocol accrual, monitor site performance, and support administrative and regulatory reporting needs.
- Application configuration processes, notifications, and nomenclature can be configured to suit each center.

10.4. <u>Data Sharing Plan</u>

10.4.1. Participating sites:

VICC
WUMC
UAB
UNC

10.4.2. Database Training

The initial steps will involve database training at the participating sites either via a web-based conference or on-site training of the treating physician or their study designee at the coordinating center. At the time of training, all participating physicians and/or their designees will receive instruction in identifying eligible patients, submission of study related materials, Oncore database training, and study related data input.

10.4.3. Patient enrollment

At the time that an eligible patient is identified, the treating physician or his staff will contact the designated study nurse at the coordinating center so that preparations can be made for the transfer of study related materials.

The specific procedures for patient enrollment are as follows:

- Obtain written informed consent from the patient.
- Complete the Eligibility Checklist.
- Complete Patient Enrollment Form and phone 615-936-5795. Ask to speak to the GI research nurse. Fax the Enrollment Form, the first page and the signature page of the informed consent, and the Eligibility Checklist that has been signed by the physician to the research nurse, at 615-936-5794.
- The Patient Enrollment Form will be returned to the patient’s site of treatment by fax with the sequence #.

10.4.4. Sample shipment
At the time of informed consent, the patient will be provided with a unique identification number that will be coded to include the patient's site of treatment as well as their sequence of enrollment on study. A request will also

be made for a paraffin block of diagnostic tissue to be shipped from the patient's treatment location to the coordinating center (VICC). The patient will then have two tubes of whole blood collected into purple top tubes (EDTA vacutainer tubes) and the tubes will be packaged in the provided shipping materials and shipped at ambient temperature along with study-related documentation including their treating physician, their unique study identification number as well as the date of sample acquisition (see Appendix C). The paraffin block may be shipped to Vanderbilt separately at a later date.

To expedite sample processing, one tube of blood for *TSER* assessment will be shipped directly to Washington University. The other tube of blood for other genetic analyses will be shipped to the coordinating center, VICC. Within five working days the results of the patient's *TSER* status will be entered into the Oncore database and a hard copy will be kept at the coordinating center.

10.4.5. Notification of *TSER* genotyping results and treatment assignment

Consultation regarding the patient's eligibility and the results of the *TSER* genetic testing will be reviewed by the principal investigator. If all documentation is in order and *TSER* status is appropriate for study participation, the study nurse will then contact the treating physician's office by telephone and by fax to relay the results of the genetic testing as well as the designated treatment assignment to the treating physician.

10.4.6. Study schedule and data management

- A study folder file and CRFs will be generated in the Oncore database where patient information will be stored. This file will be initiated via the coordination site in a combined effort between the study nurse and the study designated data manager. A study calendar will also be generated documenting when patient visits should occur in compliance with the study protocol, especially visits that involve disease assessments.
- At the time of each patient visit, toxicity and treatment related data will be entered into the Oncore database in the patient's CRF.
- At regular intervals, the database will be reviewed for the completeness of entered material. Incomplete entries will generate a database query that will be followed up on by the designated data manager.

10.4.7. Study monitoring

The designated VICC study monitor will also perform routine monitoring to ensure the enrollment of eligible patients, the appropriate entry of data in the proper receipt of study related materials. Monthly reports regarding patient participation, treatment related toxicities and treatment responses will be reviewed by the study investigators.

11. STATISTICAL CONSIDERATIONS

11.1. Study Design/Endpoints

Multi-centered phase II clinical trial assessing the utility of germline *TSER* polymorphism as a treatment selection marker for 5-FU containing chemotherapy in patients with metastatic gastric and GEJ adenocarcinomas

TSER Status

*2/*3 or *2/*2 → FOLFOX (5-FU, leucovorin, oxaliplatin)
*3/*3 → Not included in study

11.1.1. Primary endpoints

- overall response rate (CR+PR)

11.1.2. Secondary endpoints

- contribution of loss of heterozygosity in tumor *TSER* alleles to the observed 5-FU response rate
- contribution of other genetic polymorphisms (e.g. *TYMS, DPYD, ERCC1, ERCC2, XRCC1* and *GSTP1*) to the response/toxicity in the treated patients

11.2. Sample Size

The primary objective of this study is to improve upon the previously demonstrated treatment responses by selecting patients according to *TSER* genotypes. Sample size estimation is based on the assumed response rate of 43% with this regimen in an unselected population and improving the response rate to a minimum of 60%. This 60% expected response rate is based on the retrospective study data in patients with low tumor TS expression [44, 82] and the fact that the patients who would be resistant to 5-FU treatment will be removed from the study by pretreatment genotyping.

11.2.1. Sample Size Estimation and Statistical Power Analysis

We will determine the response rate (CR + PR) of FOLFOX-6 in these genotypically selected patients. An Optimum MinMax two-stage accrual design described by Simon [66] will ensure that the total number of the patients exposed to this therapy is minimized. If there is evidence that the true underlying overall response rate (CR, PR) is at least 60% in these patients, then consideration for genotype-based treatment selection in a randomized study will be justified. However, if the response rate for FOLFOX-6 is lower than 60% in these patients, then the study should be terminated early. Initially, 45 eligible patients will be entered into the study. If there are fewer than twenty responses in these first 45 patients, the trial will be terminated with the conclusion that there is little evidence to suggest that the overall response rate would reach 60%. If there are 20 or more responses in these first 45 patients, the trial will continue until 75 patients have been treated. If there are 40 or more responses in these 75 patients, then the study will be completed and a randomized study by marker status will be recommended using a similar prospective approach. If there are fewer than 40 responses in these 75 patients, then prospectively genotyping the patients for their TSER status may not be an optimal approach to select treatment for these patients. We will examine potential confounders using the collected tumor and germline genotype data. This design provides 90% statistical power to detect a difference of 17% (60% vs. 43% in an unselected population) with a significance level less than 0.05 (type I error).

11.2.2. Other Statistical Analysis Plan

Demographic information such as age and race will be tabulated. Descriptive statistics, including means, standard deviations and ranges for continuous parameters, as well as percents and frequencies for categorical parameters, will be presented. Adverse medical events will be tabulated. NCI toxicity Grade 3 and Grade 4 laboratory abnormalities will be listed.

The effectiveness of the study treatment will be assessed by response rates. The exact two-sided 95% confidence intervals for the overall response rate will be reported. For lifetime data analyses, e.g., overall and progression-free survival, the possible risk factors will be compared for survival with Kaplan-Meier estimates and log-rank tests. The proportional hazard model will be used for adjusted tests of significance and estimates of odds ratios. The generalized linear model, e.g., logistic regression, will be applied to study the association between the response status and other possible contributing variables including age, performance status, gender, tumor location, prior therapy and treatment location (study site). The adjusted p-values as well as the adjusted 95% confidence intervals will be reported.

11.3. Patient accrual

We anticipate being able to complete the proposed study enrollment (total ~~69~~ 40 patients) over ~~2 years~~45 months. The participating sites will be Vanderbilt University Medical Center, Washington University Medical Center, the University of Alabama at Birmingham and University of North Carolina. Annually, these sites combined see approximately 65 patients with gastric and GEJ cancers. Additionally the VICC website lists on-going clinical trials so that non-participating sites are aware of the currently enrolling studies. This may also assist additional patient accrual.

11.4. Patient Selection

Patients who are with “favorable TSER genotypes (*TSER**2/*2 or *2/*3)” will be selected by pre-treatment genotyping and treated with the FOLFOX regimen.

11.5. Analysis of Secondary Endpoints

All secondary analyses are included to account for potentially unexpected results in the treatment cohorts or in specific patients whose treatment outcome (response or toxicity) deviates substantially from the other observations. The study has not been powered to evaluate these variables, therefore analyses will be considered exploratory.

11.5.1. Loss of tumor *TSER* heterozygosity

Power Analysis and Sample Size Estimation: The focus of the sample size estimation for this secondary objective is to compare the response rates of the sub-group of *3/loss vs. sub-group of <*2/loss + *2/*3>. The response rates and frequency estimates were obtained from a retrospective study by Uchida et al. [76]. Table 6 shows the estimated frequency and response rate for each sub-group based on the study sample size of 50 (it is estimated that 2/3 of the 75 study patients will be in the sub-group of *2/*3).

TABLE 6. Estimated frequency and response rate for tumor TSER genotype subgroups

Germline genotype	*2/*3(n= 50)		
Tumor genotype	*2/loss	*2/*3	*3/loss
Estimated frequency	46%	22%	32%
Estimated # of patients	23	11	16
Estimated response rate	80%	40%	20%
Estimated response rate	65%		20%

The sample size estimation is completed using the Fisher's exact test. With a sample size of 50 (*2/loss=23, *2/*3=11, and *3/loss=16), it provides at least 80% power to detect a 45% difference of the response rate between *3/loss and <*2/loss + *2/*3> with two sided type I error = 5%.

In addition to the statistical analysis plan described in the primary analysis, the Fisher's exact test and the logistic regression model will be applied to study the association between the response rate and the study groups, i.e., *3/loss vs. <*2/loss + *2/*3>.

11.5.2. Tumor TS mRNA and protein expression

Descriptive statistics, including means, standard deviations and ranges for continuous parameters (TS mRNA expression levels), as well as percents and frequencies for categorical parameters (TS protein expression levels) will be obtained for responder and non-responder groups to the study treatment. The Wilcoxon test statistic will be used for testing continuous outcomes (TS mRNA expression levels) and ordinal outcomes (TS protein levels) between the responders and non-responders. Assuming that our estimated responder and non-responder proportions are correct (e.g., 60% responders to FOLFOX-6), the Wilcoxon test statistic will have 80% power to detect distributional shifts for the TS mRNA expression levels within these groups if the distributional shift (probability of the distribution of one group being larger or smaller than the other) is 0.67 or greater. We will have 80% power to detect proportional odds ratios between the ordinal levels of the TS protein variables of at least 3.8. To look at the relationship between tumor TS mRNA and protein expression, we will use scatterplots and simple linear regression analysis (if possible with sample distribution). We will have 85% power to detect correlations (slope parameters) of at least 0.5 between TS mRNA and protein levels.

11.5.3. Polymorphisms in other genes associated with treatment outcomes or toxicity

The sample size estimation is completed using the Fisher's exact test. For each polymorphism considered for its potential contribution to response, the statistical analysis is based upon whether the presence of the polymorphic allele affects the response rate. To improve the power of the analysis, the response rate will be estimated in the subgroup with the polymorphic alleles (combining heterozygous and homozygous) and the subgroup without the polymorphic alleles. With a sample size of 75, it provides at least 80% power to detect a 40% difference of the response rate between the two subgroups with two-sided type I error = 5%. A difference in the response rate of 40% is readily detectable if such a relationship exists. Polymorphisms demonstrating a trend toward significance in the secondary analysis can be further explored in a Phase III study. Since the focus of this aim is to estimate the response rate, the multiple comparisons adjustment is not applied to the sample size estimation. Table below shows the detailed results of the power analyses.

Table 7. Sample size estimation for the difference of 40% response rate between two groups

N=75		Estimated frequency	Expected sample size for each group	Power
Gene ID	SNP			
TYMS (7298)	G>C (within the 2nd tandem repeat)	0.25	19 vs. 56	> 80%

TYMS (7298)	6 bp deletion	0.29	22 vs. 53	> 85%
ERCC1 (2067)	C354T	0.3	23 vs. 52	> 85%
ERCC1 (2067)	C8092A	0.3	23 vs. 52	> 85%
ERCC2 (2068)	A2251C	0.3	23 vs. 52	> 85%
XRCC1 (7515)	G1196A	0.37	28 vs. 47	> 90%
GSTP1 (2950)	A313G	0.3	23 vs. 52	> 85%

In addition, each polymorphism will be tested whether it deviates from Hardy-Weinberg equilibrium. Haplotypes will be analyzed using the haplo.stats package, which implements the expectation maximization algorithm to estimate haplotype frequencies.

11.6. ***Reporting and Exclusions***

11.6.1. Evaluation of toxicity

All patients will be evaluable for toxicity from the time of their first treatment with chemotherapy.

11.6.2. Evaluation of response

All patients included in the study must be assessed for response to treatment, even if there are major protocol treatment deviations or if they are ineligible. Each patient will be assigned one of the following categories: 1) complete response, 2) partial response, 3) stable disease, 4) progressive disease, 5) early death from malignant disease, 6) early death from toxicity, 7) early death because of other cause, or 9) unknown (not assessable, insufficient data, early withdrawals, early discontinuation of treatment due to toxicities, etc.). [Note: By arbitrary convention, category 9 usually designates the “unknown” status of any type of data in a clinical database.

All of the patients who met the eligibility criteria (with the possible exception of those who received no study medications) should be included in the main analysis of the response rate. Patients in response categories 4-9 should be considered as failing to respond to treatment (disease progression). Thus, an incorrect treatment schedule or drug administration does not result in exclusion from the analysis of the response rate.

REFERENCES

1. ACS. 2005. Cancer Facts and Figures. American Cancer Society.
2. Ajani, J. A., J. Baker, P. W. Pisters, L. Ho, P. F. Mansfield, B. W. Feig, and C. Charnsangavej. 2002. Irinotecan/cisplatin in advanced, treated gastric or gastroesophageal junction carcinoma. Oncology (Huntington) **16:**16-8.
3. Ajani, J. A., E. Van Custem, and F. C. Moiseyenko. 2003. Docetaxel (D), cisplatin, 5-fluorouracil compared to cisplatin (C) and 5-fluorouracil (F) for chemotherapy-naive patients with metastatic or locally recurrent, unresectable gastric carcinoma: Interim results of a randomized phase III trial (V 325). Proceedings of the American Society of Clinical Oncology **22:**999 (abstract).
4. Al-Batran, S. E., A. Atmaca, S. Hegewisch-Becker, D. Jaeger, S. Hahnfeld, M. J. Rummel, G. Seipelt, A. Rost, J. Orth, A. Knuth, and E. Jaeger. 2004. Phase II trial of biweekly infusional fluorouracil, folinic acid, and oxaliplatin in patients with advanced gastric cancer. Journal of Clinical Oncology **22:**658-63.
5. Ando, Y., H. Saka, M. Ando, T. Sawa, K. Muro, H. Ueoka, A. Yokoyama, S. Saitoh, K. Shimokata, and Y. Hasegawa. 2000. Polymorphisms of UDP-glucuronosyltransferase gene and irinotecan toxicity: a pharmacogenetic analysis. Cancer Research **60:**6921-6.
6. Andre, T., A. Figer, A. Cervantes, G. Lledo, M. Flesch, F. Maindrault-Goebel, L. Mineur, G. Russ, M. Buyse, and A. De Gramont. 2003. FOLFOX7 compared to FOLFOX4. Preliminary results of the randomized optimox study. . Proceedings of the American Society of Clinical Oncology **22:**253 (abstract 1016).
7. Arnould, S., I. Hennebelle, P. Canal, R. Bugat, and S. Guichard. 2003. Cellular determinants of oxaliplatin sensitivity in colon cancer cell lines. European Journal of Cancer **39:**112-9.
8. Blot, W. J., S. S. Devesa, R. W. Kneller, and J. F. Fraumeni, Jr. 1991. Rising incidence of adenocarcinoma of the esophagus and gastric cardia.[see comment]. JAMA **265:**1287-9.
9. Boku, N., A. Ohtsu, Y. Shimada, K. Shirao, S. Seki, H. Saito, Y. Sakata, and I. Hyodo. 1999. Phase II study of a combination of irinotecan and cisplatin against metastatic gastric cancer. Journal of Clinical Oncology **17:**319-23.
10. Broughton, B. C., H. Steingrimsdottir, and A. R. Lehmann. 1996. Five polymorphisms in the coding sequence of the xeroderma pigmentosum group D gene. Mutation Research **362:**209-11.
11. Chu, E., A. C. Moto, and M. C. Fogarasi. 2001. Cancer: Principles & Practice of Oncology, 6th ed. Lippincott Williams & Wilkins, Philadelphia.
12. Collie-Duguid, E. S., M. C. Etienne, G. Milano, and H. L. McLeod. 2000. Known variant DPYD alleles do not explain DPD deficiency in cancer patients. Pharmacogenetics **10:**217-23.
13. Crew, K. D., and A. I. Neugut. 2004. Epidemiology of upper gastrointestinal malignancies. Seminars in Oncology **31:**450-64.
14. Dabholkar, M., J. Vionnet, F. Bostick-Bruton, J. J. Yu, and E. Reed. 1994. Messenger RNA levels of XPAC and ERCC1 in ovarian cancer tissue correlate with response to platinum-based chemotherapy. Journal of Clinical Investigation **94:**703-8.
15. Dank, M., J. Zaluski, C. Barone, V. Valvere, C. Peschel, M. Wenczl, E. Goker, M. Risse, L. Awad, and R. Bugat. 2005. Randomized phase 3 trial of irinotecan (CPT-11) + 5FU/folinic acid (FA) vs CDDP + 5FU in 1st-line advanced gastric cancer patients. Proceedings of the American Society of Clinical Oncology **24:**4003 (abstract).
16. De Vita, F., M. Orditura, E. Matano, R. Bianco, C. Carlomagno, S. Infusino, V. Damiano, E. Simeone, M. R. Diadema, E. Lieto, P. Castellano, S. Pepe, S. De Placido, G. Galizia, N. Di Martino, F. Ciardiello, G. Catalano, and A. R. Bianco. 2005. A phase II study of biweekly oxaliplatin plus infusional 5-fluorouracil and folinic acid (FOLFOX-4) as first-line treatment of advanced gastric cancer patients. British Journal of Cancer **92:**1644-9.
17. Evans, W. E., M. Horner, Y. Q. Chu, D. Kalwinsky, and W. M. Roberts. 1991. Altered

mercaptopurine metabolism, toxic effects, and dosage requirement in a thiopurine methyltransferase-deficient child with acute lymphocytic leukemia. Journal of Pediatrics **119:**985-9.
18. Glimelius, B., K. Ekstrom, K. Hoffman, W. Graf, P. O. Sjoden, U. Haglund, C. Svensson, L. K. Enander, T. Linne, H. Sellstrom, and R. Heuman. 1997. Randomized comparison between chemotherapy plus best supportive care with best supportive care in advanced gastric cancer. Annals of Oncology **8:**163-8.
19. Goode, E. L., C. M. Ulrich, and J. D. Potter. 2002. Polymorphisms in DNA repair genes and associations with cancer risk.[erratum appears in Cancer Epidemiol Biomarkers Prev. 2003 Oct;12(10):1119]. Cancer Epidemiology, Biomarkers & Prevention **11:**1513-30.
20. Goto, S., T. Iida, S. Cho, M. Oka, S. Kohno, and T. Kondo. 1999. Overexpression of glutathione S-transferase pi enhances the adduct formation of cisplatin with glutathione in human cancer cells. Free Radical Research **31:**549-58.
21. Grothey, A., and R. M. Goldberg. 2004. A review of oxaliplatin and its clinical use in colorectal cancer. Expert Opinion on Pharmacotherapy **5:**2159-70.
22. Horie, N., H. Aiba, K. Oguro, H. Hojo, and K. Takeishi. 1995. Functional analysis and DNA polymorphism of the tandemly repeated sequences in the 5'-terminal regulatory region of the human gene for thymidylate synthase. Cell Structure & Function **20:**191-7.
23. Iacopetta, B., F. Grieu, D. Joseph, and H. Elsaleh. 2001. A polymorphism in the enhancer region of the thymidylate synthase promoter influences the survival of colorectal cancer patients treated with 5-fluorouracil.[see comment]. British Journal of Cancer **85:**827-30.
24. Ishida, Y., K. Kawakami, Y. Tanaka, E. Kanehira, K. Omura, and G. Watanabe. 2002. Association of thymidylate synthase gene polymorphism with its mRNA and protein expression and with prognosis in gastric cancer. Anticancer Research **22:**2805-9.
25. Iyer, L., S. Das, L. Janisch, M. Wen, J. Ramirez, T. Karrison, G. F. Fleming, E. E. Vokes, R. L. Schilsky, and M. J. Ratain. 2002. UGT1A1*28 polymorphism as a determinant of irinotecan disposition and toxicity. Pharmacogenomics Journal **2:**43-7.
26. Janunger, K. G., L. Hafstrom, and B. Glimelius. 2002. Chemotherapy in gastric cancer: a review and updated meta-analysis. European Journal of Surgery **168:**597-608.
27. Kawakami, K., K. Omura, E. Kanehira, and Y. Watanabe. 1999. Polymorphic tandem repeats in the thymidylate synthase gene is associated with its protein expression in human gastrointestinal cancers. Anticancer Research **19:**3249-52.
28. Kawakami, K., D. Salonga, J. M. Park, K. D. Danenberg, H. Uetake, J. Brabender, K. Omura, G. Watanabe, and P. V. Danenberg. 2001. Different lengths of a polymorphic repeat sequence in the thymidylate synthase gene affect translational efficiency but not its gene expression. Clinical Cancer Research **7:**4096-101.
29. Kawakami, K., and G. Watanabe. 2003. Identification and functional analysis of single nucleotide polymorphism in the tandem repeat sequence of thymidylate synthase gene. Cancer Research **63:**6004-7.
30. Kawate, H., D. M. Landis, and L. A. Loeb. 2002. Distribution of mutations in human thymidylate synthase yielding resistance to 5-fluorodeoxyuridine. Journal of Biological Chemistry **277:**36304-11.
31. Kelley, J. R., and J. M. Duggan. 2003. Gastric cancer epidemiology and risk factors. Journal of Clinical Epidemiology **56:**1-9.
32. Kim, D. Y., J. H. Kim, S. H. Lee, T. Y. Kim, D. S. Heo, Y. J. Bang, and N. K. Kim. 2003. Phase II study of oxaliplatin, 5-fluorouracil and leucovorin in previously platinum-treated patients with advanced gastric cancer. Annals of Oncology **14:**383-7.
33. Kim, Y. H., S. W. Shin, B. S. Kim, J. H. Kim, J. G. Kim, Y. J. Mok, C. S. Kim, H. S. Rhyu, J. H. Hyun, and J. S. Kim. 1999. Paclitaxel, 5-fluorouracil, and cisplatin combination chemotherapy for the treatment of advanced gastric carcinoma. Cancer **85:**295-301.
34. Kollmannsberger, C., D. Quietzsch, C. Haag, T. Lingenfelser, M. Schroeder, J. T. Hartmann, W. Baronius, V. Hempel, M. Clemens, L. Kanz, and C. Bokemeyer. 2000. A phase II study of

paclitaxel, weekly, 24-hour continous infusion 5-fluorouracil, folinic acid and cisplatin in patients with advanced gastric cancer. British Journal of Cancer **83:**458-62.
35. Lenz, H. J. 2004. Pharmacogenomics and colorectal cancer. Annals of Oncology **15 Suppl 4:**iv173-7.
36. Lim, W. T., S. T. Lim, N. S. Wong, and W. H. Koo. 2003. CPT-11 and cisplatin in the treatment of advanced gastric cancer in Asians. Journal of Chemotherapy **15:**400-5.
37. Louvet, C., T. Andre, J. M. Tigaud, E. Gamelin, J. Y. Douillard, R. Brunet, E. Francois, J. H. Jacob, D. Levoir, A. Taamma, P. Rougier, E. Cvitkovic, and A. de Gramont. 2002. Phase II study of oxaliplatin, fluorouracil, and folinic acid in locally advanced or metastatic gastric cancer patients. Journal of Clinical Oncology **20:**4543-8.
38. Lu, Z., R. Zhang, and R. B. Diasio. 1993. Dihydropyrimidine dehydrogenase activity in human peripheral blood mononuclear cells and liver: population characteristics, newly identified deficient patients, and clinical implication in 5-fluorouracil chemotherapy. Cancer Research **53:**5433-8.
39. Maindrault-Goebel, F., A. de Gramont, C. Louvet, T. Andre, E. Carola, V. Gilles, J. P. Lotz, C. Tournigand, M. Mabro, J. L. Molitor, P. Artru, V. Izrael, and M. Krulik. 2000. Evaluation of oxaliplatin dose intensity in bimonthly leucovorin and 48-hour 5-fluorouracil continuous infusion regimens (FOLFOX) in pretreated metastatic colorectal cancer. Oncology Multidisciplinary Research Group (GERCOR). Annals of Oncology **11:**1477-83.
40. Maindrault-Goebel, F., C. Louvet, T. Andre, E. Carola, J. P. Lotz, J. L. Molitor, M. L. Garcia, V. Gilles-Amar, V. Izrael, M. Krulik, and A. de Gramont. 1999. Oxaliplatin added to the simplified bimonthly leucovorin and 5-fluorouracil regimen as second-line therapy for metastatic colorectal cancer (FOLFOX6). GERCOR. European Journal of Cancer **35:**1338-42.
41. Mandola, M. V., J. Stoehlmacher, S. Muller-Weeks, G. Cesarone, M. C. Yu, H. J. Lenz, and R. D. Ladner. 2003. A novel single nucleotide polymorphism within the 5' tandem repeat polymorphism of the thymidylate synthase gene abolishes USF-1 binding and alters transcriptional activity. Cancer Research **63:**2898-904.
42. Marsh, S., E. S. Collie-Duguid, T. Li, X. Liu, and H. L. McLeod. 1999. Ethnic variation in the thymidylate synthase enhancer region polymorphism among Caucasian and Asian populations. Genomics **58:**310-2.
43. Marsh, S., and H. L. McLeod. 2001. Thymidylate synthase pharmacogenetics in colorectal cancer.[see comment]. Clinical Colorectal Cancer **1:**175-8; discussion 179-81.
44. Metzger, R., C. G. Leichman, K. D. Danenberg, P. V. Danenberg, H. J. Lenz, K. Hayashi, S. Groshen, D. Salonga, H. Cohen, L. Laine, P. Crookes, H. Silberman, J. Baranda, B. Konda, and L. Leichman. 1998. ERCC1 mRNA levels complement thymidylate synthase mRNA levels in predicting response and survival for gastric cancer patients receiving combination cisplatin and fluorouracil chemotherapy. Journal of Clinical Oncology **16:**309-16.
45. Milano, G., M. C. Etienne, V. Pierrefite, M. Barberi-Heyob, R. Deporte-Fety, and N. Renee. 1999. Dihydropyrimidine dehydrogenase deficiency and fluorouracil-related toxicity. British Journal of Cancer **79:**627-30.
46. Moscow, J. A., C. R. Fairchild, M. J. Madden, D. T. Ransom, H. S. Wieand, E. E. O'Brien, D. G. Poplack, J. Cossman, C. E. Myers, and K. H. Cowan. 1989. Expression of anionic glutathione-S-transferase and P-glycoprotein genes in human tissues and tumors. Cancer Research **49:**1422-8.
47. Murad, A. M., F. F. Santiago, A. Petroianu, P. R. Rocha, M. A. Rodrigues, and M. Rausch. 1993. Modified therapy with 5-fluorouracil, doxorubicin, and methotrexate in advanced gastric cancer. Cancer **72:**37-41.
48. Nakamura, K., T. Ueyama, T. Yao, Z. X. Xuan, K. Ambe, Y. Adachi, Y. Yakeishi, A. Matsukuma, and M. Enjoji. 1992. Pathology and prognosis of gastric carcinoma. Findings in 10,000 patients who underwent primary gastrectomy. Cancer **70:**1030-7.
49. Ohtsu, A., Y. Shimada, K. Shirao, N. Boku, I. Hyodo, H. Saito, N. Yamamichi, Y. Miyata, N.

Ikeda, S. Yamamoto, H. Fukuda, S. Yoshida, and S. Japan Clinical Oncology Group. 2003. Randomized phase III trial of fluorouracil alone versus fluorouracil plus cisplatin versus uracil and tegafur plus mitomycin in patients with unresectable, advanced gastric cancer: The Japan Clinical Oncology Group Study (JCOG9205). Journal of Clinical Oncology **21:**54-9.

50. Park, D., J. Stoehlmacher, W. Zhang, D. Tsao-Wei, S. Groshen, S. Zahedy, J. Gil, N. Mallik, and H. J. Lenz. 2002. ERCC1 polymorphism is associated with differential ERCC1 mRNA levels. Proceedings of the American Association for Cancer Research **43:**Abstract 1591.
51. Park, D. J., J. Stoehlmacher, W. Zhang, D. D. Tsao-Wei, S. Groshen, and H. J. Lenz. 2001. A Xeroderma pigmentosum group D gene polymorphism predicts clinical outcome to platinum-based chemotherapy in patients with advanced colorectal cancer. Cancer Research **61:**8654-8.
52. Park, D. J., W. Zhang, J. Stoehlmacher, D. Tsao-Wei, S. Groshen, J. Gil, J. Yun, E. Sones, N. Mallik, and H. J. Lenz. 2003. ERCC1 gene polymorphism as a predictor for clinical outcome in advanced colorectal cancer patients treated with platinum-based chemotherapy. Clinical Advances in Hematology and Oncology **1:**162-6.
53. Pozzo, C., R. Bugat, and C. Peschel. 2001. Irinotecan in combination with CDDP or 5-FU and folinic acid is active in patients with advanced gastric or gastro-oesophageal junction adenocarcinoma: Final results of a randomized phase II study. Proceedings of the American Society of Clinical Oncology **21:**531a (abstract).
54. Pullarkat, S. T., J. Stoehlmacher, V. Ghaderi, Y. P. Xiong, S. A. Ingles, A. Sherrod, R. Warren, D. Tsao-Wei, S. Groshen, and H. J. Lenz. 2001. Thymidylate synthase gene polymorphism determines response and toxicity of 5-FU chemotherapy. Pharmacogenomics Journal **1:**65-70.
55. Pyrhonen, S., T. Kuitunen, P. Nyandoto, and M. Kouri. 1995. Randomised comparison of fluorouracil, epidoxorubicin and methotrexate (FEMTX) plus supportive care with supportive care alone in patients with non-resectable gastric cancer. British Journal of Cancer **71:**587-91.
56. Raymond, E., S. Faivre, S. Chaney, J. Woynarowski, and E. Cvitkovic. 2002. Cellular and molecular pharmacology of oxaliplatin. Molecular Cancer Therapeutics **1:**227-35.
57. Relling, M. V., and T. Dervieux. 2001. Pharmacogenetics and cancer therapy. Nature Reviews Cancer **1:**99-108.
58. Ridwelski, K., T. Gebauer, J. Fahlke, H. Kroning, E. Kettner, F. Meyer, K. Eichelmann, and H. Lippert. 2001. Combination chemotherapy with docetaxel and cisplatin for locally advanced and metastatic gastric cancer. Annals of Oncology **12:**47-51.
59. Ross, P., M. Nicolson, D. Cunningham, J. Valle, M. Seymour, P. Harper, T. Price, H. Anderson, T. Iveson, T. Hickish, F. Lofts, and A. Norman. 2002. Prospective randomized trial comparing mitomycin, cisplatin, and protracted venous-infusion fluorouracil (PVI 5-FU) With epirubicin, cisplatin, and PVI 5-FU in advanced esophagogastric cancer.[see comment]. Journal of Clinical Oncology **20:**1996-2004.
60. Roth, A. D., R. Maibach, G. Martinelli, N. Fazio, M. S. Aapro, O. Pagani, R. Morant, M. M. Borner, R. Herrmann, H. Honegger, F. Cavalli, P. Alberto, M. Castiglione, and A. Goldhirsch. 2000. Docetaxel (Taxotere)-cisplatin (TC): an effective drug combination in gastric carcinoma. Swiss Group for Clinical Cancer Research (SAKK), and the European Institute of Oncology (EIO). Annals of Oncology **11:**301-6.
61. Shah, M. A., and G. K. Schwartz. 2004. Treatment of metastatic esophagus and gastric cancer. Seminars in Oncology **31:**574-87.
62. Shen, M. R., I. M. Jones, and H. Mohrenweiser. 1998. Nonconservative amino acid substitution variants exist at polymorphic frequency in DNA repair genes in healthy humans. Cancer Research **58:**604-8.
63. Shirota, Y., J. Stoehlmacher, J. Brabender, Y. P. Xiong, H. Uetake, K. D. Danenberg, S. Groshen, D. D. Tsao-Wei, P. V. Danenberg, and H. J. Lenz. 2001. ERCC1 and thymidylate synthase mRNA levels predict survival for colorectal cancer patients receiving combination

oxaliplatin and fluorouracil chemotherapy. Journal of Clinical Oncology **19:**4298-304.
64. Siciliano, M. J., A. V. Carrano, and L. H. Thompson. 1986. Assignment of a human DNA-repair gene associated with sister-chromatid exchange to chromosome 19. Mutation Research **174:**303-8.
65. Siewert, J. R., K. Bottcher, H. J. Stein, and J. D. Roder. 1998. Relevant prognostic factors in gastric cancer: ten-year results of the German Gastric Cancer Study.[see comment]. Annals of Surgery **228:**449-61.
66. Simon, R. 1989. Optimal two-stage designs for phase II clinical trials. Control Clin Trials **10:**1-10.
67. Souglakos, J., K. Syrigos, A. Potamianou, A. Polyzos, I. Boukovinas, N. Androulakis, C. Kouroussis, N. Vardakis, C. Christophilakis, A. Kotsakis, and V. Georgoulias. 2004. Combination of irinotecan (CPT-11) plus oxaliplatin (L-OHP) as first-line treatment in locally advanced or metastatic gastric cancer: a multicentre phase II trial. Annals of Oncology **15:**1204-9.
68. Spitz, M. R., X. Wu, Y. Wang, L. E. Wang, S. Shete, C. I. Amos, Z. Guo, L. Lei, H. Mohrenweiser, and Q. Wei. 2001. Modulation of nucleotide excision repair capacity by XPD polymorphisms in lung cancer patients. Cancer Research **61:**1354-7.
69. Stoehlmacher, J., V. Ghaderi, S. Iobal, S. Groshen, D. Tsao-Wei, D. Park, and H. J. Lenz. 2001. A polymorphism of the XRCC1 gene predicts for response to platinum based treatment in advanced colorectal cancer. Anticancer Research **21:**3075-9.
70. Stoehlmacher, J., and H. J. Lenz. 2003. Implications of genetic testing in the management of colorectal cancer. American Journal of PharmacoGenomics **3:**73-88.
71. Stoehlmacher, J., D. J. Park, W. Zhang, S. Groshen, D. D. Tsao-Wei, M. C. Yu, and H. J. Lenz. 2002. Association between glutathione S-transferase P1, T1, and M1 genetic polymorphism and survival of patients with metastatic colorectal cancer. Journal of the National Cancer Institute **94:**936-42.
72. Tan, B. R., and H. L. McLeod. 2005. Pharmacogenetic influences on treatment response and toxicity in colorectal cancer. Seminars in Oncology **32:**113-9.
73. Therasse, P., S. G. Arbuck, E. A. Eisenhauer, J. Wanders, R. S. Kaplan, L. Rubinstein, J. Verweij, M. Van Glabbeke, A. T. van Oosterom, M. C. Christian, and S. G. Gwyther. 2000. New guidelines to evaluate the response to treatment in solid tumors. European Organization for Research and Treatment of Cancer, National Cancer Institute of the United States, National Cancer Institute of Canada. J Natl Cancer Inst **92:**205-16.
74. Thompson, L. H., K. W. Brookman, N. J. Jones, S. A. Allen, and A. V. Carrano. 1990. Molecular cloning of the human XRCC1 gene, which corrects defective DNA strand break repair and sister chromatid exchange. Molecular & Cellular Biology **10:**6160-71.
75. Tournigand, C., A. d Gramont, C. Louvet, T. Andre, E. Carola, V. Gilles-Amar, F. Maindrault-Goebel, J. P. Lotz, J. L. Molitor, V. Izrael, E. Ecstein-Fraisse, and M. Krulik. 1998. A simplified bimonthly regimen with leucovorin and 5 FU for metastatic colorectal cancer. Proceedings of the American Society of Clinical Oncology **17:**274 (abstract 1052).
76. Uchida, K., K. Hayashi, K. Kawakami, S. Schneider, J. M. Yochim, H. Kuramochi, K. Takasaki, K. D. Danenberg, and P. V. Danenberg. 2004. Loss of heterozygosity at the thymidylate synthase (TS) locus on chromosome 18 affects tumor response and survival in individuals heterozygous for a 28-bp polymorphism in the TS gene. Clin Cancer Res **10:**433-9.
77. Ulrich, C. M., J. Bigler, C. M. Velicer, E. A. Greene, F. M. Farin, and J. D. Potter. 2000. Searching expressed sequence tag databases: discovery and confirmation of a common polymorphism in the thymidylate synthase gene. Cancer Epidemiology, Biomarkers & Prevention **9:**1381-5.
78. Villafranca, E., Y. Okruzhnov, M. A. Dominguez, J. Garcia-Foncillas, I. Azinovic, E. Martinez, J. J. Illarramendi, F. Arias, R. Martinez Monge, E. Salgado, S. Angeletti, and A. Brugarolas. 2001. Polymorphisms of the repeated sequences in the enhancer region of the thymidylate

synthase gene promoter may predict downstaging after preoperative chemoradiation in rectal cancer.[see comment]. Journal of Clinical Oncology **19:**1779-86.

79. Watson, M. A., R. K. Stewart, G. B. Smith, T. E. Massey, and D. A. Bell. 1998. Human glutathione S-transferase P1 polymorphisms: relationship to lung tissue enzyme activity and population frequency distribution. Carcinogenesis **19:**275-80.
80. Webb, A., D. Cunningham, J. H. Scarffe, P. Harper, A. Norman, J. K. Joffe, M. Hughes, J. Mansi, M. Findlay, A. Hill, J. Oates, M. Nicolson, T. Hickish, M. O'Brien, T. Iveson, M. Watson, C. Underhill, A. Wardley, and M. Meehan. 1997. Randomized trial comparing epirubicin, cisplatin, and fluorouracil versus fluorouracil, doxorubicin, and methotrexate in advanced esophagogastric cancer.[see comment]. Journal of Clinical Oncology **15:**261-7.
81. Wilson, M. D., C. C. Ruttan, B. F. Koop, and B. W. Glickman. 2001. ERCC1: a comparative genomic perspective. Environmental & Molecular Mutagenesis **38:**209-15.
82. Yeh, K. H., C. T. Shun, C. L. Chen, J. T. Lin, W. J. Lee, P. H. Lee, Y. C. Chen, and A. L. Cheng. 1998. High expression of thymidylate synthase is associated with the drug resistance of gastric carcinoma to high dose 5-fluorouracil-based systemic chemotherapy. Cancer **82:**1626-31.

PROPOSED INFORMED CONSENT

This informed consent applies to: **Adults**

Name of participant: ____
Age: ___________

The following is given to you to tell you about this research study. Please read this form with care and ask any questions you may have about this study. Your questions will be answered. Also, you will be given a copy of this consent form.

You do not have to be in this research study. You may choose not to be in this study and get other treatments without changing your healthcare, services or other rights. You can stop being in this study at any time. If we learn something new that may affect the risks or benefits of this study, you will be told so that you can decide whether or not you still want to be in this study.

1. What is the purpose of this study?

You are being asked to take part in this research study because you have stomach cancer or cancer of the lower part of the esophagus that has either spread to other organs. This is a clinical trial (a type of research study). Clinical trials include only patients who choose to take part. Please take your time to make your decision and discuss it with your friends and family.

There are many different chemotherapy treatments for your type of cancer. At the present time, there is no proven way to choose a therapy for an individual patient that is most likely to be of benefit. Some studies have shown that people with a certain genetic difference in an important gene that has to do with how some chemotherapies work may respond differently to chemotherapy than those patients without that genetic difference. We inherit these gene differences from our parents. The genetic difference that we are studying is a difference in the thymidylate synthase or TS gene. We are hoping that by treating patients according to their genes, that they may respond to treatment of their cancer better and it will help us choose cancer treatments better in the future.

In this study, you will be asked to have your blood drawn before you get any treatment. If you have one type of the TS gene (called "star 3-star 3" abbreviated *3/*3), you will not be able to participate in this study. This type of TS gene is seen in about 1 out of every 3 patients. Your doctor will recommend treatment for your cancer outside of this study.

If you have other types of the TS gene (called star 2-star 2 and star 3-star 2 abbreviated *2/*2 and *3/*2), then studies show that you may respond well to the 5-FU and we will recommend a chemotherapy with 5-FU in it. We see these types of TS genes in about two-thirds of patients.

If you have the *2/*2 or the *3/*2 TS gene you will be recommended to get a

chemotherapy treatment called FOLFOX. FOLFOX has three chemotherapy drugs in it and they are 5-FU, leucovorin and oxaliplatin. FOLFOX is an accepted treatment for stomach cancers and cancers of the lower esophagus. Details about the FOLFOX treatment are discussed later in this study form.

What will happen and how long will you be in the study?
You will also have the following tests. Some of these tests would be done even if you did not take part in the study. The tests that are being done for research purposes only will be noted with a check (√).

Before treatment:
√ If you choose to take part in this study, you will have two tubes of blood drawn. The first tube will be sent to a lab at Washington University in St. Louis where they will check to see what kind of TS gene you have. The other tube will be sent to Vanderbilt University in Nashville where other genetic tests will be done. Only the results of test for the TS gene will be used to recommend your chemotherapy treatment. The other genetic tests are to look at other genes that may also affect how you respond to the chemotherapy and the side-effects of the treatment. We will also ask that a sample of your tumor be sent to Vanderbilt University for testing. This testing is also being done to look at genes and proteins in the tumor and how they affect your response to chemotherapy. The tumor sample should already have been taken as part of how they diagnosed you with this cancer, so you should not need to have a tumor biopsy.

Within 5 days we should know the results of your TS gene testing and we will know whether you can receive treatment on this study or whether treatment outside of the study will be recommended for you.

- Female patients will have a pregnancy test.
- You will receive a complete physical exam and be seen by a physician at the beginning of each cycle of treatment.
- Samples of your blood will be drawn, along with chest x-rays, CT scans or MRI to evaluate your health status.
- An electrocardiogram (EKG) will be done.

Treatment:
If you have the TS *2/*2 or *2/*3 gene, you will get treatment with FOLFOX chemotherapy. This is a two-day treatment given every other week. Medicines will be given before chemotherapy to prevent nausea and vomiting. All of the chemotherapy drugs will be given intravenously (through a vein). Oxaliplatin will be given over 120 minutes. Leucovorin will be given at the same time as the oxaliplatin. 5-FU will be given last as a quick infusion, followed by 5-FU given as a continuous infusion through a portable pump over the next 46 hours. The portable pump will be provided for you. Instructions about care of the pump will also be provided.

Prior to treatment, blood will be drawn (every 2 weeks) to check blood counts, liver and kidney function, and a tumor marker called CEA. Based on the side effects you experience and, depending on the results of the blood tests, the doses of the study drugs may be changed to make sure you receive the dose of chemotherapy you can handle.

After every 4 chemotherapy treatments you will have an assessment of your tumor, by either CT scan or MRI. These studies would be done whether you took part in this study or not. If the chemotherapy is keeping your tumor from growing or is causing it to shrink, and you are tolerating the chemotherapy well, you doctor will probably recommend continuing with your treatment. If the cancer appears to be growing or you are not handling the chemotherapy well, then your doctor will recommend stopping the treatment and possibly considering other options for treatment.

We think you will be in the study for at least eight weeks before the status of the tumor is checked to see if the therapy is helping. If the re-staging studies show that the tumor is either shrinking or not growing and you tolerate the therapy, then you can continue on therapy as long as you can handle the side effects of therapy and you wish to continue. If the tumor shows definite growth at any point, then the therapy will be stopped. Even if you stop treatment on the study we would like to keep in touch with you or keep in touch with your doctor about you for 4 years or until you die. Your doctor may decide to take you off this study for the following reasons, even if you wish to stay on the protocol:

- growth of the cancer
- you cannot tolerate treatment
- your doctors feel that the risks of continuing on the protocol therapy are too great
- you are unable to comply with the study guidelines for treatment and follow-up
- you become pregnant or start to breast-feed

You may stop participating at any time. However, we encourage you to talk to your doctor before you decide to withdraw, to explain your reasons and to ask what effect your decision may have on the cancer.

Important information about genetic testing:
The purpose of this study is to look at genes (DNA) and how they affect health and disease. Genes are the instruction manual for your body. The genes you get from your parents decide what you look like and how your body behaves. They can also tell us a person's risk for certain diseases and how they will respond to treatment.

You are being asked to give a blood sample for genetic research. What we learn about you from this sample will not be put in your health record. No one else (like a relative, boss, or insurance company) will be given your test results. Your sample will only be used for research at Vanderbilt University and will not be sold.

Two single blood samples of about a teaspoon each *will be drawn from a vein in your arm using a needle.* This will take about *10 minutes* of your time.

One risk of giving samples for this research may be the release of your name that could link you to the stored samples and/or the results of the tests run on your samples. This may cause problems with insurance or getting a job. To prevent this, these samples will be given a code. Only the study staff will know the code. The name that belongs to the code will be kept in a locked file or in a computer with a password. Only (investigator's name and/or other's names) will have access to your name.

Your sample will be used to make DNA that will be kept for an unknown length of time (maybe years) for future research. The sample will be destroyed when it is no longer needed.

Your samples will be used for research only and will not be sold or used to make products that could be sold for money.

At any time, you may ask to have your sample destroyed. You should contact Dr. Goff or study her staff at 777 Preston Research Bldg. Nashville, TN 37232-6307 – (615) 322-4967 to have your sample destroyed and no longer used for research. We will not be able to destroy research data that has already been gathered using your sample. Also, if your identity was removed from the samples, we will not be able to locate and destroy them.

2. Costs to you if you take part in this study:

Taking part in this study may lead to added costs to you or your insurance company. However, most of the tests that are being done to you as part of the study are considered normal in the routine care of your type of cancer so your insurance should cover payment for them. All tests that are being done for research purposes only will be performed at no cost to you or your insurance company. You may find the National Cancer Institute's guide "Clinical Trials and Insurance Coverage - A Resource Guide" helpful. Ask your doctor for a copy. It is also available on the World Wide Web at: http://www.cancer.gov/clinicaltrials/learning/insurance-coverage

In some instances insurance companies do not pay for the chemotherapy drug oxaliplatin because it is only FDA approved to be given to patients with colon cancer. Please let you doctor know if this happens as we can write a letter on your behalf to the insurance company appealing their decision. If an appeal does not work there may be funds available to pay for the oxaliplatin.

3. Side effects and risks that you can expect if you take part in this study:

While on the study, you are at risk for the following side effects. The drugs used in this study, 5-FU, leucovorin and oxaliplatin may cause some, all or none of the side

effects listed. You should discuss these with your doctor. There may also be other side effects that we cannot predict. Other drugs will be given to make side effects less serious and less uncomfortable. Many side effects go away shortly after the study therapy is stopped, but in some cases side effects can be serious, long-lasting, permanent or life-threatening. Death is rare, but possible.

Your physician will check you closely to see if any of these side effects are occurring and routine blood tests will be done to monitor the effects of treatment.

Risks and side effects related to the drugs and procedures we are studying include:

5-Fluorouracil (5-FU):

More Likely: Loss of appetite; soreness or painful ulcers of the mouth or throat**;** pain when swallowing**;** diarrhea (loose and frequent stools)**;** constipation**;** nausea (feeling sick to the stomach)**;** vomiting (throwing up); temporary hair loss; decreases in the blood cells produced in the bone marrow, leading to decreased white blood cells, red blood cells and platelets.

Less Likely: Swelling and pain of the hands and feet; thinning of the skin; fingernail changes; redness or increased skin coloring over the veins; skin rash; increased sensitivity to the sun.

Rare: Irritation of the eyes with watery eyes and a scratchy feeling; unsteadiness in walking; dizziness; confusion; chest pain; changes in the heart rhythm.

Leucovorin:

More likely: This drug is a vitamin that is used to increase the effectiveness of 5-FU. The side effects of 5-FU may be made worse, especially the side effects on the small and large intestines.

Rare: Allergic reaction (rash, itching, difficulty breathing, low blood pressure); seizures; fainting; fever.

Oxaliplatin:

More Likely: Nausea (feeling sick to your stomach); vomiting (throwing up); diarrhea (frequent bowel movements); numbness or tingling in the hands and/or feet (may feel stronger if exposed to cold); feeling of tightness or fullness in the throat that may make it feel like it is difficult to breathe or swallow; soreness or redness where the drug is injected; lowered white blood cell count (may make you more likely to get infections); lowered red blood cell count (may make you feel tired or weak); lowered platelets (may make you more likely to bruise or bleed); rash; fever; shortness of breath; damage to the liver or kidneys; having less and harder bowel movements; pain that could be in the belly, chest, bones, muscles or joints, along the spine and legs; feeling tired all the time; mouth sores or sore throat that make it difficult or painful to swallow; headache; loss of appetite; trouble sleeping; hearing loss.

Less Likely: Pain and the risk of infection where the drug is injected; inflammation or infection of the bowel; high blood pressure; chills or shaking; swelling in the arms and legs; changes in taste; upset stomach, heartburn, gas; dry mouth; hot flashes or flushing (redness of face and neck); runny nose; cough; hiccups; decreased fluid in

the body because of diarrhea or inability to drink fluids (called dehydration); fluid collecting in the abdomen; vision changes (blurring) usually brief; changes in your heart beat (rapid heart beat); hair loss; allergic reaction (symptoms vary but difficulty breathing, upset stomach, nausea, vomiting, diarrhea, skin rash, and/or itching are common with allergic reactions).

Rare: Confusion, depression, anxiety, or other mental changes; feeling of imbalance (as if you might fall down), dizziness; abnormal liver function; changes in the salts in the blood stream such as potassium, magnesium, calcium, sodium and phosphorous; inflammation of the bowels, 'blow-out' of the bowels, leakage; blood clots; pain while peeing or blood in the urine, Inability to pee or need to pee frequently; changes in nerve function, including possible confusion, imbalance, lack of coordination, sleep disturbance, eye sight; muscle spasms or loss of normal muscular contraction; abnormal eye muscle movement, fluid in or around the eye; rapid and/or jumpy heartbeat; seizure or passing out; temporary blockage or paralysis of the bowels resulting in abdominal pain and cramping, which may prevent normal bowel movements; hives or itchy skin, dry skin, change in skin color; nail changes; hoarseness, loss or alteration in voice, laryngitis; infection.

Rare but Serious: Hemolytic Uremic Syndrome - low platelets, low red blood cells and kidney failure together; Pulmonary Fibrosis - lung problems such as cough, shortness of breath, trouble breathing, build-up of scar tissue in lungs; thickening and stiffening of lung tissue.

Can be life threatening - tell your doctor right away if you experience any of these problems: bleeding and blood clots; bleeding from any source including stomach (throwing up blood), or black stools; lung (coughing up blood), bowels (blood in the stool) or brain; Veno-occlusive disease – liver injury which leads to an enlarged liver, enlarged spleen, swelling in the abdomen, and jaundice (yellowing of the skin); Tumor Lysis Syndrome- a complication that can occur when cancer cells destroyed by treatmen- may damage kidneys and change calcium level - may lead to kidney dialysis, usually on a short-term basis.

Nausea Medications: Most chemotherapy drugs can cause nausea and vomiting. If you have severe nausea and vomiting, you may have to stay in the hospital. Many types of drugs can be given to help or prevent nausea and vomiting. These types of drugs can cause the side effects listed below.

Common: You may feel weak, drowsy, restless, dizzy, have poor balance or a hard time using your hands or feet and judgment. You should not drink alcohol, use machines, or drive for at least 24 hours after taking this type of drug.

Uncommon: You may have feelings that are not normal for you, dry mouth, feel depressed, or be confused.

Rare: You may have an allergic reaction to the drugs you get or you may have muscle movements you can't control

Blood draws: Taking a blood sample can cause pain, redness, soreness, bruising, or infection may occur at the needle stick site. Rarely some people faint. The study doctor may put some cream (called EMLA) on your skin to numb the area so you will not feel the needle stick as much. The numbing cream may make your skin or the area have a change in skin color, but this is rare.

4. **Risks that are not known:**

   The chemotherapies that are being given as part of this study are commonly used and we have listed the known side-effects for you. However on rare occasions, there may be risks that we do not know about at this time and that we could not anticipate.

5. **Payment in case you are injured while in this study:**

   In the case of injury or illness resulting from this study, emergency medical treatment is available but will be provided at the usual charge. Although no funds have been set aside to compensate you in the event of injury or illness related to the study treatment or procedures, you do not give up any of your legal rights for compensation by signing this form. You or your insurance company will be charged for continuing medical care and/or hospitalization.

6. **Good effects that might result from this study:**

   a) The benefits to science and humankind that might result from this study are: We may discover ways to better decide which cancer treatments are best for people with your kind of cancer.
   b) The benefits you might get from being in this study are: A more effective treatment for your cancer may be given to you rather than if your cancer treatment is selected at random.

7. **Other treatments you could get if you decide not to be in this study:**

   There are several available chemotherapy treatments for cancers of the stomach and lower esophagus. You can even get these same study treatments even if you decide not to participate in the study. Your doctor can go over the available treatment options and help you to decide what is best for you.

8. **Payments for your time spent taking part in this study or expenses:**

   You will receive no payment for taking part in this study. If you need assistance to help with some of the costs for travel to the clinic please let your doctor know because we may be able to provide assistance for you.

9. **Reasons why the study doctor may take you out of this study:**

   - growth of the cancer
   - you cannot tolerate treatment
   - your doctors feel that the risks of continuing on the protocol therapy are too great
   - you are unable to comply with the study guidelines for treatment and follow-up
   - you become pregnant or start to breast-feed

10. **What will happen if you decide to stop being in this study?**

If you decide to stop being part of the study, you should tell your study doctor. Deciding to not be part of the study will not change your regular medical care in any way.

11. Who to call for any questions or in case you are injured:

If you should have any questions about this research study or if you feel you have been hurt by being a part of this study, please feel free to contact **Dr. Laura Williams Goff** at **(615) 322-4967**. If you cannot reach the research staff, please page the study doctor at **(615) 835-9577.**

For additional information about giving consent or your rights as a person in this study, please feel free to call the Vanderbilt University Institutional Review Board Office at (615) 322-2918 or toll free at (866) 224-8273, or email at http://mcapps01.mc.vanderbilt.edu/IRB/WkshpReg.nsf/Suggestion Form?OpenForm.

12. Confidentiality:

Privacy of Protected Health Information:
All efforts, within reason, will be made to keep your protected health information (PHI) private. PHI is your health information that is, or has been gathered or kept by Vanderbilt as a result of your healthcare. This includes data gathered for research studies, and can be traced back to you. Using or sharing ("disclosure") such data must follow federal privacy rules. By signing the consent for this study, you are agreeing ("authorization") to the uses and likely sharing of your PHI. If you decide to be in this research study, you are also agreeing to let the study team use and share your PHI as described below.

As part of the study, Dr. Goff and her study team may share the results of your study and/or non-study linked blood test results and radiology test results as well as parts of your medical record, to the groups named below. These groups may include people from the Federal Government Office for Human Research Protections, the Vanderbilt University Institutional Review Board, the study supporter, Sanofi-Aventis, other participating investigators at other institutions and possibly your health insurance company Federal privacy rules may not apply to these groups; they have their own rules and codes to assure that all efforts, within reason, will be made to keep your PHI private.

The study results will be kept in your research record for at least six years after the study is finished. At that time, the research data that has not been put in your medical record will be archived. Any research data that has been put into your medical record will be kept for an unknown length of time.

Unless told otherwise, your consent to use or share your PHI does not expire. If you change your mind, we ask that you contact Dr. Goff in writing and let her know that you withdraw your consent. Her mailing address is 777 Preston Research Bldg. Nashville, TN 37232-6307. At that time, we will stop getting any more data about

you. But, the health data we stored before you withdrew your consent may still be used for reporting and research quality.

If you decide not to take part in this research study, it will not affect your treatment, payment or enrollment in any health plans or affect your ability to get benefits. You will get a copy of this form after it is signed.

STATEMENT BY PERSON AGREEING TO BE IN THIS STUDY

I have read this consent form and the research study has been explained to me verbally. All my questions have been answered, and I freely and voluntarily choose to take part in this study.

___________ ____________________

Date Signature of patient/volunteer

Consent obtained by:

___________ ____________________

Date Signature

____

Printed Name and Title

APPENDIX A

Performance Status Criteria

ECOG Performance Status Scale		Karnofsky Performance Scale	
Grade	Descriptions	Percent	Description
0	Normal activity. Fully active, able to carry on all pre-disease performance without restriction.	100	Normal, no complaints, no evidence of disease.
		90	Able to carry on normal activity; minor signs or symptoms of disease.
1	Symptoms, but ambulatory. Restricted in physically strenuous activity, but ambulatory and able to carry out work of a light or sedentary nature (e.g., light housework, office work).	80	Normal activity with effort; some signs or symptoms of disease.
		70	Cares for self, unable to carry on normal activity or to do active work.
2	In bed <50% of the time. Ambulatory and capable of all self-care, but unable to carry out any work activities. Up and about more than 50% of waking hours.	60	Requires occasional assistance, but is able to care for most of his/her needs.
		50	Requires considerable assistance and frequent medical care.
3	In bed >50% of the time. Capable of only limited self-care, confined to bed or chair more than 50% of waking hours.	40	Disabled, requires special care and assistance.
		30	Severely disabled, hospitalization indicated. Death not imminent.
4	100% bedridden. Completely disabled. Cannot carry on any self-care. Totally confined to bed or chair.	20	Very sick, hospitalization indicated. Death not imminent.
		10	Moribund, fatal processes progressing rapidly.
5	Dead.	0	Dead.

APPENDIX B

NATIONAL CANCER INSTITUTE
COMMON TERMINOLOGY CRITERIA FOR ADVERSE EVENTS 3.0 (CTCAE)
PUBLISH DATE JUNE 10, 2003

The electronic version can be found at: http://ctep.cancer.gov/forms/CTCAEv3.pdf

A hard copy will be provided to all study sites.

APPENDIX C

sanofi-aventis U.S. Inc.
Global Pharmacovigilance and Epidemiology
200 Crossing Boulevard, P.O. Box 6890
Mailstop BX4-412-i
Bridgewater, NJ 08807

sanofi-aventis U.S. Inc.

Fax SAE REPORT INVESTIGATOR SPONSORED TRIALS

Please √ one: ☐ **Eloxatin** ☐ **Taxotere** **Reported to FDA?** ☐ **Yes** ☐ **No**

Fax: 908-231-4827

Date: **Pages:**

From: **Phone:**

IST#:	
Study Title:	
PI Name:	
Reportability:	All serious adverse/events (SAEs) unexpected and possibly related to the use of the Study Drug(s) are reported directly to the FDA in accordance with applicable law, regulations and Study protocol with a copy submitted to sanofi-aventis. If the FDA does not require the sponsor (investigator) to submit SAEs that are unexpected and related, SAE reports should be sent to sanofi-aventis when discovered.<br>The U.S. Package Insert shall be used to define expectedness. All SAEs will be evaluated by the investigator for reportability. Relatedness is assessed using the definitions below.<br>For Comparator Drugs / Secondary Suspects (Concomitant Medications), all serious adverse experiences will be forwarded to the product manufacturer.
Please √ one:	
	Unlikely: The event is clearly due to causes distinct from the use of the study drug, such as a documented pre-existing condition, the effect of a concomitant medication, a new condition which, based upon the pathophysiology of the condition, and the pharmacology of the study drug, would be unlikely related to the use of the study drug.
	Possible: The event follows a reasonable temporal sequence from administration of the study drug or the event follows a known response pattern to the study drug *BUT* the event could have been produced by an intercurrent medical condition which, based on the pathophysiology of the condition, and the pharmacology of the study drug, would be unlikely related to the use of the study drug or the event could be the effect of a concomitant medication.
	Probable: The event follows a reasonable temporal sequence from administration of the study drug and the event follows a known response pattern to the study drug *AND* the event cannot have been reasonably explained by an intercurrent medical condition *or* the event cannot be the effect of a concomitant medication.
	Definite: The event follows a reasonable temporal sequence from administration of the study drug, the event follows a known response pattern to the study drug and based on the known pharmacology of the study drug, the event is clearly related to the effect of the study drug.
